# Cognitive Impairments Induced by Concussive Mild Traumatic Brain Injury in Mouse Are Ameliorated by Treatment with Phenserine via Multiple Non-Cholinergic and Cholinergic Mechanisms

**DOI:** 10.1371/journal.pone.0156493

**Published:** 2016-06-02

**Authors:** David Tweedie, Koji Fukui, Yazhou Li, Qian-sheng Yu, Shani Barak, Ian A. Tamargo, Vardit Rubovitch, Harold W. Holloway, Elin Lehrmann, William H. Wood, Yongqing Zhang, Kevin G. Becker, Evelyn Perez, Henriette Van Praag, Yu Luo, Barry J. Hoffer, Robert E. Becker, Chaim G. Pick, Nigel H. Greig

**Affiliations:** 1 Translational Gerontology Branch, Intramural Research Program, National Institute on Aging, National Institutes of Health, Baltimore, MD 21224, United States of America; 2 Division of Bioscience and Engineering, Shibaura Institute of Technology, Saitama 3378570, Japan; 3 Department of Anatomy and Anthropology, Sackler School of Medicine, Tel-Aviv University, Tel-Aviv, 69978 Israel; 4 Laboratory of Genetics and Genomics, Intramural Research Program, National Institute on Aging, National Institutes of Health, Baltimore, MD 21224, United States of America; 5 Laboratory of Behavioral Neuroscience, Intramural Research Program, National Institute on Aging, National Institutes of Health, Baltimore, MD 21224, United States of America; 6 Laboratory of Neurosciences, Intramural Research Program, National Institute on Aging, National Institutes of Health, Baltimore, MD 21224, United States of America; 7 Department of Neurosurgery, Case Western Reserve University School of Medicine, Cleveland, OH, United States of America; 8 Independent Researcher, 7123 Pinebrook Road, Park City, UT 94098, United States of America; 9 Sagol School of Neuroscience, Tel-Aviv University, Tel-Aviv, 69978 Israel; University of Florida, UNITED STATES

## Abstract

Traumatic brain injury (TBI), often caused by a concussive impact to the head, affects an estimated 1.7 million Americans annually. With no approved drugs, its pharmacological treatment represents a significant and currently unmet medical need. In our prior development of the anti-cholinesterase compound phenserine for the treatment of neurodegenerative disorders, we recognized that it also possesses non-cholinergic actions with clinical potential. Here, we demonstrate neuroprotective actions of phenserine in neuronal cultures challenged with oxidative stress and glutamate excitotoxicity, two insults of relevance to TBI. These actions translated into amelioration of spatial and visual memory impairments in a mouse model of closed head mild TBI (mTBI) two days following cessation of clinically translatable dosing with phenserine (2.5 and 5.0 mg/kg BID x 5 days initiated post mTBI) in the absence of anti-cholinesterase activity. mTBI elevated levels of thiobarbituric acid reactive substances (TBARS), a marker of oxidative stress. Phenserine counteracted this by augmenting homeostatic mechanisms to mitigate oxidative stress, including superoxide dismutase [SOD] 1 and 2, and glutathione peroxidase [GPx], the activity and protein levels of which were measured by specific assays. Microarray analysis of hippocampal gene expression established that large numbers of genes were exclusively regulated by each individual treatment with a substantial number of them co-regulated between groups. Molecular pathways associated with lipid peroxidation were found to be regulated by mTBI, and treatment of mTBI animals with phenserine effectively reversed injury-induced regulations in the ‘Blalock Alzheimer’s Disease Up’ pathway. Together these data suggest that multiple phenserine-associated actions underpin this compound’s ability to ameliorate cognitive deficits caused by mTBI, and support the further evaluation of the compound as a therapeutic for TBI.

## Introduction

Traumatic brain injury represents a significant and growing cause of disability and death worldwide, and is estimated to become the third largest cause of global disease burden by 2020 [1]. Every year, some 10 million individuals succumb to TBI events that can be broadly subdivided as either concussive or blast in origin. The former type of TBI is far more common, and is exemplified by events such as automobile accidents, full contact sporting injuries and falls in the young and, particularly, in the elderly. The latter type of TBI, generally resulting from a high-pressure shock wave from an explosive device, are a prevalent form of injury in modern combat arenas that primarily affect servicemen/women and civilians in active war zones. In addition to immediate TBI-induced physical injuries, ensuing secondary damage, and signature cognitive deficits, TBI is increasingly considered to be an important conduit to the development of chronic neurodegenerative disorders [2]. In this regard, a strong association between TBI and the onset of dementia-related illness has recently been reported in US military veterans [3,4]. This finding is of particular significance in the face of estimates that approximately 15% of all deployed military personnel receive a mild to moderate TBI of one form or another, with the total number of such injuries estimated as high as 320,000 [5,6]. TBI has also been associated with an increased likelihood of Parkinson’s disease [7], particularly in the elderly. Within the US alone, it is estimated that at least 1.7 million people suffer a TBI event annually. Studies suggest that every year approximately 235,000 TBI victims require hospitalization that can, in the most severe instances, result in some 50,000 deaths annually. Indeed, at least 5.3 million Americans are presently believed to be living with long-term disabilities associated with a TBI [8], for which there is currently no effective pharmacological treatment.

Neurological sequelae of TBI events include shearing and compression of neuronal and vascular tissue that, in turn, initiate a secondary cascade of pathological events that cause further brain injury. Secondary injury to brain tissues may be amenable to therapeutic intervention. Irrespective of the type of injury, mild to moderate TBI can lead to headaches, sleep disorders, and significant impairments across a broad range of brain functions such as attention, cognition, emotion and social behavior [9,10]. Clinical and experimental research suggests that mTBI-induced cognitive changes may be derived from learning and memory deficits caused by damage to the hippocampus and hippocampal-dependent functions [11,12] from oxidative stress, neuroinflammation, ischemia, neuronal degeneration, elevated excitatory neurotransmitters (e.g., glutamate excitotoxicity), loss or disruption of synaptic connections, or altered synaptic physiology [13–17].

Advances in our understanding of the molecular mechanisms that regulate the health and survival of neurons in neurodegenerative conditions has allowed identification of key biochemical cascades linked to neuronal dysfunction and cellular demise that may be amenable to pharmacologic intervention. Our prior studies using pharmacological tools, such as anti-apoptotic p53 inhibitors [18,19], indicate that neuronal cell death occurring in the secondary phase of injury can be halted to thereby mitigate cognitive deficits; however, such agents are not readily clinically translatable. In contrast, clinically translatable drugs focused towards mechanisms shared between neurodegenerative disorders and TBI have yet to provide unequivocal efficacy profiles supportive of clinical approval.

Studies of experimental TBI models as well as post mortem human TBI samples have demonstrated losses in key features of the cholinergic system [20–23]. Cholinesterase inhibitors have, for example, been appraised in preclinical and clinical TBI studies, but have generated largely mixed results [24–29]. Paradoxically, rapid elevations in acetylcholine (ACh) levels within CSF of animal models and humans have been reported following TBI [30–33], with higher levels associated with greater injury [34]. This trend supported the early experimental and clinical use of anticholinergic agents, particularly muscarinic antagonists, for the mitigation of ACh-related toxicity to ameliorate TBI-induced deficits [35–39].

In the present study we evaluated the actions of an experimental and reversible anti-acetylcholinesterase (AChE) agent, (-)-phenserine tartrate (phenserine) in a well-characterized mild concussive model of TBI in mouse [18,40–43]. Notably, in addition to its anti-AChE activity, phenserine is able to inhibit the synthesis of amyloid precursor protein and alpha-synuclein, proteins of relevance to the pathology of AD and PD, respectively, but of currently unknown relevance to TBI. In addition, phenserine possesses anti-inflammatory properties [44], a phenomenon of significance in TBI, although the majority of anti-inflammatory approaches have failed [45].

We now report that mTBI-induced deficits in two common measures of rodent cognition were substantially ameliorated by the acute systemic administration of phenserine for 5 days, when initiated shortly following induction of TBI. These actions on cognition were non-cholinergically mediated and not symptomatic, as they were assessed 2 days following phenserine treatment cessation and washout. In addition to animal behavioral testing, we undertook cellular studies focused on insults known to be involved in TBI (oxidative stress and glutamate excitotoxicity), as well as biochemical and transcriptomic analyses of hippocampal tissue in order to gain insight into the molecular elements involved in mTBI-induced cognitive deficits, as well as those that may be involved in the phenserine-induced attenuation of these deficits.

## Methods and Materials

### Materials

Phenserine ((-)-phenylcarbamoyleseroline) was synthesized in the form of its water-soluble tartrate salt (>99.9% chemical and 100% (-)- chiral purity) according to published procedures [46]. All other reagents were from Sigma-Aldrich (St. Louis, MO) unless otherwise stated.

### Cell culture

Cell cultures were maintained at 37°C in a humidified incubator with 5% CO_2_ and 95% air. SH-SY5Y cells, obtained from American Type Culture Collection (ATCC) (Manassas, VA), were grown in a 1:1 mixture of Eagle’s Minimum Essential Medium and Ham’s F12 Medium supplemented with 10% heat-inactivated fetal bovine serum (HI-FBS) and 100 U/mL penicillin/streptomycin (Invitrogen, Carlsbad, CA). Culture media was changed every other day and cells were split in a 1:3 ratio every 4 to 5 days (0.25% trypsin, 0.53 mM EDTA solution) or upon reaching approximately 80% confluence. Primary cortical neurons were obtained from embryonic day 15 Sprague-Dawley rats, dissociated by mild trypsination, and equal numbers of cells were seeded onto 24-well plates in plating media (DMEM-12 media containing 2% B27 supplement [Invitrogen], 10% HI-FBS, 0.5 mM L-glutamine, and 25 *μ*M L-glutamate) at a density of approximately 3 *×* 10^5^ cells/well. From day 3 *in vitro* (DIV), cultures were maintained in feeding media (Neurobasal medium containing 2% B-27 supplement [Invitrogen] and 0.5 mM L-glutamine).

### Cell viability assays

Cell viability was evaluated either by the 3-(4, 5-dimethylthiazol-2-yl) -5-(3-carboxymethoxyphenyl) -2-(4-sulfophenyl)-2H-tetrazolium (MTS) assay or by the lactate dehydrogenase (LDH) assay. For the MTS assay, studies were performed in 96-well plates, and cells were serum-deprived (with 0.5% of serum) overnight before pretreatment with phenserine or vehicle for 1 h. Thereafter, cells were exposed to different concentrations of glutamate or H_2_O_2_ for 24 h, the doses of which were selected from prior experiments to attain a significant but sub-maximal cellular loss within the linear portion of the dose-response curve of each (data not shown). A CellTiter 96 Aqueous One Solution Cell Proliferation Assay kit (Promega, Madison, WI) was utilized to measure a formazan product, which is directly proportional to the cell viability. For the LDH assay, studies were performed in 24-well plates, and cells were pretreated with phenserine for 1 h prior to glutamate exposure for 24 h. Samples of conditioned media were collected for quantification of LDH levels (LDH Cytotoxicity Assay Kit, Cayman Chemical), which is an indicator of plasma membrane integrity and cell viability.

### Animal studies

Mouse mTBI studies were conducted at Tel Aviv University, Israel, and within the Intramural Research Program of the National Institute on Aging, Baltimore, MD, USA. Specifically, male Institute of Cancer Research (ICR) mice, 6–8 weeks old (weighing approximately 30 g) were housed 5 to a cage under a 12-hr. light/dark cycle (constant temperature 22 ± 2°C) with food (Purina rodent chow) and water *ad libitum*. Experimental animal protocols were approved by the Ethics Committees of the Sackler Faculty of Medicine (M-12-063) and by the Animal Care and Use Committee of the Intramural Research Program, National Institute on Aging (438-TGB-2016), and were in compliance with the guidelines for animal experimentation of the National Research Council (Committee for the Update of the Guide for the Care and Use of Laboratory Animals, 2011) and the National Institutes of Health (DHEW publication 85–23, revised, 1995). A minimal number of mice were used for the study and all efforts were made to minimize potential suffering.

### Induction of mild traumatic brain injury (mTBI)

Experimental mTBI was induced using the concussive, closed-scalp head trauma device described previously [40–42]. Mice were anesthetized by inhalation of isoflurane in a closed chamber and placed under a metal tube device where the opening was positioned directly over the animal’s head just anterior to the right ear. The animals were held in a manner such that the force of impact to the skull generated anterio-lateral movements without any rotational movements, analogous to those that occur during closed head injury in automobile accidents. The injury was induced by dropping a blunted cylindrical metal weight (30 g), inside the metal tube device (inner diameter 13 mm) from a height of 80 cm. Mice were placed back in their home cage to allow for recovery from the anesthesia and mTBI, immediately following the induction of the injury. Evaluation of body temperature prior to and following mTBI demonstrated no statistically significant change across groups. The potential effects of the weight drop injury were studied 7 days following the trauma. Each mouse was used in a single experiment and for one time point only. Sham control animals were subjected to the same procedure, but without the weight being dropped. Groups of animals were subjected to mTBI or sham procedure, administered either phenserine tartrate or vehicle, and then examined in behavioral or biochemical studies in parallel, as described below.

### Drug treatments

Mice were given a 5-day regimen of either phenserine tartrate (2.5 or 5.0 mg/kg, intraperitoneal (i.p.) in 0.1 ml/10 g body weight) or vehicle (physiological saline) injections, twice daily (every 12 hr.), with the first injection administered 30 min after injury.

### Animal study treatment groups

Mice were separated into the following groups: (i) control animals, where mice underwent no mTBI and were treated with either (a) vehicle (saline: 0.1 ml/10 g body weight) or (b) phenserine (2.5 or 5.0 mg/kg, i.p. *bis in die* [BID]); (ii) mTBI animals, where mice received a mild TBI (30 g) followed by administration of either vehicle or phenserine (PHEN: 2.5 or 5.0 mg/kg i.p., BID) for a total of 5 days. Animal numbers (N) per group are noted in the Figure legends. Behavioral studies were initiated from day 7 onwards, following a 2 day washout period of drug and vehicle.

### Behavioral assessments

#### Novel object recognition (NOR) paradigm

This task was used to evaluate visual recognition memory, as previously described for mice [47], and is based on the innate tendency of rodents to explore new objects within their environment. Assessment of this natural tendency enables a determination of whether an animal is able to discriminate between a familiar object and a novel one. Mice were individually habituated to an open field plexiglass box (59 × 59 × 20 cm size) for 5 min, 48 h prior to the test. During the acquisition phases, two identical objects (termed A and B) were placed in symmetrical positions within the arena, and animals were allowed to explore the environment for 5 min. These objects were suitably heavy and high to guarantee that mice could neither move nor climb over them. Twenty-four hours following this acquisition phase of training, one of the objects (either A or B, randomly) was replaced with a novel one (C), and the animal’s exploratory behavior was again evaluated over 5 min. Exploration of an object was defined as rearing on the object or sniffing it at a distance of less than 2 cm and/or touching it by nose. Successful recognition was manifested by the preferential exploration of the novel object. A discrimination preference index was determined as follows: (time near the new object -time near the old object)/(time near the new object + time near the old object) [12]. Following each session, all objects were cleaned with 70% ethanol to avoid odor recognition.

#### Y maze paradigm

This test was chosen to assess spatial memory. The Y maze was comprised of three identical arms separated by 120° angles and built from black plexiglass [43]. Each arm was 8 × 30 × 15 cm in dimension and differed from one another solely by the presence of different visual cues (a triangle, square, or a circle). One arm was randomly selected as the “start” arm. On the first trial, lasting for 5 min, each mouse was placed into the start arm and one of the two remaining arms was randomly blocked to limit access. In contrast, during the second trial, lasting for 2 min, all arms of the maze were accessible. These two trials were separated by a 2 min interval, during which the mouse was returned to its home cage. The time spent in each of the arms was quantified during the two trial periods. Between trials, the maze was cleansed using a 70% ethanol solution and was then dried to avoid odor recognition. A discrimination preference index was adopted from Dix and Aggleton [12] as follows: (time in the new arm—time within the familiar arm)/(time in the new arm + time within the familiar arm).

### Oxidative stress evaluation in brain

Markers associated with oxidative stress were evaluated within hippocampal tissue obtained from the side of the concussive injury on Days 5- and 14-post injury. Frozen tissue was weighed, placed in 9 volumes of phosphate buffered saline and sonicated (4°C). A portion of each sample was separated for measurement of thiobarbituric acid reactive substances (TBARS), and the remainder was ultracentrifuged (100,000 x g for 60 min, 4°C [Optima TLX Ultracentrifuge, Beckman Coulter, Brea, CA, USA]) for other analyses.

#### Measurement of anti-oxidative enzyme activity

Quantification of superoxide dismutase (SOD) activity was performed using a SOD determination kit (Sigma-Aldrich) according to the manufacturer’s protocol. This method is based on the xanthine oxidase reaction, which induces superoxide. Nitroblue tetrazolium reacts with superoxide and is reduced to diformazan. In the presence of SOD, however, superoxide reacts with SOD and results in production of hydrogen peroxide, causing a consequential decline in diformazan generation. In this assay, SOD activity is expressed as the ratio of decreased diformazan, and was determined at a 440 nm wavelength absorbance by a microplate reader (xMark Microplate Absorbance Spectrophotometer, Bio-Rad Laboratories Inc., Hercules, CA, USA).

Glutathione peroxidase (GPx) activity was determined by monitoring β-nicotinamide adenine dinucleotide phosphate (NADPH) levels by use of a commercial kit (Glutathione peroxidase cellular activity assay kit, Sigma-Aldrich) following the manufacture's protocol. Samples were mixed with reduced glutathione (GSH), glutathione reductase (GR), and NADPH, and then rapidly incubated with *tert*-butyl hydrogen peroxide. Hydrogen peroxide is reduced by GPx, while GSH is oxidized to glutathione (GSSG). GR reduces GSSG to GSH and concomitantly oxidizes NADPH to NADP+. Consequent reductions in levels of NADPH were measured at a 340 nm absorbance every 10 s for 10 min. SOD and GPx activities were expressed in units of activity per mg protein within the samples.

#### Measurement of antioxidant protein expression

Part of the ultracentrifuge supernatant was separated into a second tube and added to radioimmunoprecipitation assay (RIPA)-buffer (Boston Bioproducts, Inc., Ashland, MA) with protease inhibitors (Protease Inhibitors Cocktail 1, 2 and 3; phenylmethanesulfonyl fluoride [Sigma Aldrich]). LDS sample buffer (Thermo Fisher Scientific, Waltham, MA) was added and incubated for 5 min at 95°C. The samples (10 μg) were loaded into polyacrylamide 10–20% gradient gels (Criterion TGX Precast Gels, Bio-Rad Laboratories Inc.) and transferred to nitrocellulose membranes (Bio-Rad Laboratories) using the Trans-Blot Turbo (Bio-Rad Laboratories). The transferred membranes were stained with Ponceau S solution (Sigma-Aldrich) for 5 min and washed with 1% acetic acid. Images of the membranes were taken (Amersham Imager 600, GE Healthcare, Little Chalfont, UK). Ponceau S staining was used for normalization of each specific protein expression. The membranes were washed in PBS containing 0.5% tween-20 (PBS-T) and incubated in blocking solution (PBS-T containing 5% non-fat milk) for 1 h at room temperature (R/T). The membranes were then probed with antibodies specific for the following proteins: anti-superoxide dismutase (SOD) 1 (#ab13498, 1:2000, Abcam, Cambridge, UK), SOD 2 (#ab13533, 1:2000, Abcam), glutathione peroxidase (GPx)1 (#ab22604, 1:1000, Abcam); overnight at 4°C respectively. Bovine anti-rabbit HRP conjugated antibody was used as a secondary antibody at 1:5000 dilution for 1 h at R/T. Chemiluminescent signals were generated by incubation with the detection reagents (ECL Prime Western Blotting Analysis Reagent; GE Healthcare) according to the manufacture’s procedure. The intensities of Ponceau S, SOD1, SOD2 and GPx1 were quantified by ImageJ software (ImageJ 1.48v, National Institutes of Health, Bethesda, MD). Expression ratios were calculated by dividing the SOD1, SOD2 and GPx-1 density values by those of the Ponceau S.

#### Analysis of lipid peroxidation

For analysis of lipid oxidation, TBARS were quantified. Lipid peroxides were measured using Yagi’s method, as previously described with some modifications. Twenty microliters of each sample homogenate was mixed with 20 μL of 5 mM EDTA, 200 μL of 1% phosphate acid, and 500 μL of 0.7% thiobarbituric acid. The mixtures were heated to 100°C in a heating block for 45 min. After cooling in an icebox, samples were incubated with 600 μL of butanol for 3 min. After centrifugation, the upper layers were isolated and the absorbance was measured at 535 nm using a spectrophotometer (Lambda 25 UV/VIS Systems, PerkinElmer, Waltham, MA, USA). TBARS were expressed as mg protein in the samples.

### Gene expression microarray analysis

In the present study we chose to examine hippocampal tissue gene expressions due to the relevance of the hippocampus in learning and memory, its vulnerability to mild concussive TBI, and due to evidence of mTBI-induced hippocampal cell degeneration in this model [16,18,48]. Hippocampal tissue gene expression was assessed on Day 14 post injury on the side of the concussive injury in a series of experiments parallel to the behavioral studies. The mouse treatment groups utilized in the gene expression studies: sham (n = 5), phenserine (PHEN) no injury (n = 5), mTBI (n = 5), and mTBI/PHEN (n = 5).

Extraction of total RNA from hippocampal tissue has been previously described [49,50]. Total RNA was quality-controlled using the Agilent Bioanalyzer RNA 6000 Chip (Agilent, Santa Clara, CA) and labeled using the Illumina® TotalPrep^TM^ RNA amplification kit, according to the manufacturer’s instructions. Illumina SentrixMouse Ref-8, v2 Expression BeadChips (Illumina, San Diego, CA) were hybridized overnight using 750 ng biotinylated aRNA and following posthybridization washes incubated with streptavidin-conjugated Cy3 and scanned at a resolution of 0.54 μm using the iScan array scanner from Illumina. Hybridization data were extracted from the intensity images using Illumina GenomeStudio software, V2011.1.

Hippocampal gene expression profile comparisons were made between the following mouse treatment data sets: mTBI; mTBI/PHEN and PHEN all vs. sham. Bioinformatic methods used were as described previously [49,50]. In brief, raw array chip hybridization image signals were processed to generate normalized data that was then transformed to create Z-scores for each gene. The Z-score transformed data was then utilized to generate a Z-ratio measurement, which allowed for the statistical analysis of the gene expression data sets. We selected significant genes using the following criteria: 1) gene expression changes that had a z-test p value of ≤ 0.05 vs. the control comparison group; 2) the absolute value of Z-ratio was calculated to be ≥ 1.5 vs. the control comparison group; 3) the False Discovery Rate for the genes was ≤ 0.30; 4) the average Z-score over all sample comparisons were not negative and lastly; 5) a one way independent ANOVA test on the sample group p value cut off was ≤ 0.05. Only genes that consistently displayed significant expression changes in all samples from a given treatment group were considered for further statistical analysis. The relationships of significantly regulated genes identified in the various group comparisons are illustrated by Venn Diagram; details of the genes are provided in a supplemental table (S1 Table). Where PHEN treated mTBI animal Z-score and Z-ratio data were compared with mTBI vs. sham data for individual genes or gene sets (i.e. ontology or pathways), values that are illustrated in graphical format are represented as an overall geometric average change in gene/gene set regulation between treatment groups. All data sets underwent Parametric Analysis of Gene set Enrichment analysis (PAGE, [51]) for Gene Ontology. Regulation of canonical and non-canonical pathways was assessed by use of Ingenuity Pathway Analysis (IPA, Ingenuity Systems, Inc. Redwood City, CA), which enabled the identification of treatment effects on the Broad Institute Pathway gene set data. Raw and z-score normalized data are accessible through Gene Expression Omnibus, Accession Number GSE44625 (http://www.ncbi.nlm.nih.gov/geo/query/acc.cgi?acc=GSE44625).

### Array validation by quantitative (q)-RT-PCR

The same hippocampal RNA samples utilized for cDNA gene array studies were used to validate array data by q-RT-PCR; gene expression comparisons were made between mTBI vs. sham samples. The genes of interest (GOI) were *Arc*; *Fos* and *Tmem66*. q-RT-PCR was performed as described in detail elsewhere [49,50]. Due to observed altered gene transcript expressions for β-actin (*ACTB*), α1-actin skeletal (*ACTA1*), α1b-tubulin (*TUBA1b*) and β-tubulin(s) (tubulin, beta 4A class Iva and tubulin, beta 5 class I [*TUBB4* and *TUBB5*]) in the gene array data we chose to normalize q-RT-PCR data with glyceraldehyde-3-phosphate dehydrogenase (*GAPDH*) as no changes in the expression of this gene transcript were observed with the Illumina array platform used in these studies. The primers used for PCR reactions were as follows for *Arc* (activity-regulated cytoskeleton-associated protein) forward 5’-GGAGGGAGGTCTTCTACCGTC-3’ and reverse 5’-CTGCCCACTGGGTATTTGC-3’ (GenBank accession no. NM_018790.1, Primer Bank 86604725c2, 76 bp); for *Fos* (FBJ murine osteosarcoma viral oncogene homolog) forward 5’-CGGCATCATCTAGGCCCAG -3’ and reverse 5’- TCTGCTGCATAGAAGGAACCG-3’ (GenBank accession no. NM_010234.2, Primer Bank 31560587c3, 82 bp); for *Tmem66* (transmembrane protein 66) forward 5’-CTGGAACGACCCTGACAGAAT -3’ and reverse 5’-AACACACTTCAACTGTGGGATAG -3’ (GenBank accession no. NM_026432.2, Primer Bank 31982647a1, 112 bp), for *Gapdh* forward 5’-AGGTCGGTGTGAACGGATTTG-3’ and reverse 5’-TGTAGACCATGTAGTTGAGGTCA-3’ (GenBank accession no. NM_008084.2, Primer Bank 6679937a1, 123 bp). The validated genes were normalized to the housekeeping gene noted above, and were quantified using the Pfaffl method [52]. Gene expression data are expressed as mean ± standard error of the mean (SEM) of the fold change from sham values. The expression level for the GOI was used for statistical analysis between the appropriate groups (Unpaired students t-test), and p values are provided. The fold change in expression level determined by q-RT-PCR was compared to that obtained from the array analysis.

### Data analysis

All data are presented as mean ± SEM values. Animal behavioral data were analyzed by SPSS 18 software. One-way ANOVA’s were performed to compare among all groups, followed by LSD post hoc tests. *P <0.05. Cell culture and biochemical data were analyzed by Student’s *t*-test (*P < 0.05), with appropriate correction for multiple comparisons.

## Results

### Cellular studies

**Fig 1:**
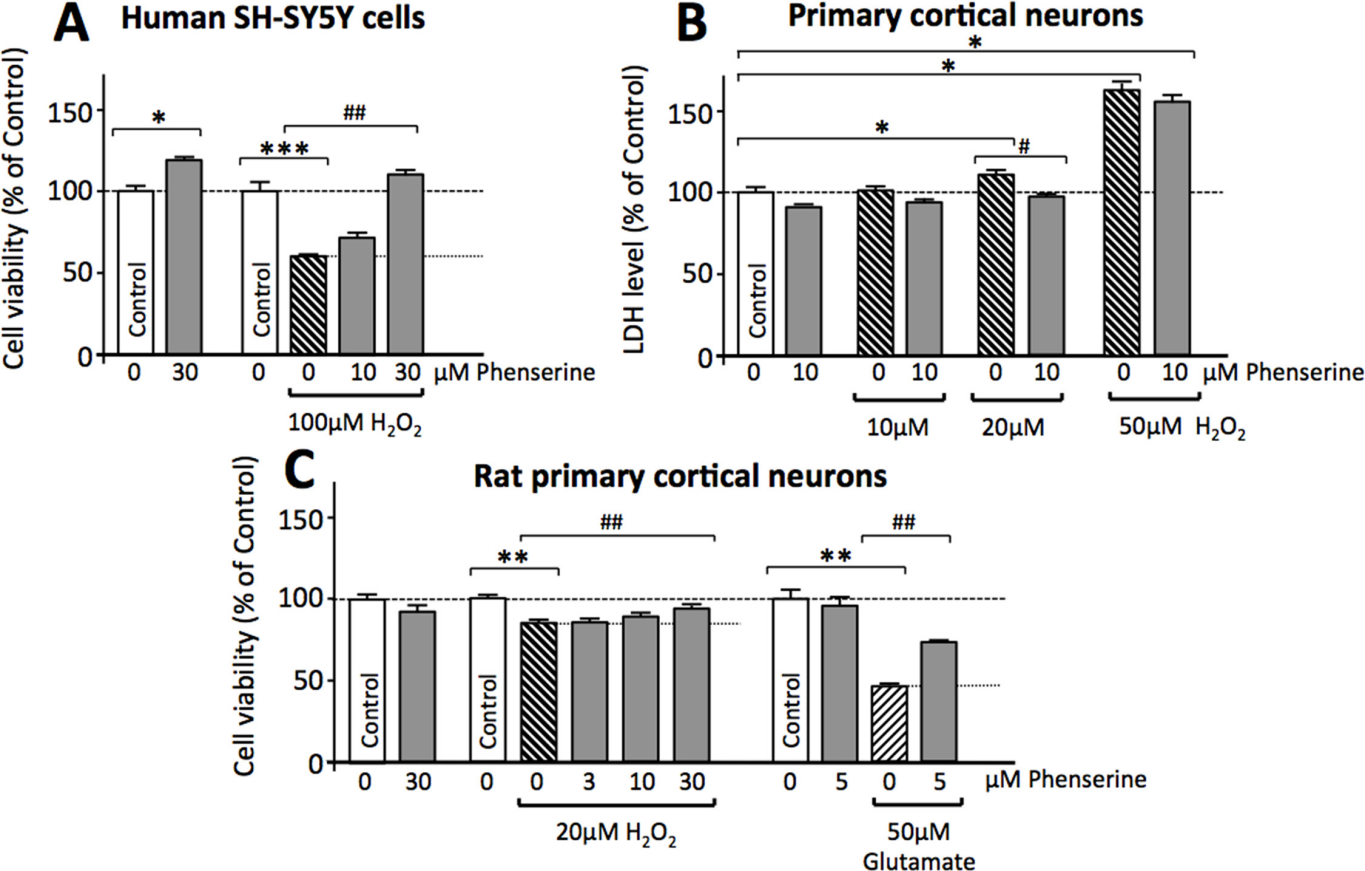
Phenserine mitigates oxidative stress and glutamate excitotoxicity in cultured neuronal cells. (**A**) Human SH-SY5Y cells and rat primary cortical neurons (**B** and **C**) were treated with and without phenserine and challenged with either oxidative stress (H_2_O_2_) or glutamate excitotoxicity. Cell viability was quantified by MTS (**A** and **C**) or LDH (**B**) assay at 24 h. * and # designate comparisons with control cells and either H_2_O_2_ or glutamate challenged cells, respectively (*p<0.05, **p<0.01, ***p<0.001 (comparison with Control cells); #p<0.05, ##p<0.01, (comparison with challenged cells), N≥4 per group.

In light of the occurrence of glutamate excitotoxicity and oxidative stress in TBI [28,53], an immortal cell line and primary cortical cultures were challenged with toxic levels of glutamate and H_2_O_2_ in the presence and/or absence of phenserine to evaluate its neuroprotective potential. Phenserine was well tolerated in human SH-SY5Y cells at concentrations below 100 μM, and proved to be neurotrophic at a concentration of 30 μM, increasing cell viability to 118.6% of control levels (Fig 1A). This same concentration fully protected cells against H_2_O_2_ (100 μM)-induced cell death (Fig 1A). As illustrated in Fig 1B and 1C, rat primary cortical cultures are likewise vulnerable to oxidative stress, with H_2_O_2_ inducing cellular toxicity at 20 μM and above, as evaluated by an increase in LDH levels and a decline in MTS assay-derived cell viability. Phenserine (10 μM and 30 μM, respectively) mitigated the increase in LDH (Fig 1B) and MTS assay-derived cellular loss (Fig 1C) induced by H_2_O_2_ (20 μM). Phenserine levels as low as 5 μM reduced glutamate (50 μM)-induced excitotoxicty (Fig 1C).

### Behavioral assessments

**Fig 2:**
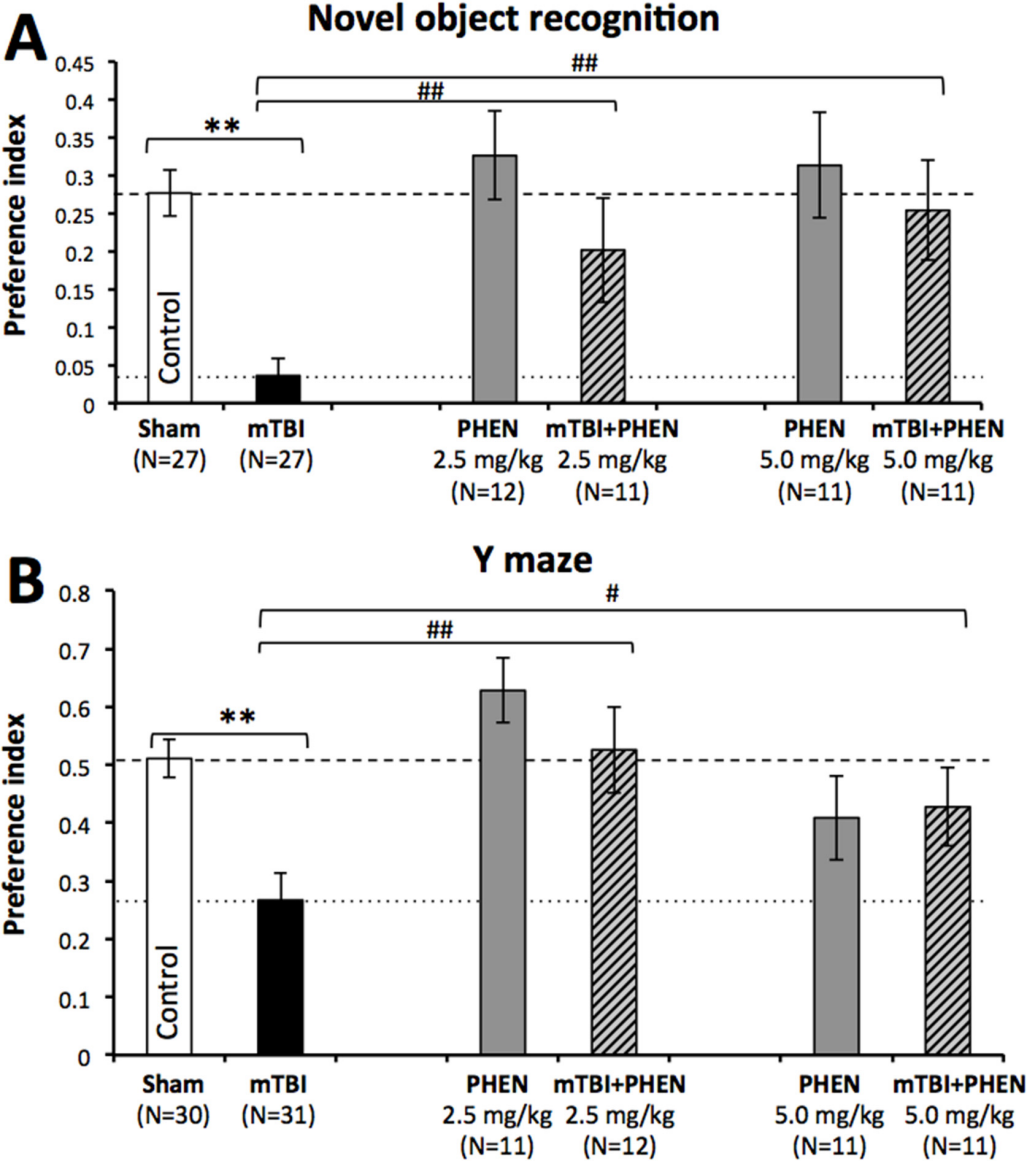
Phenserine mitigates mTBI-induced impairments in visual and spatial memory. (**A**) mTBI mice demonstrate a deficit in visual memory compared with control (**p<0.01). Phenserine administration, at both doses utilized in this study significantly ameliorated behavioral deficits (##p<0.01). One-way ANOVA revealed a significant effect between groups [F(5,98) = 7.770, p = 0.000]. Fisher’s LSD post hoc analysis revealed that the preference index of the “mTBI” group was significantly lower than all other groups (**p<0.01). (**B**) mTBI mice demonstrate a significant deficit in spatial memory compared with control (**p<0.01). Phenserine administration significantly ameliorated this damage (**##**p<0.01 for 2.5mg/kg, and **#**p<0.05 for 5mg/kg). One-way ANOVA revealed a significant effect between groups [F(5,105) = 6.190, p = 0.000]. Fisher’s LSD post hoc analysis revealed that the preference index of the “mTBI” group was significantly lower than all other groups, other than “Phenserine only” 5 mg/kg (*p<0.05, **p<0.01).

In agreement with prior studies [18,49,50], mice subjected to a 30 g concussive mTBI without treatment displayed marked deficits in visual and spatial memory, compared to sham control animals (p<0.01), evaluated by the NOR (Fig 2A) and Y-maze paradigms (Fig 2B), respectively. Administration of phenserine (2.5 and 5.0 mg/kg BID) was well tolerated, and phenserine treated sham animals performed similarly to sham controls in both paradigms, evaluated between days 7 and 14 post injury. Notably, the novel object preference index of mice subjected to mTBI and treated with either dose of phenserine was superior to, and significantly different from, mTBI untreated animals (p<0.05) and no different from that of sham control mice (Fig 2A). A similar amelioration of spatial deficits was induced by both doses of phenserine in the Y-maze paradigm, with the preference index for phenserine-treated, mTBI-challenged mice being significantly higher than untreated mTBI animals, and no different from control sham mice (Fig 2B).

### Oxidative stress

**Fig 3:**
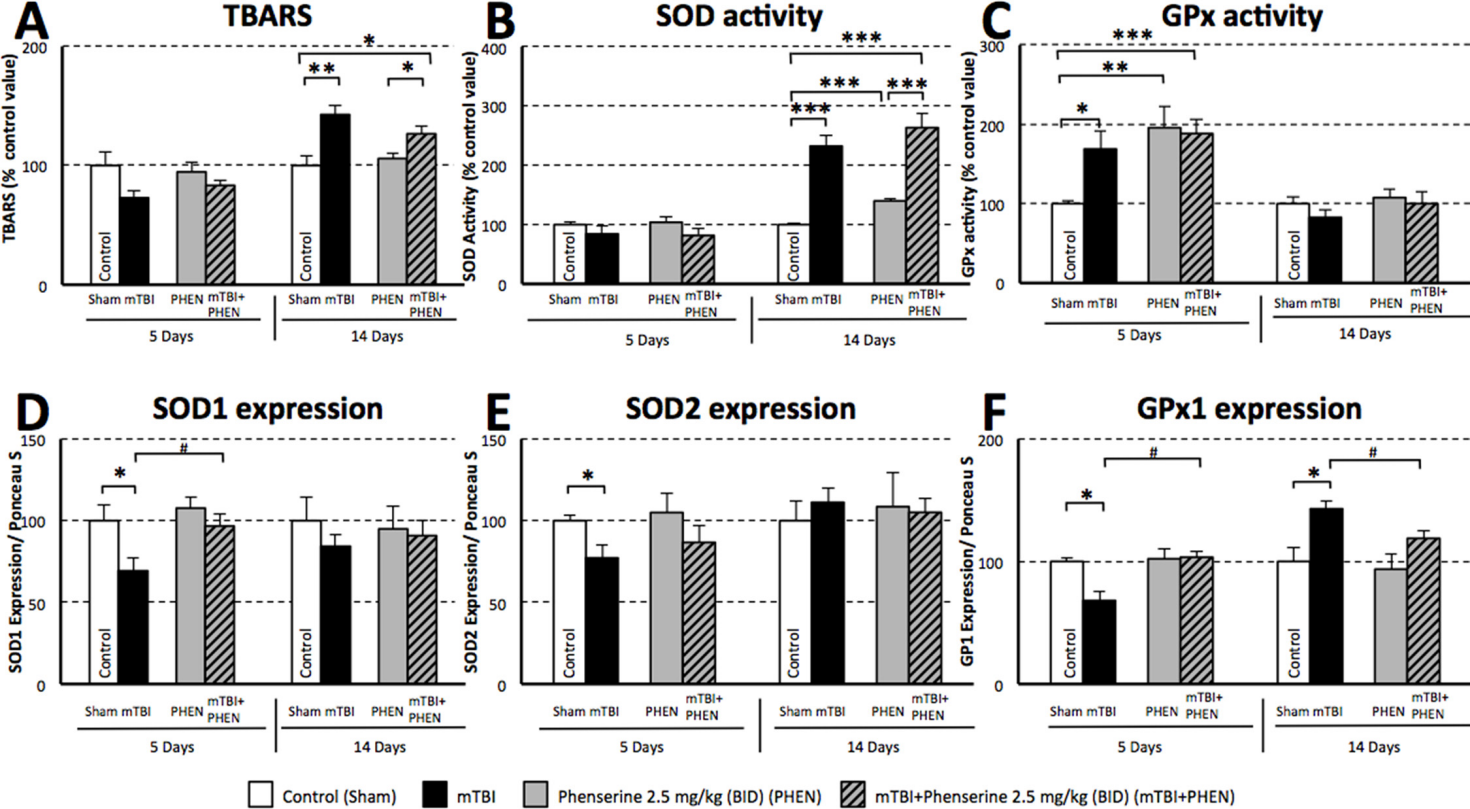
Phenserine augments endogenous antioxidant homeostatic mechanisms relevant to mTBI. Quantification within the ipisilateral hippocampus at 5 and 14 days following mTBI or a sham procedure with and without phenserine (PHEN) treatment: (**A**) levels of thiobarbituric acid reactive substances (TBARS), (**B**) superoxide dismutase (SOD) activity, (**C**) glutathione peroxidase (GPx) activity, and (**D**) SOD1 (**E**) SOD2 and (**F**) GPx1 protein levels (each normalized to total protein, as measured by Ponceau S). Values were significantly different from either control (sham) or phenserine in the absence of mTBI: *p<0.05, **p<0.01, ***p<0.001. Values were significantly different between the untreated and phenserine-treated mTBI challenged groups: #p<0.05, ##p<0.01. N = 6 per group for day 5, and N = 4–6 per group for day 14 evaluation.

In light of the extensive evidence supporting the role of oxidative damage in neuronal cell death induced by TBI [54], markers of oxidative stress (TBARS) and homeostatic mechanisms to mitigate oxidative stress (SOD and GPx) were evaluated in hippocampal tissue ipsilateral to mTBI at 5 and 14 days post injury. As illustrated in Fig 3A, unaltered across groups at 5 days, a significant elevation in TBARS, indicative of lipid peroxidation, was evident in untreated mTBI mice at 14 days post mTBI (142.7% of the control value, p≤0.01). Phenserine treated mTBI animals showed a smaller rise in TBARS levels (126% of control values, p≤0.05).

As a counter measure to oxidative stress, endogenous SOD activity/mg was quantified (Fig 3B) and found maintained at control levels across all groups at 5 days. When evaluated at 14 days, however, an elevation in SOD activity was induced by phenserine treatment in sham animals (139.9% of the control value, p≤0.001) and SOD activity was more substantially elevated in untreated and, in particular, in phenserine treated mTBI groups (232.2% and 263.5% of the control value, respectively, p≤0.001). The levels of the endogenous antioxidant enzymes SOD1 and SOD2 were measured to define the protein amount available within the hippocampus (Fig 3D and 3E). The levels of both SOD1 and SOD2 were significantly reduced in untreated mTBI-challenged mice at 5 days (30.3% and 23.0% lower than sham control values, respectively, p≤0.05), with the former likewise being significantly lower than phenserine treated mTBI mice (p≤0.05). These had returned to control levels when evaluated on day 14. By contrast, both phenserine treated sham and mTBI groups possessed SOD1 and SOD2 amounts similar to control values at both 5 and 14 days.

As illustrated in Fig 3C, GPx activity/mg was significantly elevated at day 5 in both untreated and phenserine-treated mTBI groups (168.9% (p≤0.05) and 188.4% (p≤0.001) of the control value, respectively) and, notably, was elevated within the phenserine only sham group (195.7% of control value (p≤0.01), Fig 3C). In line with the described declines in SOD1 and SOD2 protein levels evident at day 5 in untreated mTBI-challenged mice, these same animals expressed a reduced protein level of GPx1 vs. control shams (-31.5%, p≤0.05), as well as vs. phenserine-treated mTBI animals (-33.8%, p≤0.05) at day 5 (Fig 3F). By 14 days, however, GPx1 protein levels in untreated mTBI-challenged were elevated to 143.2% (p≤0.05) of control values. In contrast, phenserine-treated mTBI mice had expression values similar to both sham control and phenserine control groups at both times.

### Significantly regulated genes

**Fig 4:**
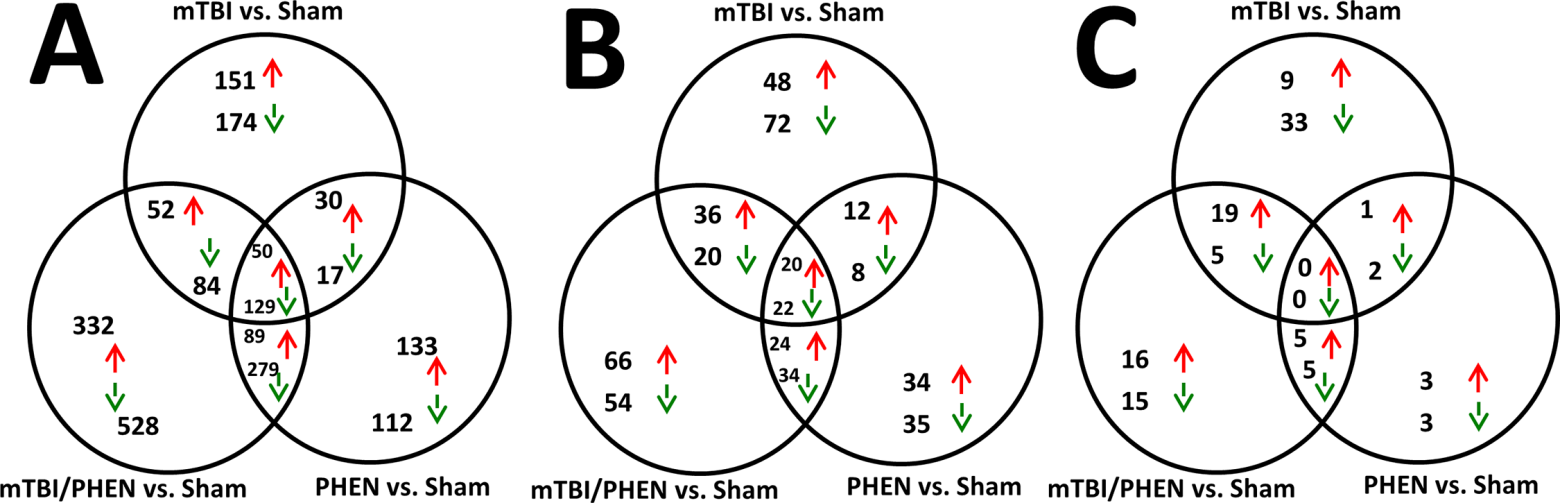
Significantly regulated genes, Gene Ontologies and Canonical Pathways observed in hippocampal tissues from mTBI, mTBI/PHEN and PHEN animals are presented. Red arrows indicate up regulated genes, Green arrows indicate down regulated genes. **A:** Genes: by 14 days after injury, 50 and 129 genes were commonly up and down regulated, respectively, in the three treatment groups. The largest numbers of genes exclusively regulated were observed in the mTBI+PHEN treated animals, 332 genes and 528 genes were exclusively up and down regulated, respectively. Due to the large numbers of genes the identities and Z-ratios of the genes are provided in S1 Table. **B:** Gene ontologies: ontologies derived from altered genes in hippocampal tissues indicate that 20 and 22 gene ontologies were commonly up and down regulated, respectively, in the three treatment groups. Several CNS or neuronal gene ontology listings are described in the results section; however, all ontology identities and Z-scores are provided in S2 Table. **C:** Pathways: molecular pathways derived from altered genes in hippocampal tissues indicate no pathways were commonly regulated in the three treatment groups. Several pathways were CNS- or neuronal-related. These pathways are described in the results section. A list of pathways with corresponding Z-scores is provided in S3 Table.

In an attempt to help define possible molecular players involved in phenserine treatment amelioration of TBI-induced cognitive impairment on a large scale, we examined changes in hippocampal gene groups from mice challenged with mTBI with and without treatment with phenserine. The number of genes significantly regulated by mTBI, mTBI/PHEN and PHEN (all vs. sham samples) are illustrated by Venn diagrams (Fig 4A). A large number of genes were determined to be significantly regulated by the three treatment groups compared to sham samples; the details of which genes were regulated are provided in S1 Table (shown are gene symbol and Z-ratio). The data has been sorted in descending Z-ratio for the mTBI vs. sham treatment group. Upon examination of the most up and down regulated common genes (S1 Table), 13 genes were associated with neurological disorders including seizures, Alzheimer’s disease, brain cancer and amyotrophic lateral sclerosis and neuronitis. The remaining most regulated common genes were linked with the innate immune system, and regulators of transcription.

In addition to commonly regulated genes observed in all three treatment groups, there were numerous genes displaying co-regulation in pairwise comparisons (Fig 4A). Interestingly the largest numbers of genes were observed to be exclusively regulated by each individual treatment comparison with the sham group. The fold changes in transcripts detected in our hippocampal samples were relatively small. The two most highly regulated gene transcripts identified in our arrays, induced by mTBI were as follows: *Cox6a2*, which had a fold change of +2.01 and Z-ratio of +8.48. The product of this gene codes for the cytochrome c oxidase subunit VIa polypeptide 2, which is the terminal enzyme of the mitochondrial respiratory chain where it catalyzes the electron transfer from reduced cytochrome c to oxygen in the generation of ATP. The most highly down regulated gene probe was *Gtf3c1*, the fold change was -1.60 with a corresponding Z-ratio of -6.46. The product of this gene codes for the general transcription factor IIIC, polypeptide 1, alpha 220kD which is involved in mediating gene relocation and transcription to regulate the rearrangement of nuclear architecture in neurons following neuron excitation [55]. In the mTBI/PHEN samples the two most highly regulated genes probes were *Cox6a2* (+2.04 fold change and a Z-ratio of +6.99) and *Deadc1* (also known as *ADAT2*); this gene had a -1.87 fold change and a Z-ratio of -6.90 it codes for adenosine deaminase, TRNA-Specific 2. There is a major role for adenosine dysregulation in TBI [56]. The two most regulated genes induced in PHEN only samples were as follows: *Fos*; this gene had a fold change of +1.86 and a Z-ratio of +7.57. This gene codes for the FBJ murine osteosarcoma viral oncogene homolog and is a component of the transcription factor complex activator protein-1. The gene *Rapgef5*, showed a fold change of -2.13 and a Z-ratio of -10.77. The gene codes for Rap guanine nucleotide exchange factor (GEF) 5.

### Significantly regulated gene ontology

Common themes indicated by CNS related GO data suggest altered physiological processes involved in hippocampal synaptic plasticity. Many Gene Ontology (GO) classifications were observed to be regulated by the treatments at Day 14 after injury (Fig 4B**)**. Forty two of the significantly regulated GOs identified from all three comparisons were of relevance to CNS or neuronal physiology/pathophysiology. The identities of all regulated GOs indicated in Fig 4B are provided in S2 Table. Provided are a GO identifier and Z-scores for the appropriate comparisons; data were sorted based upon decreasing Z-scores of the mTBI vs. sham comparison.

Of the commonly regulated GOs identified in all three comparisons, 10 were CNS or neuronal related and were associated with alterations in glutamate and dopamine signaling in mouse hippocampal tissue indicative of abnormal synaptic plasticity, an altered sense of smell and grooming behaviors. The identities of the GOs are, with positive Z-scores GO0004984 olfactory receptor activity, GO0007608 sensory perception of smell and GO0008188 neuropeptide receptor activity. GOs displaying negative Z-scores are GO0004952 dopamine receptor activity; GO0007212 dopamine receptor signaling pathway; GO0004970 ionotropic glutamate receptor activity; GO0005234 extracellular glutamate gated ion channel and GO0031103 axon regeneration, GO0001975 response to amphetamine and GO0007625 grooming behavior.

CNS-neuronal GOs exclusively altered in the mTBI vs. sham samples but not in the other comparisons were GO0007271 synaptic transmission cholinergic; GO0007399 nervous system development; GO0001540 beta amyloid binding, all had negative Z-scores.

Other changes in dopamine physiology in the mTBI/PHEN samples were associated with a dysregulation in synaptic plasticity; the GOs include GO0050780 dopamine receptor binding; GO0042053 regulation of dopamine metabolic process (both had negative Z-scores) and one GO with a positive Z-score GO0001963 synaptic transmission dopaminergic. Other changes in CNS GOs included GO0007409 axonogenesis and GO0048168 regulation of neuronal synaptic plasticity (both had negative Z-scores).

GOs only regulated in the PHEN treated animals included (with positive Z-scores) GO0007611 learning and or memory; GO0042551 neuron maturation and GO0021513 spinal cord dorsal or ventral patterning. GOs with negative Z-scores included GO0021987 cerebral cortex development and GO0016525 negative regulation of angiogenesis.

### Significantly Regulated Pathways

Canonical pathways associated with neurodegeneration, altered neurotransmission and lipid metabolism were identified in our hippocampal samples. No pathways were commonly regulated by all three treatments (Fig 4C). However there was a pairwise co-regulation of pathways i.e. for the mTBI and mTBI/PHEN comparisons (both vs. sham) and 19 and 5 pathways were co up and down regulated, respectively. The identities and Z-scores of the canonical pathways illustrated in Fig 4C are listed in S3 Table, data were sorted based upon increasing Z-scores of the mTBI vs. sham comparisons.

**Fig 5:**
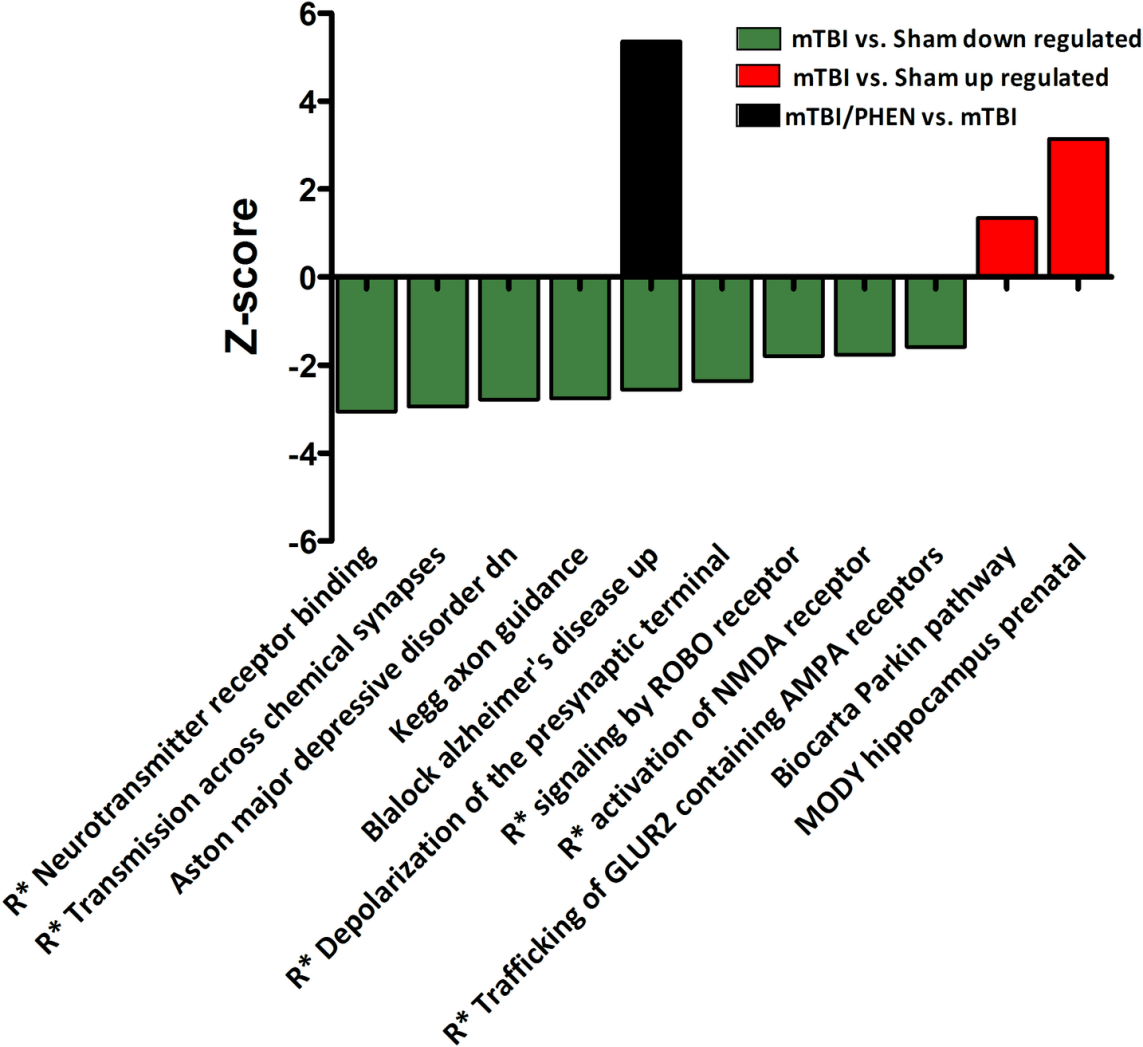
CNS—neuronal canonical and non-canonical pathways observed to be regulated by mTBI compared to sham, and the effects of post-injury treatment with phenserine on mTBI-regulated pathways are presented. R* refers to Reactome. Most pathways were observed to be down regulated by mTBI, with only two that were up regulated. Two pathways were associated with neurodegenerative disease: ‘Blalock Alzheimer’s Disease Up’ and ‘Biocarta Parkin Pathway’. While post-injury treatment of animals with phenserine had no effect on the majority of pathways, one Alzheimer’s disease-related pathway was further regulated by this drug. Drug treatment reversed the effects of mTBI on the Alzheimer’s disease pathway.

Neurodegeneration-related pathways include the Biocarta Parkin Pathway (mTBI vs. sham and PHEN vs. sham, both positive Z-scores); and the Blalock Alzheimer’s Disease Up pathway, which presented with a negative Z-score (mTBI vs. sham). After treatment with PHEN, injury-induced gene expressions for this pathway were reversed i.e. for mTBI/PHEN vs. mTBI for Blalock Alzheimer’s Disease Up pathway the Z-score was positive (Fig 5). Interestingly, other AD-related pathways were observed to be regulated in mTBI/PHEN vs. mTBI samples; Blalock Alzheimer’s Disease Incipient Up (positive Z-score), Blalock Alzheimer’s Disease Incipient Down and Blalock Alzheimer’s Disease Down (both negative Z-scores).

Exclusive mTBI pathways linked with neurotransmission and axon development regulated in our samples include Reactome neurotransmitter receptor binding and downstream transmission in the postsynaptic cell; Reactome transmission across chemical synapses; Reactome depolarization of the presynaptic terminal triggers the opening of calcium channels; Reactome trafficking of GLUR2 containing AMPA receptors, all presented with negative Z-scores. Neuronal developmental pathways altered by mTBI included KEGG axon guidance and Reactome signaling by ROBO receptor, both Z-scores were negative.

Three pathways of note which were observed to be regulated in the mTBI and mTBI/PHEN samples were associated with lipid metabolism. They were Reactome transformation of lanosterol to cholesterol which presented with negative Z-scores in both treatment groups (S3 Table. Significantly Regulated Pathways), Reactome regulation of lipid metabolism by peroxisome proliferator activated receptor alpha (mTBI vs. sham), and Kegg Peroxisome (mTBI/PHEN vs. sham). Interestingly when compared to mTBI samples, the Kegg Peroxisome pathway was further regulated by treatment with PHEN after injury (mTBI/PHEN vs. mTBI, all pathways had negative Z-scores).

### Array validation by quantitative (Q)-RT-PCR

**Fig 6:**
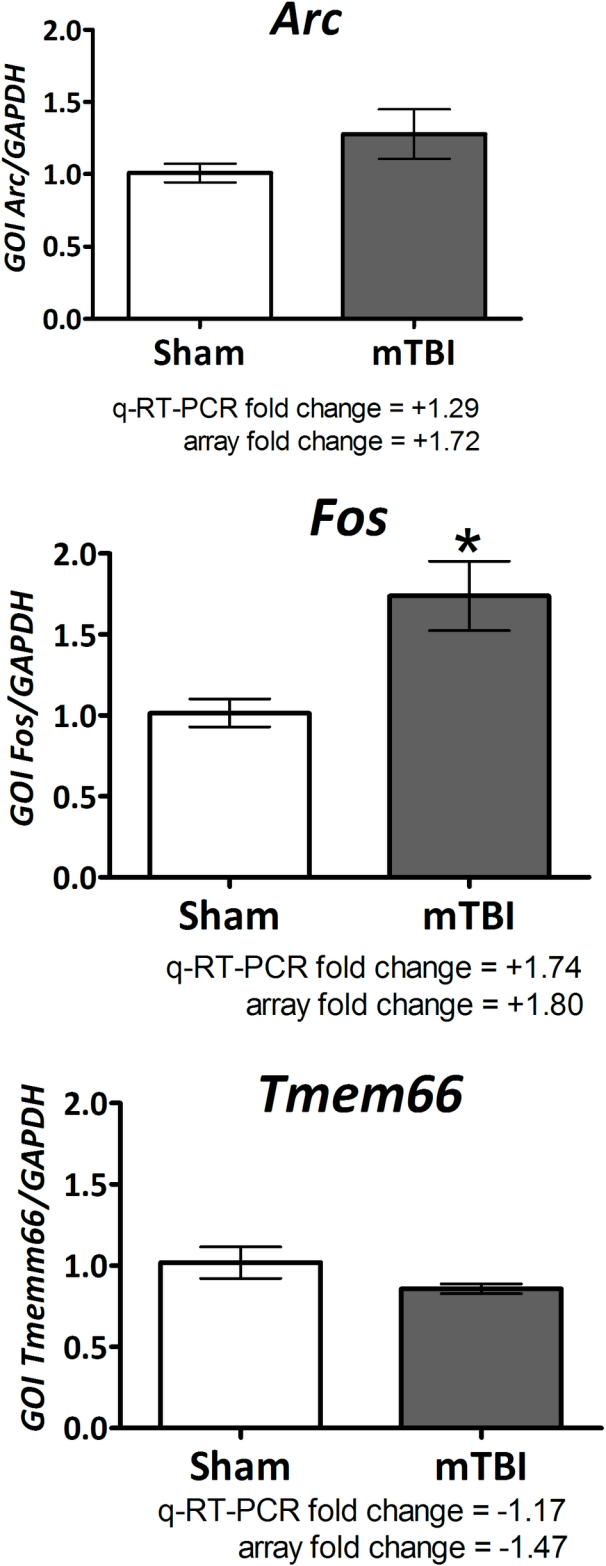
Partial array validation for the genes *Fos*, *Arc* and *Tmem66* illustrate similarities between the fold changes in the gene transcripts measured by q-RT-PCR and array methods. The fold change measurements are indicated in the figure. The mTBI-induced changes for gene transcript levels of *Fos* were significantly elevated compared to sham levels, p = 0.01387. The mTBI-induced changes in transcript levels for *Arc* and *Tmem66* were not significantly different from the sham values (p = 0.1780 and p = 0.1506, respectively); however the fold changes were similar. Values are expressed as mean±SEM of n observations. Sham n = 5 and mTBI n = 5.

Array validation data indicate that of the gene transcripts examined, the fold changes observed between the array methodology and q-RT-PCR methodology were similar. A significant change was observed for the transcript levels of the gene *Fos* in the mTBI samples compared to sham samples (p = 0.01387, both n = 5). The q-RT-PCR and array fold changes were +1.74 and +1.80, respectively. The fold change of the transcript levels of *Arc* and *Tmem66* in mTBI samples were not significantly different when compared to sham transcript levels (p = 0.1780 and p = 0.1506, respectively; both n = 5). However, the q-RT-PCR and array fold changes were similar for both genes; the fold changes for *Arc* were +1.29 and +1.72 and for *Tmemm66* the fold changes were -1.17 and -1.47 (Fig 6).

## Discussion

The incidences of mTBI or concussion are common events and impact approximately 42 million people worldwide each year [7]. Though moderate and severe TBI are established risk factors for neurodegenerative diseases such as dementia, Parkinson’s Disease, chronic traumatic encephalopathy and amyotrophic lateral sclerosis [7,57–59], mTBI has not previously been considered an important risk factor for these disorders. Recent large-scale epidemiological studies, however, have reported that mTBI, which represents the vast majority of TBIs (90%), is actually a significant risk factor in the development of neurodegenerative disorders [3,4,7,60]. Moreover, in the short term, mTBI can cause headaches and meaningful deficits across a wide range of brain functions such as sleep, memory, attention and cognitive processes. mTBI can also cause affective disorders [9]. Such symptoms, which are reported in up to 70% of cases, occur in the absence of overt histological or standard clinical radiological evidence of damage, and may resolve or persist long after injury occurrence. To date, there is no approved drug for the treatment of TBI, despite the evaluation of a broad number of drug classes focused on a range of different mechanisms pertinent to TBI. As a consequence, the effective pharmacological treatment of TBI represents a current and significant unmet medical need. In the present study, we demonstrate that the experimental AD drug phenserine possesses neuroprotective actions in cortical cell cultures challenged with oxidative stress and glutamate excitotoxicity, two insults implicated in the pathogenesis of a wide number of acute and chronic neurological disorders, including TBI [61–63]. Notably, these neuroprotective actions translated significantly into the amelioration of cognitive impairments in a murine, closed-head model of concussive mTBI. Biochemical and gene array evaluation of hippocampal tissue ipsilateral to injury indicated that multiple cellular and molecular processes may underpin the beneficial actions of phenserine in mTBI.

Previous preclinical and clinical studies evaluating a number of anti-cholinesterases in TBI have provided ambiguous results, with some studies reporting improvements and others reporting no activity or further deterioration of cognitive measures [26]. An association between TBI and alterations in cholinergic system function was proposed more than 70 years ago [30]. More recent research indicates that the impact of experimental TBI on cholinergic markers is both diverse and time-dependent. Immediately following TBI (12 min to 4 hr) a marked rise in ACh release and its turnover rate have been described in key cholinergic brain areas [64]. The changes are likely induced by mechanical forces [65]. Declines in the activity of choline acetyltransferase (ChAT) [66] as well as the density of the vesicular acetylcholine transporter (vAChT) [67] have been described within 1 to 2 hr of insult. Reductions in the density of muscarinic and nicotinic ACh receptors have been reported to persist from 2 to 72 hr across cholinergic regions [65], particularly nicotinic α7 receptors that are reportedly decreased within the hippocampus by as much as 50% from 1 hr up to 21 days after TBI [20,67]. Changes in AChE activity have also been characterized, and appear to be both regional and time-dependent. AChE activity increases following TBI in the basal forebrain [68], an area not only notable for its abundance of cholinergic neurons, but also for its role as the origin of the cholinergic innervation to the hippocampus and cortex as well. In contrast, AChE activity is reportedly reduced within these projection areas [68]. Reports of cholinergic neuron loss within both the basal forebrain and hippocampus following TBI have been described in rodents and humans [69–71]. Due to the complex time- and regional-dependent changes in cholinergic function evident in brain following a TBI, and the negative effects on cognitive performance [72] caused by early use of anticholinergics, primarily muscarinic antagonists, to potentially mitigate neurological deficits associated with TBI [31,35–37,73,74], we evaluated the actions of phenserine following a two day washout when cholinergic actions were no longer present, eliminating potential confounding factors.

The action of phenserine was evaluated in a well-characterized mouse mTBI model following two clinically translatable doses of the drug (2.5 and 5.0 mg/kg, BID x 5 days) initiated shortly following injury. These doses are approximately equivalent to 12 and 24 mg in a 60 kg human, following body surface area normalization [75]. This model involves a concussive impact with a weight equivalent to that of the animal (30 g), and parallels collisions and falls in humans, which entail the weight of the victim. Increases in markers of apoptosis (p53, Bax, apoptosis inducing factor [AIF]) have been described in this model, along with some diffuse neuronal loss and neuroinflammation [16,43,48,76,77]. Behavioral evaluation of animals subjected to mTBI was undertaken at 7 days onwards after injury, in accord with previous time-dependent studies [18,43,77]. The assessed behavior involved (i) recognition memory assessed by the classical novel object recognition paradigm and, (ii), spatial memory evaluated by the Y-maze paradigm, both of which were impaired by mTBI in prior studies [18,43,78–80]. The former refers to the ability to discriminate a previously-encountered (familiar) item from a novel one. This task has become a valuable tool in basic and preclinical research for investigating the neural basis of memory [81], and has parallels to visual paired-comparison task studies in humans and monkeys. Damage to the hippocampus is sufficient to produce impaired recognition memory [82–84]. The latter task, spontaneous spatial memory in the Y-maze, is likewise considered a hippocampal-dependent test [84,85]. Importantly, both recognition and spatial memory are impaired in humans with mTBI [86]. The hippocampus appears to be particularly vulnerable to mTBI-induced neuronal degeneration [48,76,87,88].

Analyses of gene expression profiles from affected brain areas in animal concussive mTBI models implicates the involvement of multiple pathways, including those associated with chronic neurodegenerative disorders, epitomized by Alzheimer’s disease and Parkinson’s disease [49,50]. The transcriptome analysis in the current study, likewise, demonstrated multiple gene changes induced by mTBI, phenserine and mTBI/phenserine vs. sham controls, many of which were exclusively regulated by the treatment and many others that were co-regulated. Interestingly, the mTBI/phenserine group displayed the largest numbers of both exclusively regulated as well as co-regulated genes. Two of the genes induced by mTBI that were validated by q-RT-PCR are associated with CNS disorders (Fig 6). *Fos* and *Arc* are linked to schizophrenia, audiogenic seizures, Alzheimer’s disease and autistic syndromes [89–91].

In relation to Gene Ontologies, beyond the previously described disruptions to cholinergic signaling and function that are found in TBI [92,93], implicated in AD [94,95], and associated with amyloid, tau and neuroinflammatory hypotheses in AD progression [96–98], numerous other systems are implicated. Prominent among these systems are the dopaminergic system (with induced changes in dopamine receptor binding [GO0050780], signaling [GO0007212] and activity [GO0004952]), the glutamatergic system (with induced changes in extracellular glutamate gated ion channels [GO0005234] and ionotropic glutamate receptor activity [GO0004970]) and the axonogenesis system [GO0007409]. Interestingly, learning and/or memory [GO0007611] was up regulated in the phenserine sham group, in accordance with the compound’s actions in rodents, canines and humans [99–101].

Pathway analyses demonstrated the involvement of multiple physiological cascades. Many are related to CNS and neuronal systems with many more diverse pathway regulations, including lipid peroxidation, a pathological process identified in this study (Fig 3A). As was observed in a prior study, mTBI-induced regulations in several pathways are associated with CNS and neurodegenerative processes and disorders, namely Alzheimer’s disease and Parkinson’s disease [49]. The findings in the present study extend and confirm these observations with similar Alzheimer’s-related pathways. These pathways were observed to be derived from significantly regulated genes identified in the hippocampal tissue RNA samples. A key pathway of note here is ‘Blalock Alzheimers Disease Up’, which was down regulated by mTBI and oppositely regulated by treatment with phenserine. The identification of mTBI regulated genes in this pathway that were further regulated by treatment with phenserine will provide a focus for further studies to highlight potential proteins of relevance to disease pathology and targets for drug development. It would be interesting to follow phenserine treated mTBI animals for a substantially longer duration in future studies in light of epidemiological studies in humans finding increased risks of Alzheimer’s disease and dementia in those following a head injury [3,4, 102]

At the cellular level, phenserine protected immortal human SH-SY5Y neuronal cells from oxidative stress, and rodent primary cortical neurons from both oxidative stress and glutamate excitotoxicity (Fig 1). These results are in line with previous studies demonstrating the neuroprotective actions of both phenserine and its cholinergically inert enantiomer Posiphen [103], and appear to be mediated via the PKC and ERK pathways. Likewise at a cellular level, there is substantial cross-talk between the cholinergic and glutaminergic systems. Glutamatergic neurons in the hippocampus, cerebral cortex, amygdala and reticular formation form synaptic connections with cholinergic neurons localized within the nucleus basalis of Meynert, the medial septum and diagonal band of Broca. In turn, these cholinergic neurons innervate the hippocampus and neocortex [104]. Glutamatergic signaling has been shown to modulate acetylcholine release, predominantly via the ionotropic *N*-methyl-d-aspartate (NMDA) receptor [105], and some cholinergic basal forebrain neurons appear to release both ACh and glutamate [106]. During normal physiological conditions, NMDA receptor channels are blocked by Mg^2+^ ions. Following a transient glutamate synaptic signal, depolarization of the post-synaptic membrane occurs by release of the voltage-dependent Mg^2+^ channel block to permit Ca^2+^ entry into the post-synaptic neuron and the triggering of a signaling cascade to induce synaptic plasticity and long-term potentiation (LTP) to ultimately augment executive function [104]. NMDA receptor over-activation by excessive glutamate, as is considered to occur in TBI [13–17], leads to sustained Ca^2+^ influx and overload, and generates excitotoxicity—producing neuronal dysfunction and apoptosis [104, 105] to ultimately impact both cholinergic and glutaminergic pathways regulating cognitive functioning. Our cellular studies demonstrate that phenserine mitigates glutamate excitotoxicity to reduce neuronal cell death. Our gene expression studies suggest that phenserine favorably impacts glutamate gated ion channels and glutamate receptor activity at a number of levels in vivo. In addition, it has been reported that physostigmine, for which phenserine bears structural similarity, and select other anticholinesterases have NMDA antagonist activity at albeit it non-clinically relevant concentrations in the order of 50 μM [107]. Further focused studies would be required to define Phenserine mediated mechanism(s) that underpin its neuroprotective actions at the level of glutamate excitotoxicity.

Our analysis of hippocampal tissue from mice suggests that oxidative stress, particularly lipid peroxidation as evaluated by increased levels of TBARS, has a role in mTBI (Fig 3A), which is consistent with previous studies [108,109]. Aberrant changes in mitochondrial function, which include suppression of mitochondrial oxidative phosphorylation, elevation in reactive oxygen species, loss of mitochondrial membrane potential and impaired calcium buffering, have been reported to play critical roles in initiating post-injury neuronal dysfunction and death [110]. Augmentation of antioxidant mechanisms, particularly endogenous pathways, is reported to mitigate ensuing neuronal dysfunction and neuroinflammation in not only TBI [29,111], but also in other neurodegenerative diseases [112,113]. The ability of phenserine to augment the actions of SOD1, SOD2 and GPx *in vivo* may, in part, underlie its favorable actions in mTBI.

Although anticholinesterases have previously been appraised in TBI with variable efficacy [26,114,115], often within a narrow and not readily translatable dose range for human use [116–118], the reversible AChE inhibitor phenserine warrants further evaluation due to its promising actions in the described animal model of concussive head injury that bears face validity to the human condition. Phenserine combines cholinergic actions with valuable non-cholinergic actions; the latter being evaluated following a 2 day drug washout period (in excess of 5 half-lives of the agent’s pharmacokinetic and anticholinesterase actions [119]) to ensure that cholinergic symptomatic actions were not a caveat in evaluating the protection provided by phenserine against mTBI-induced cognitive deficits. Prior experimental and clinical studies with anti-inflammatory, cholinergic and other drugs working via a single mechanism only, may have failed to address the full range of pathologies that lead to neuronal loss and cognitive impairment in TBI and other disorders. Phenserine surprisingly benefits multiple pathways, including the augmentation of endogenous antioxidant, neurotrophic, neuroprotective, anti-inflammatory, pro-angiogenesis [44,103,120–122], as well as cholinergic ones and likely others, too, that provide neuroprotection across animal models, as epitomized by models of nerve gas poisoning and AD [121–123]. Furthermore, the ability of phenserine to lower amyloid precursor protein and alpha-synuclein synthesis [123–125] may be beneficial in relation to TBI-induced pathways that increase the risk for subsequent neurodegenerative disorders. The uncovering of these multiple activities of phenserine over many years illustrates how initial impressions of a drug may mislead investigators away from an agent’s full range of benefits to human health. Phenserine has proven to be well tolerated in humans both acutely [126] and chronically, as assessed in Phase 2 [127] and 3 clinical trials [101] for the treatment of AD. It favorably impacts a number of processes instigated by TBI as well as mechanisms that promote neurorepair for which gaps remain in our knowledge as to how these could be safely and beneficially regulated by other clinically translatable drugs [128], and hence (-)-phenserine warrants further assessment as a new treatment strategy for TBI.

## Supporting Information

S1 Table Significantly Regulated Genes. (DOCX)

S2 Table Significantly Regulated GOs. (DOCX)

S3 Table Significantly Regulated Pathways. (DOCX)
