# Supplementary material for: Cognitive Impairments Induced by Concussive Mild Traumatic Brain Injury in Mouse Are Ameliorated by Treatment with Phenserine via Multiple Non-Cholinergic and Cholinergic Mechanisms
Source: PLoS One. 2016 Jun 2;11(6):e0156493. doi: 10.1371/journal.pone.0156493 (PMC4890804; doi:10.1371/journal.pone.0156493)
Supplement: S1 Table — (DOCX) [file pone.0156493.s001.docx]

**Supplemental Table illustrating the identities of significantly regulated genes indicated in Figure 4 A**

**Common Genes observed in all three treatments**

**mTBI vs. Sham and mTBI/PHEN vs. Sham and PHEN vs. Sham**

**Up regulated Z-ratio Z-ratio Z-ratio**

**Symbol mTBI vs. Sham mTBI/PHEN vs. Sham PHEN vs. Sham**

Fos 7.900 5.250 7.570

Arc 7.380 5.950 6.210

Dusp1 5.440 4.380 5.580

Egr1 4.730 3.380 3.220

Per2 4.260 1.255 3.195

Pfc 4.180 2.360 3.100

Pdlim7 4.140 2.650 3.140

1300002F13Rik 4.000 2.170 2.710

Tra2a 3.990 2.310 2.300

Junb 3.720 4.770 4.950

Clk4 3.210 1.700 2.510

Rps11 2.820 3.440 2.680

Dusp11 2.730 1.780 1.980

Slc38a2 2.700 0.850 1.815

2610207I05Rik 2.690 1.750 2.630

6820449I09Rik 2.670 2.990 3.080

Ankrd12 2.660 1.970 3.100

Dnajb1 2.620 2.030 2.300

Mfge8 2.490 3.460 2.590

Tfg 2.460 1.680 1.630

Rps4x 2.440 2.620 1.800

Mscp 2.430 2.400 2.100

Hrsp12 2.427 2.183 2.800

Rbm26 2.320 1.690 1.650

BC043301 2.210 2.700 2.930

Isgf3g 2.210 1.890 1.640

AA407930 2.120 1.810 2.220

Prpf3 2.010 1.305 1.375

Hp1bp3 1.990 2.500 2.020

Ppm1f 1.990 1.690 1.740

1500031H04Rik 1.920 1.610 1.930

Pik3r1 1.900 1.620 1.650

Adcy6 1.830 1.550 1.600

E2f6 1.810 1.505 2.210

Ddit4 1.800 1.770 1.620

Zfyve21 1.740 2.040 2.000

Pttg1ip 1.710 2.890 2.090

Lmtk2 1.680 1.580 1.690

B430110G05Rik 1.670 1.680 1.770

2810432D09Rik 1.650 2.810 1.760

Gpsn2 1.640 1.860 1.570

Mgst3 1.630 2.150 2.080

Apba3 1.600 2.070 2.720

Gprasp1 1.580 2.370 1.790

Rassf1 1.580 2.430 2.350

Apoa1bp 1.550 1.660 1.820

Caln1 1.530 2.260 2.210

Rpl35 1.520 1.920 1.570

Syngr3 1.510 1.620 2.160

Prkcz 1.255 1.720 1.520

**Common Genes observed in all three treatments**

**Down Regulated Z-ratio Z-ratio Z-ratio**

**Symbol mTBI vs. Sham mTBI/PHEN vs. Sham PHEN vs. Sham**

Acaa2 -1.520 -2.470 -1.620

Pih1d1 -1.540 -1.840 -2.110

BC028440 -1.550 -2.930 -2.000

0610041B22Rik -1.600 -2.450 -2.490

Pbp -1.610 -2.340 -2.840

Tssc4 -1.620 -1.570 -1.860

Zfp179 -1.630 -3.230 -3.740

Paf1 -1.720 -1.970 -1.820

Dusp16 -1.740 -2.360 -1.910

Ephx2 -1.760 -2.220 -2.620

Ugalt2 -1.780 -2.750 -2.650

2310010G13Rik -1.790 -1.950 -2.740

Mkrn1 -1.800 -2.350 -2.080

Ncaph2 -1.810 -2.150 -2.580

Igsf11 -1.850 -1.620 -2.030

Ptma -1.870 -3.040 -2.940

AI837181 -1.890 -2.060 -2.200

1110001A12Rik -1.900 -3.010 -2.000

Siah1a -1.900 -2.500 -1.710

Zfp346 -1.900 -2.550 -2.010

A230051G13Rik -1.910 -1.850 -1.960

Pacs1 -1.910 -2.260 -3.120

Nos1ap -1.920 -3.220 -2.120

Sec14l2 -1.920 -2.340 -1.960

Stx6 -1.930 -2.150 -2.470

Zfand2b -1.930 -2.610 -2.310

Tle1 -1.960 -2.675 -2.505

2810428C21Rik -1.980 -2.980 -2.560

Sca10 -2.000 -2.480 -1.910

Nf1 -2.010 -2.260 -1.610

E030041M21Rik -2.020 -2.590 -2.440

Siat5 -2.030 -2.430 -2.150

MGC68323 -2.040 -2.290 -2.290

Tomm70a -2.060 -3.400 -2.810

Gprc5b -2.080 -2.300 -2.520

H1f0 -2.080 -2.610 -3.210

1110001J03Rik -2.090 -2.800 -2.590

Ppp2r5c -2.090 -2.460 -2.790

Dok4 -2.100 -3.190 -2.820

Bcas2 -2.130 -3.740 -4.030

Dcps -2.130 -2.200 -1.890

Pls3 -2.140 -4.470 -3.010

D130060C09Rik -2.170 -2.880 -3.560

Hps6 -2.190 -2.170 -1.810

BC002230 -2.200 -2.290 -2.240

Klhdc3 -2.200 -1.690 -1.610

Tmem183a -2.200 -2.830 -2.620

2310075A12Rik -2.210 -2.890 -2.900

Grcc2f -2.210 -2.770 -2.920

Aptx -2.220 -3.240 -2.140

Gle1l -2.220 -2.950 -2.850

Sox18 -2.220 -2.250 -2.000

Gabarapl2 -2.230 -3.180 -2.660

Podxl -2.230 -2.720 -2.890

Smarcal1 -2.240 -2.310 -1.710

1300001I01Rik -2.270 -1.640 -1.650

Phyhip -2.270 -2.970 -2.840

D15Mgi27 -2.300 -2.340 -2.200

LOC381813 -2.300 -3.490 -2.650

Ankrd34 -2.310 -2.100 -1.830

6030419C18Rik -2.320 -2.750 -2.500

Glrx2 -2.320 -3.620 -2.610

Ywhaq -2.320 -3.550 -3.260

2010200O16Rik -2.360 -3.200 -2.040

Ccdc53 -2.360 -2.520 -1.960

Rmnd5b -2.360 -2.530 -3.070

AI314180 -2.370 -3.410 -1.950

Mrpl16 -2.370 -2.860 -3.200

1110038F14Rik -2.380 -2.500 -2.870

1700023O11Rik -2.420 -2.800 -2.130

C730025P13Rik -2.420 -2.870 -3.370

Supv3l1 -2.420 -1.950 -1.610

1200009C21Rik -2.430 -1.990 -2.670

LOC216963 -2.430 -2.910 -2.330

Slc35c2 -2.430 -3.270 -2.950

Coq3 -2.440 -2.870 -1.880

2410002F23Rik -2.490 -1.610 -2.590

Ran -2.490 -2.790 -3.090

Yeats4 -2.510 -2.240 -1.900

Zfp691 -2.510 -1.710 -2.650

1810009N02Rik -2.520 -2.090 -2.230

4931406I20Rik -2.530 -2.980 -2.270

Myo5b -2.540 -3.300 -4.190

Hist2h3c1 -2.580 -3.750 -3.950

Ndufb10 -2.580 -4.040 -4.700

Srpr -2.590 -2.530 -2.870

Agpat3 -2.620 -3.000 -3.410

Pla2g6 -2.620 -2.350 -2.160

EG665378 -2.640 -1.740 -3.430

0610010E21Rik -2.670 -2.850 -1.630

2700055A20Rik -2.670 -4.010 -3.960

4733401H18Rik -2.670 -3.820 -1.960

5730528L13Rik -2.700 -3.350 -3.830

Cog8 -2.730 -1.700 -1.850

Rnuxa -2.740 -3.250 -2.520

Wdr8 -2.750 -1.500 -2.390

Lrrn2 -2.760 -5.590 -3.930

Coq2 -2.770 -2.480 -2.660

Mtch2 -2.790 -1.660 -1.510

2010100O12Rik -2.880 -2.580 -2.920

Cenpb -2.880 -1.570 -2.150

Pnma1 -2.950 -2.580 -2.030

8430432M10Rik -2.980 -2.390 -2.380

Cog2 -3.000 -2.320 -2.120

P2ry5 -3.070 -2.740 -1.970

Prpf40b -3.090 -2.810 -2.590

Kcnj4 -3.110 -2.820 -3.170

Zfhx1b -3.120 -5.030 -5.250

Hirip5 -3.190 -3.890 -3.670

Ppp2r4 -3.200 -3.010 -2.490

2900046G09Rik -3.210 -3.020 -2.540

BC085271 -3.210 -2.240 -2.740

Unc5b -3.210 -2.240 -2.470

Zfp297 -3.280 -2.730 -3.250

Pfkl -3.380 -2.550 -2.410

9030425E11Rik -3.440 -3.990 -3.540

Srr -3.450 -3.910 -2.860

Mod1 -3.470 -3.900 -3.240

Fhl2 -3.740 -3.260 -4.330

Tada2l -3.790 -3.210 -3.490

Pacsin1 -3.860 -2.020 -1.810

Ube3a -3.880 -4.020 -3.240

Ephb2 -3.950 -2.800 -2.570

Rps3a -4.300 -5.080 -5.440

Tgfb2 -4.570 -5.830 -5.610

Fus -4.630 -2.060 -1.990

S100a13 -5.690 -2.850 -3.670

Habp4 -6.070 -5.110 -5.140

Gtf3c1 -6.460 -3.040 -3.940

**Common Genes observed in pair wise comparisons**

**mTBI vs. Sham and mTBI/PHEN vs. Sham**

**Up regulated Z-ratio Z-ratio**

**Symbol mTBI vs. Sham mTBI/PHEN vs. Sham**

Cox6a2 8.480 6.990

Mglap 6.220 4.470

Plekhb2 3.790 2.450

Rpl9 3.780 3.140

Mt1 3.700 2.250

Gmip 3.560 2.270

Rplp1 3.390 2.930

Rnf19 3.170 1.640

1810008A18Rik 3.060 3.490

Srfbp1 2.990 1.540

E130016E03Rik 2.980 2.430

Rps6 2.880 2.450

EG668668 2.870 2.410

Rab28 2.860 1.760

Ubl5 2.720 2.330

Ppp1r1c 2.680 1.980

Farp1 2.660 2.470

Rps20 2.530 2.080

Zfp36l1 2.420 2.300

Morf4l2 2.400 1.750

Clu 2.360 2.870

Zranb2 2.350 1.840

Fau 2.340 2.380

4432405B04Rik 2.310 2.060

Cox17 2.310 1.910

Mat2a 2.280 2.750

Eif3s2 2.270 1.780

Atp6v0e 2.230 2.290

Atpif1 2.200 2.700

1110020P15Rik 2.165 2.350

Rpl37 2.140 2.270

Lrpap1 2.130 1.940

Rps13 2.120 2.730

Prdx2 2.080 2.610

Akr1a4 2.000 2.260

Pfdn5 1.950 1.670

Nudc 1.910 2.040

D5Ertd585e 1.900 1.700

Tmed10 1.900 1.850

Acot7 1.860 1.980

A130092J06Rik 1.830 1.780

5033425B17Rik 1.790 1.950

Zfp292 1.790 1.580

5033414D02Rik 1.680 1.700

Tulp4 1.640 1.530

Cyc1 1.630 1.580

Fez1 1.600 1.775

Rps8 1.570 1.840

Mgea6 1.495 1.330

3010033P07Rik 0.973 0.787

Ttc3 0.657 0.257

Vti1b 0.090 -1.035

**Common Genes observed in pair wise comparisons**

**Down Regulated Z-ratio Z-ratio**

**Symbol mTBI vs. Sham mTBI/PHEN vs. Sham**

H2afy -1.540 -1.500

Zdhhc9 -1.560 -1.610

Usp39 -1.580 -1.800

Zscan21 -1.580 -1.870

2310046O06Rik -1.620 -2.260

Med19 -1.620 -1.550

Rasgrp3 -1.690 -2.100

Rasa2 -1.700 -1.700

Scamp3 -1.740 -1.830

Prune -1.760 -2.130

AI790326 -1.790 -1.880

D630041K24Rik -1.790 -2.410

Zdhhc3 -1.790 -2.010

Nucb1 -1.800 -1.860

2610528E23Rik -1.820 -1.720

Kcnmb4 -1.820 -1.630

Mcph1 -1.820 -2.300

Arfip2 -1.850 -1.550

Pitpnc1 -1.850 -1.560

Scarb2 -1.890 -1.830

Klc2 -1.910 -1.560

Rab3ip -1.930 -2.170

Tk2 -1.930 -1.970

1200009B18Rik -1.950 -2.940

Gcs1 -1.950 -2.580

5730410I19Rik -1.960 -1.530

Grip1 -1.960 -1.590

Psmb5 -1.960 -3.270

Fbxl3a -1.980 -2.430

Lrfn2 -1.990 -1.580

Kcnk2 -2.000 -3.450

Mapre3 -2.010 -2.100

Doc2b -2.020 -2.820

Gstm4 -2.020 -1.620

Chrnb2 -2.050 -2.440

Psmd1 -2.060 -1.780

0610011N22Rik -2.070 -1.650

Cno -2.070 -2.670

Mrps25 -2.080 -2.260

5430431G03Rik -2.120 -1.720

5730409E04Rik -2.130 -1.780

Tusc2 -2.140 -1.640

Syngr1 -2.150 -2.040

Fuk -2.160 -2.910

Zdhhc12 -2.210 -1.640

2010316F05Rik -2.220 -1.540

Denr -2.280 -2.010

Ramp2 -2.280 -1.610

Tesc -2.300 -2.550

Znhit4 -2.300 -2.120

Dazap1 -2.330 -2.010

Aarsd1 -2.430 -2.340

Centb5 -2.430 -1.930

3110082I17Rik -2.460 -1.680

Pcdhac2 -2.460 -1.590

2210016L21Rik -2.500 -1.830

Tcof1 -2.500 -1.540

2900045N06Rik -2.510 -1.720

Apc2 -2.520 -1.760

Tgfbr3 -2.530 -2.090

Xpa -2.540 -1.740

A830053O21Rik -2.560 -1.790

Thra -2.560 -2.170

Trappc5 -2.560 -1.560

Rapgef6 -2.610 -3.070

5330408N05Rik -2.780 -2.670

Trps1 -3.040 -1.690

Stx4a -3.050 -1.730

Ndufb6 -3.080 -2.120

Ptms -3.150 -2.330

D030015G18Rik -3.170 -1.580

Atxnl2 -3.200 -1.960

Mansc1 -3.210 -2.670

Rabl3 -3.230 -2.470

5033413H12Rik -3.365 -2.015

0610037L13Rik -3.450 -2.250

Il16 -3.450 -4.170

B3gat3 -3.480 -2.580

Nosip -3.660 -2.050

Atic -3.700 -4.250

Arf5 -3.830 -3.380

Ppp1r14a -3.980 -2.810

Pnpo -4.220 -2.270

Purb -4.330 -3.270

**Common Genes observed in pair wise comparisons**

**mTBI vs. Sham and PHEN vs. Sham**

**Up regulated Z-ratio Z-ratio**

**Symbol mTBI vs. Sham PHEN vs. Sham**

Dok3 5.190 3.530

6330407D12Rik 3.470 2.000

Creld2 3.450 1.620

Mbnl1 3.250 1.630

4933421E11Rik 2.850 2.160

EG622320 2.760 2.260

1200016B10Rik 2.600 2.470

Herpud1 2.360 1.335

Tbp 2.250 2.070

Pcdh17 2.210 2.320

Sh3md4 2.140 1.900

8430410K20Rik 2.130 1.770

Pfdn4 2.130 2.960

Hbp1 2.110 2.390

Als2 2.000 2.530

Pop5 1.980 1.550

4430402O11Rik 1.950 1.930

Trfp 1.890 2.020

1700123O20Rik 1.880 2.120

Cetn3 1.830 2.250

Pnma2 1.800 1.540

Zfp27 1.780 2.290

Sfrs7 1.720 1.800

Itgb1bp1 1.650 1.540

Tpr 1.570 1.530

BC042901 1.550 1.600

Rnf44 1.540 1.530

Rab8b 1.470 1.350

1300018P11Rik 1.250 1.920

Slc25a38 1.237 1.043

**Common Genes observed in pair wise comparisons**

**Down regulated Z-ratio Z-ratio**

**Symbol mTBI vs. Sham PHEN vs. Sham**

H3f3b -1.520 -2.000

Myo18a -1.530 -1.510

Panx1 -1.530 -1.850

2410015N17Rik -1.600 -1.940

Ier5l -1.710 -1.560

Klhl6 -1.890 -2.200

BC018242 -2.170 -1.520

2610029K21Rik -2.220 -3.010

Slc7a10 -2.240 -1.720

Usp4 -2.260 -1.770

Lsm10 -2.460 -1.970

Tap2 -2.520 -2.560

1810049H13Rik -2.600 -1.570

1190002J23Rik -3.280 -2.210

Rrbp1 -3.490 -2.410

Slamf9 -3.820 -2.340

4930455C21Rik -9.900 -6.890

**Common Genes observed in pair wise comparisons**

**mTBI/PHEN vs. Sham and PHEN vs. Sham**

**Up regulated Z-ratio Z-ratio**

**Symbol mTBI/PHEN vs. Sham PHEN vs. Sham**

Actb 5.930 5.670

Dbp 4.160 4.370

Bmp4 4.150 2.840

Hba-a1 3.930 6.410

Garnl3 3.690 3.420

Sox21 3.490 3.410

G431001E03Rik 3.470 4.800

Ier3 3.420 3.280

2700050C12Rik 3.290 2.660

Prkacb 3.290 2.190

Ccnd2 3.230 4.370

Syp 3.160 2.090

Cdh13 3.130 3.820

A930034L06Rik 3.120 4.030

9330134C04Rik 3.110 3.680

Bzrap1 3.110 2.100

Adam15 2.790 2.290

Mbp 2.700 1.935

Timp2 2.690 3.530

Rftn2 2.660 2.140

Garnl4 2.650 2.940

Plagl2 2.640 2.900

Ltbp4 2.580 2.880

Per1 2.565 3.315

Caskin1 2.480 2.620

Map2k3 2.470 2.140

Kcnc4 2.455 1.315

Brd2 2.440 2.260

Rasal1 2.390 3.090

Kifc2 2.370 2.080

Rpl36al 2.330 1.620

Sepn1 2.300 2.510

Lasp1 2.230 1.720

Kif5a 2.220 2.230

Psmb10 2.220 1.610

Scamp2 2.220 1.520

AI132321 2.210 1.890

Midn 2.210 1.800

Ilk 2.190 1.620

Ankrd54 2.180 2.380

Kcnh3 2.180 3.440

BC008155 2.170 1.630

Atp5j 2.150 1.920

Max 2.150 2.500

BC023957 2.140 1.560

Gja1 2.110 1.570

4833420N02Rik 2.100 2.380

Fxyd5 2.070 2.980

Zadh2 2.070 2.370

Cox6b 2.065 1.585

B430214A04Rik 2.010 2.040

D6Wsu176e 2.000 1.610

4632411B12Rik 1.950 2.620

Eno3 1.930 2.580

Maged2 1.920 1.960

2410127E18Rik 1.900 1.910

Smarca2 1.880 1.690

Rabl4 1.870 1.810

Qars 1.850 1.810

Rasl11b 1.820 2.450

Guk1 1.800 1.580

Pdxp 1.790 1.800

A230050P20Rik 1.780 1.880

Notch3 1.780 2.060

2610033C09Rik 1.760 1.600

Traf4 1.755 1.825

Pkd1 1.750 1.630

Rab11fip5 1.750 1.610

Snrpd2 1.740 1.820

Fto 1.730 1.500

DXImx50e 1.720 2.190

Fdps 1.680 1.760

Bckdk 1.640 1.700

Fbxo28 1.630 2.280

Rnf40 1.620 1.560

Zswim3 1.620 2.000

D10Bwg1379e 1.600 2.670

Extl3 1.590 1.250

Pdgfb 1.580 1.580

Zfp316 1.575 2.095

Hspa2 1.570 2.750

Drpla 1.550 1.900

Sema4b 1.550 2.300

Chkb 1.540 1.600

Gcap14 1.540 1.820

1200003C05Rik 1.510 1.710

2700082D03Rik 1.510 1.560

Ube1dc1 1.510 1.870

Kif1b 0.635 1.180

**Common Genes observed in pair wise comparisons**

**Up regulated Z-ratio Z-ratio**

**Symbol mTBI/PHEN vs. Sham PHEN vs. Sham**

Fts -1.500 -2.060

Acadsb -1.510 -1.990

1810057P16Rik -1.520 -2.060

Surf4 -1.520 -1.660

Mrps18c -1.530 -2.580

Coro1c -1.550 -2.360

BC043098 -1.570 -1.580

Acsl1 -1.580 -2.450

Scap -1.620 -1.880

9330187M14Rik -1.630 -1.660

Bmpr1b -1.640 -1.900

Mtmr4 -1.640 -2.050

Klhdc4 -1.660 -2.340

Mki67ip -1.660 -2.760

Tgoln1 -1.660 -1.840

Ptp4a2 -1.670 -2.570

Etv5 -1.680 -1.730

D11Moh34 -1.690 -2.370

Zgpat -1.690 -1.840

Psmd10 -1.700 -2.170

Atp5e -1.710 -2.160

Idh2 -1.720 -2.080

Ddb1 -1.730 -2.350

Ganab -1.730 -1.980

Sez6l2 -1.730 -1.520

Notch1 -1.740 -2.820

Atp13a1 -1.760 -2.710

Pcqap -1.760 -1.590

Aamp -1.770 -1.710

Gmeb2 -1.770 -1.730

Gpatch1 -1.780 -2.750

Rpl36a -1.810 -2.170

Akap2 -1.820 -2.040

2610318I18Rik -1.830 -1.810

5730494N06Rik -1.830 -1.600

Hadhb -1.830 -2.170

Terf2 -1.830 -2.240

Ube2q2 -1.830 -2.490

Tesk1 -1.840 -1.550

Abhd6 -1.850 -1.730

Atf5 -1.850 -2.660

Hcfc1 -1.850 -1.660

Hdh -1.850 -1.880

Ndufaf1 -1.850 -1.730

Plcd1 -1.860 -2.000

Sel1h -1.860 -1.620

Vps33a -1.860 -1.940

2700088M22Rik -1.870 -1.550

1810060J02Rik -1.880 -1.870

Tmem50a -1.880 -2.160

Cdc42 -1.890 -1.920

4931406C07Rik -1.900 -1.840

9630044O09Rik -1.900 -1.820

Myh9 -1.900 -2.620

Ndufb5 -1.900 -2.730

Sra1 -1.910 -2.170

Tgfb1i1 -1.915 -2.820

Rfwd3 -1.920 -1.670

3930402F13Rik -1.930 -1.620

AW060766 -1.930 -1.790

1200015F23Rik -1.940 -2.280

Chga -1.940 -2.280

Uck1 -1.950 -1.770

Aldh6a1 -1.960 -1.560

4930519N13Rik -1.970 -1.970

Mars -1.970 -1.720

Nfyb -1.970 -2.350

4833420K19Rik -1.980 -2.180

Tbc1d23 -1.980 -1.750

Elavl1 -2.000 -1.750

Hcn2 -2.000 -3.140

Mlycd -2.000 -1.980

Rhbdl2 -2.000 -3.030

Mrpl20 -2.010 -2.990

Dhdds -2.020 -1.740

Rtkn -2.020 -1.770

Seh1l -2.020 -2.130

Txndc7 -2.020 -1.950

Rnf167 -2.040 -2.200

Sh3bgrl -2.040 -1.510

Slc23a2 -2.050 -2.040

Tyki -2.050 -3.110

Wbp4 -2.050 -3.270

A930040G15Rik -2.060 -2.070

Itgb5 -2.060 -2.090

Ebpl -2.070 -3.040

Snrpa -2.070 -2.160

Cfdp1 -2.110 -1.600

D10Wsu52e -2.110 -2.130

Gja9 -2.110 -1.710

Ogt -2.110 -1.750

1810008A14Rik -2.120 -1.760

AI481500 -2.120 -1.870

Tmem23 -2.120 -1.910

Pex19 -2.130 -1.520

Armc6 -2.135 -1.550

6720458F09Rik -2.140 -2.350

Itsn1 -2.140 -1.880

Gm440 -2.160 -1.890

Ubl3 -2.160 -1.840

Ylpm1 -2.160 -2.730

Ncbp2 -2.170 -1.680

Tex261 -2.170 -2.530

Cldn12 -2.180 -1.770

Sipa1l1 -2.180 -2.210

Srrp -2.180 -1.570

AW540478 -2.190 -2.670

Trpc4ap -2.190 -2.090

1500002I11Rik -2.200 -2.920

C030034J04Rik -2.200 -2.580

Hmgcl -2.210 -2.210

Zfp289 -2.210 -2.530

BC023829 -2.220 -1.870

Lingo2 -2.230 -2.780

6330548G22Rik -2.240 -2.880

Prpf38a -2.240 -2.120

Aff1 -2.250 -2.150

1810073P09Rik -2.260 -3.050

Scotin -2.260 -2.040

Dnm1l -2.270 -2.100

0610012G03Rik -2.310 -2.410

Bop1 -2.310 -2.760

Slc39a10 -2.320 -1.570

Taf13 -2.320 -1.700

Ppp3ca -2.330 -3.620

Ube2d3 -2.330 -1.790

2700067E09Rik -2.340 -2.250

Rab10 -2.340 -1.820

Hist2h2aa1 -2.350 -3.100

Pcyt1a -2.350 -2.200

Nlrx1 -2.370 -2.700

Trim32 -2.370 -1.600

1500003D12Rik -2.400 -2.670

2810457M08Rik -2.400 -1.560

4631403P03Rik -2.420 -2.030

Zfp428 -2.430 -1.580

Mettl9 -2.460 -1.660

Pgm2 -2.460 -2.310

Vps29 -2.460 -2.080

2010012F05Rik -2.470 -1.970

Cpeb1 -2.470 -2.220

Ncl -2.480 -2.130

Tm9sf2 -2.480 -1.760

Rbm18 -2.490 -2.380

Epm2aip1 -2.505 -2.535

Akr1e1 -2.510 -2.330

D1Bwg1363e -2.510 -1.910

Dixdc1 -2.510 -1.930

Nptx1 -2.510 -1.810

Paip2 -2.520 -2.490

Tbl1x -2.520 -2.310

Cbr3 -2.530 -2.120

Ift20 -2.530 -3.060

Pam -2.530 -3.450

BC005471 -2.540 -2.090

B930006L02Rik -2.560 -3.110

Egln2 -2.560 -3.160

Siat9 -2.570 -1.860

Tsr2 -2.570 -1.900

Adam9 -2.580 -3.090

Sv2b -2.590 -2.210

Rgs14 -2.610 -2.410

4833424O15Rik -2.620 -2.660

Calm2 -2.630 -3.060

Cnn3 -2.640 -2.160

Hip2 -2.650 -2.400

Raver2 -2.650 -2.100

Selk -2.650 -2.490

Tebp -2.660 -2.760

Dhx15 -2.670 -2.570

Amigo -2.680 -2.730

Cyb561d1 -2.690 -2.050

Mapbpip -2.690 -2.840

5033405K12Rik -2.710 -2.620

Rab18 -2.710 -2.400

BC019977 -2.720 -2.000

Galt -2.720 -1.950

Zfp97 -2.720 -2.890

Kctd3 -2.730 -2.150

Tpp2 -2.730 -1.770

4632404H12Rik -2.740 -1.910

Psme4 -2.760 -1.790

6330548O06Rik -2.765 -2.550

Fkbp14 -2.770 -2.430

Vhlh -2.780 -3.260

Papd1 -2.790 -2.100

Pkig -2.790 -3.030

Pgm2l1 -2.800 -2.640

Mgat3 -2.820 -1.710

Rhob -2.840 -2.010

Hnrpa1 -2.850 -4.030

Slitrk3 -2.850 -2.240

Pldn -2.870 -2.130

Ndfip2 -2.880 -2.220

Trappc2 -2.890 -2.190

Evl -2.920 -1.770

Pmpcb -2.920 -2.930

Vapa -2.930 -2.370

Magoh -2.940 -2.250

Brsk2 -2.950 -2.950

Ss18 -2.950 -2.310

Nol11 -2.970 -2.260

Tial1 -2.970 -2.120

Snx3 -3.000 -3.370

Cnksr2 -3.010 -2.130

D030028O16Rik -3.010 -1.890

Kpna3 -3.010 -2.590

Psmd11 -3.020 -2.050

Clcn3 -3.050 -3.380

Id2 -3.050 -3.420

2210412D01Rik -3.090 -2.970

Ube1c -3.090 -2.620

Nefm -3.130 -4.230

Sfrs16 -3.130 -2.600

Ciz1 -3.140 -2.960

Fkbp2 -3.160 -2.440

Ddx3x -3.180 -2.950

Vil2 -3.180 -3.320

Actr3 -3.190 -2.460

Catnd2 -3.210 -2.590

Gpm6a -3.230 -3.680

4933403G14Rik -3.240 -1.840

Gng2 -3.240 -4.100

Sdhd -3.240 -2.230

Atp5a1 -3.260 -2.390

3110050N22Rik -3.290 -2.510

Kpnb1 -3.290 -3.070

Hspcb -3.300 -2.000

Nrxn3 -3.300 -2.740

Golga2 -3.300 -4.400

A230072I16Rik -3.310 -2.960

Pdhb -3.355 -2.765

Herc3 -3.360 -2.840

Tardbp -3.360 -2.090

Adcy9 -3.370 -2.730

Nsbp1 -3.370 -2.560

Ube2j1 -3.430 -2.080

Hars2 -3.440 -3.110

BC038286 -3.450 -3.560

Glt8d1 -3.460 -2.850

Mdh1 -3.470 -2.190

Ndufb2 -3.480 -3.030

1200013F24Rik -3.490 -3.430

Arntl -3.500 -4.570

Ureb1 -3.520 -2.300

Ccl25 -3.530 -3.060

Gucy1b3 -3.540 -3.090

Tsn -3.590 -2.350

Atp6v1a1 -3.600 -2.670

Bxdc1 -3.600 -2.660

Adcy1 -3.630 -3.740

Cyfip2 -3.660 -2.990

Epha7 -3.660 -4.460

Bicd2 -3.670 -2.510

Txnl2 -3.700 -3.390

2810417M05Rik -3.730 -3.300

1300003K24Rik -3.780 -2.570

Ndufb9 -3.800 -3.660

Ghitm -3.810 -2.110

Ndrg4 -3.810 -2.910

Ptpra -3.820 -2.430

Pcmt1 -3.890 -2.960

D10Ertd610e -3.910 -3.410

Hspe1 -3.960 -2.060

1810029G24Rik -3.980 -3.040

Vbp1 -3.980 -2.740

AW125753 -4.010 -3.870

Ssbp1 -4.030 -3.750

Scn1a -4.050 -3.660

Tug1 -4.050 -3.570

Fut8 -4.060 -2.545

Rtn4 -4.100 -4.030

Sgtb -4.240 -3.550

Bok -4.250 -4.070

6430702L12 -4.420 -2.500

Cugbp2 -4.420 -3.340

Neurod2 -4.490 -3.790

Map1lc3b -4.570 -3.020

Sel1l -5.000 -4.570

**Exclusive Genes observed in each treatment**

| **mTBI vs. Sham** |  |  | **mTBI/PHEN vs. Sham** | |  | **PHEN vs. Sham** |  |
| --- | --- | --- | --- | --- | --- | --- | --- |
|  |  |  |  |  |  |  |  |
| **Up regulated** |  |  | **Up regulated** |  |  | **Up regulated** |  |
| **Symbol** | **Z-ratio** |  | **Symbol** | **Z-ratio** |  | **Symbol** | **Z-ratio** |
| Sult1a1 | 7.740 |  | 0610041G09Rik | 6.190 |  | Col6a1 | 7.900 |
| Tnfrsf25 | 4.640 |  | Aebp1 | 5.740 |  | Arv1 | 6.140 |
| LOC545007 | 4.600 |  | Acta2 | 5.390 |  | Znhit3 | 5.020 |
| Xbp1 | 4.250 |  | 0710001E13Rik | 5.140 |  | Adamts2 | 3.890 |
| Gjb6 | 3.860 |  | Igf2 | 5.130 |  | Pnrc2 | 3.750 |
| 2610318I01Rik | 3.710 |  | 9130006A14Rik | 5.010 |  | Rims2 | 3.630 |
| E330018D03Rik | 3.600 |  | Slc13a4 | 4.590 |  | Fzd2 | 3.600 |
| Fbxo30 | 3.570 |  | Apod | 4.470 |  | Appbp2 | 3.230 |
| Htra1 | 3.550 |  | Col23a1 | 4.270 |  | Alox12b | 3.090 |
| Slc15a2 | 3.290 |  | Apaf1 | 4.180 |  | Dhx35 | 2.960 |
| 2310008M10Rik | 3.270 |  | Ctgf | 4.120 |  | Bcl9l | 2.700 |
| 1600019D15Rik | 3.200 |  | Eif4el3 | 3.760 |  | Rnpep | 2.600 |
| P4ha1 | 3.150 |  | Cldn11 | 3.690 |  | 1110035L05Rik | 2.570 |
| Spcs1 | 3.140 |  | Ckb | 3.680 |  | Bcl2l2 | 2.570 |
| Stk36 | 3.140 |  | Gig1 | 3.650 |  | Cbfa2t3h | 2.550 |
| Chordc1 | 3.120 |  | Hap1 | 3.620 |  | Zfp187 | 2.500 |
| Rps2 | 2.990 |  | Slc6a13 | 3.560 |  | Sap30 | 2.420 |
| Trpc2 | 2.970 |  | B230312I18Rik | 3.550 |  | Slc2a3 | 2.410 |
| Eef1d | 2.920 |  | Gm129 | 3.485 |  | Otx1 | 2.400 |
| Mertk | 2.840 |  | Fxyd6 | 3.420 |  | 9130213B05Rik | 2.390 |
| Tmc7 | 2.830 |  | Rims3 | 3.300 |  | Nin | 2.390 |
| Zfp119 | 2.820 |  | Ppap2b | 3.280 |  | Kif1a | 2.370 |
| Nxt2 | 2.810 |  | Zfp537 | 3.270 |  | Zfyve26 | 2.330 |
| Clp1 | 2.780 |  | Igfbp5 | 3.250 |  | Ctnnbl1 | 2.320 |
| Klhl7 | 2.780 |  | Camk2n1 | 3.220 |  | Mknk2 | 2.290 |
| Tsc22d3 | 2.780 |  | Psap | 3.200 |  | Hmgn3 | 2.280 |
| Mrps12 | 2.770 |  | Lamp1 | 3.140 |  | Ptprd | 2.280 |
| Fkbp4 | 2.680 |  | Grit | 3.100 |  | Cecr6 | 2.210 |
| Cox5a | 2.670 |  | Myh11 | 3.030 |  | 1810055G02Rik | 2.200 |
| Ivns1abp | 2.630 |  | Ednrb | 3.000 |  | Pelo | 2.150 |
| B830021E24Rik | 2.550 |  | Slc1a3 | 2.990 |  | Wnt5a | 2.150 |
| EG633640 | 2.550 |  | Ifit3 | 2.940 |  | Atp5c1 | 2.140 |
| Emp1 | 2.500 |  | Rpl23 | 2.930 |  | Slc35f4 | 2.130 |
| Itpr1 | 2.490 |  | Atp5b | 2.920 |  | Dpysl5 | 2.120 |
| Pigc | 2.450 |  | BC011468 | 2.900 |  | Dlx1 | 2.030 |
| Scrg1 | 2.450 |  | Rps17 | 2.860 |  | Man2b1 | 2.030 |
| Skp1a | 2.450 |  | 2610007K22Rik | 2.840 |  | Topors | 2.030 |
| Mapre2 | 2.430 |  | B230104P22Rik | 2.830 |  | Riok3 | 2.020 |
| Irs2 | 2.410 |  | Eef1a1 | 2.820 |  | Fbf1 | 1.980 |
| 4632417K02 | 2.400 |  | B430104H02Rik | 2.800 |  | Vegfb | 1.980 |
| Anxa3 | 2.400 |  | Ahnak | 2.780 |  | Itpkc | 1.970 |
| Irf2 | 2.390 |  | Ubb | 2.780 |  | Sec61a2 | 1.950 |
| Cul3 | 2.330 |  | Myo9b | 2.740 |  | BC031353 | 1.920 |
| 4930438O05Rik | 2.290 |  | Olfm2 | 2.740 |  | Snapc4 | 1.890 |
| D630048P19Rik | 2.280 |  | Gm644 | 2.730 |  | 9430015G10Rik | 1.870 |
| Matr3 | 2.280 |  | E430034L04Rik | 2.660 |  | Abcg4 | 1.870 |
| 1300006C19Rik | 2.260 |  | Wnt4 | 2.660 |  | 2610039E05Rik | 1.860 |
| Uhrf2 | 2.260 |  | Ptn | 2.630 |  | AI847670 | 1.850 |
| 5832424M12 | 2.250 |  | Rpl38 | 2.620 |  | BC046331 | 1.840 |
| BC017133 | 2.230 |  | Dab2 | 2.610 |  | Ccrk | 1.840 |
| Fcgr3 | 2.230 |  | Tuba1b | 2.580 |  | Hdac7a | 1.830 |
| Trim9 | 2.215 |  | Tro | 2.520 |  | Orc5l | 1.830 |
| Bcl2 | 2.210 |  | Cops8 | 2.510 |  | Prkr | 1.820 |
| 2510005D08Rik | 2.200 |  | Tspan7 | 2.510 |  | Zfp87 | 1.820 |
| Fbxo9 | 2.200 |  | Vtn | 2.510 |  | Accn2 | 1.810 |
| 1100001I22Rik | 2.190 |  | Wbp2 | 2.510 |  | Aldh3a2 | 1.810 |
| Aprin | 2.180 |  | 2310022B05Rik | 2.480 |  | AW549877 | 1.810 |
| Bhlhb9 | 2.180 |  | C2 | 2.460 |  | D4Ertd429e | 1.810 |
| Cai | 2.180 |  | Cyba | 2.460 |  | Riok1 | 1.810 |
| Snap23 | 2.150 |  | Atp5g3 | 2.440 |  | 1190005F20Rik | 1.800 |
| Nr2f1 | 2.140 |  | Reln | 2.440 |  | 2410022L05Rik | 1.800 |
| 2210407G14Rik | 2.110 |  | Cox6a1 | 2.430 |  | 2610304F09Rik | 1.800 |
| D19Ertd144e | 2.110 |  | Ndufs6 | 2.430 |  | 3100004P22Rik | 1.800 |
| Gmds | 2.110 |  | Tubb4 | 2.420 |  | 2310067B10Rik | 1.770 |
| Chchd1 | 2.100 |  | Cnp1 | 2.415 |  | Ccnd3 | 1.770 |
| Nipbl | 2.080 |  | Ece1 | 2.400 |  | Keo4 | 1.770 |
| Chuk | 2.020 |  | D7Ertd156e | 2.380 |  | Psme1 | 1.770 |
| 0610039N19Rik | 2.000 |  | Fth1 | 2.370 |  | 4632428M11Rik | 1.760 |
| Srrm2 | 1.960 |  | Rpl41 | 2.370 |  | Acd | 1.750 |
| Clock | 1.950 |  | 0910001L09Rik | 2.360 |  | Stk35 | 1.750 |
| Zfp597 | 1.950 |  | Wnt7a | 2.360 |  | Cacnb3 | 1.740 |
| AU021838 | 1.940 |  | Wbscr1 | 2.320 |  | Cog6 | 1.740 |
| Ccdc56 | 1.940 |  | Card10 | 2.310 |  | Rabl2a | 1.740 |
| Jmjd1a | 1.940 |  | 9030612K14Rik | 2.290 |  | 0610034P02Rik | 1.720 |
| Marcks | 1.940 |  | Gtf2ird2 | 2.290 |  | Osbpl9 | 1.720 |
| Ccng1 | 1.930 |  | Stx1a | 2.280 |  | Nadk | 1.710 |
| 1600002K03Rik | 1.920 |  | LOC218963 | 2.270 |  | Tnfrsf21 | 1.700 |
| Sec24b | 1.910 |  | Atp6ap1 | 2.260 |  | Add3 | 1.690 |
| D12Ertd553e | 1.900 |  | Atp6v1e1 | 2.260 |  | Pik3r3 | 1.690 |
| Gtpbp2 | 1.900 |  | Hebp1 | 2.250 |  | Vkorc1l1 | 1.685 |
| Prkar1a | 1.890 |  | Rab6ip1 | 2.250 |  | Nope | 1.680 |
| Spry3 | 1.890 |  | Atp1a1 | 2.230 |  | 2810437L13Rik | 1.670 |
| Hsf1 | 1.880 |  | C130036G08 | 2.230 |  | 6430517E21Rik | 1.660 |
| Pank4 | 1.880 |  | Tubb5 | 2.220 |  | Arih2 | 1.660 |
| Zfp207 | 1.880 |  | D2Bwg0891e | 2.200 |  | Ase1 | 1.660 |
| AU020772 | 1.860 |  | Ssh3 | 2.200 |  | Rufy1 | 1.660 |
| Sumo1 | 1.850 |  | Vps25 | 2.200 |  | Lrba | 1.650 |
| 2610208M17Rik | 1.840 |  | Atp5j2 | 2.180 |  | Pigk | 1.650 |
| Cops4 | 1.840 |  | Fgd2 | 2.180 |  | Smg6 | 1.650 |
| Hrb | 1.840 |  | Prnp | 2.180 |  | Bet1l | 1.640 |
| Gclm | 1.830 |  | 0610006I08Rik | 2.170 |  | Cox10 | 1.640 |
| Rpl39 | 1.820 |  | Lamb2 | 2.170 |  | Sez6 | 1.640 |
| Pik4cb | 1.800 |  | Rab3d | 2.170 |  | 1200009O22Rik | 1.630 |
| Cct6a | 1.770 |  | Acvr2b | 2.160 |  | 3110023B02Rik | 1.630 |
| 5430428G01Rik | 1.760 |  | Rps19 | 2.160 |  | 4732496O19Rik | 1.620 |
| Gpt2 | 1.760 |  | Eno2 | 2.150 |  | Rbms2 | 1.620 |
| Pycrl | 1.760 |  | Fhl1 | 2.150 |  | Slc1a4 | 1.620 |
| Slc12a6 | 1.750 |  | Ndufa2 | 2.150 |  | A830007L07Rik | 1.610 |
| Ddit3 | 1.740 |  | Slc25a10 | 2.150 |  | Bat1a | 1.610 |
| Zfp276 | 1.740 |  | Mapk1 | 2.140 |  | BC002199 | 1.610 |
| Cd97 | 1.720 |  | Psmd4 | 2.140 |  | Sav1 | 1.610 |
| Gca | 1.720 |  | 3110004L20Rik | 2.130 |  | 2510006D16Rik | 1.600 |
| Lrrc8a | 1.720 |  | Cd9 | 2.130 |  | Crat | 1.600 |
| Fbxl10 | 1.700 |  | Fmo1 | 2.130 |  | Timm8b | 1.600 |
| Ulk1 | 1.700 |  | Mylk | 2.130 |  | Zfp239 | 1.600 |
| Cops3 | 1.680 |  | Ppp2ca | 2.130 |  | Catnbip1 | 1.590 |
| Wbp1 | 1.680 |  | 2310020H20Rik | 2.120 |  | 2610029D06Rik | 1.580 |
| Hspa9 | 1.670 |  | Bag1 | 2.120 |  | Fiz1 | 1.580 |
| Ormdl3 | 1.660 |  | Pea15 | 2.120 |  | Pbx1 | 1.580 |
| Slitrk5 | 1.660 |  | Ppia | 2.120 |  | Rps6ka5 | 1.580 |
| Agps | 1.650 |  | Ppp2r5d | 2.120 |  | Timm10 | 1.580 |
| Cds1 | 1.650 |  | Atp5g1 | 2.090 |  | Eral1 | 1.570 |
| 0610039D01Rik | 1.630 |  | Slc7a5 | 2.090 |  | Jtb | 1.570 |
| Mrps18a | 1.630 |  | Tagln3 | 2.090 |  | Pias1 | 1.570 |
| Hat1 | 1.620 |  | Tiam1 | 2.090 |  | Anks3 | 1.560 |
| 6330403K07Rik | 1.600 |  | Ube1x | 2.080 |  | Fbxo21 | 1.560 |
| Ipp | 1.600 |  | Btf3 | 2.070 |  | AI846148 | 1.550 |
| 2410006F12Rik | 1.590 |  | Gapd | 2.065 |  | Prdm4 | 1.550 |
| Trove2 | 1.590 |  | Rps12 | 2.050 |  | Top3b | 1.550 |
| 1700048E23Rik | 1.580 |  | Tprkb | 2.050 |  | Snag1 | 1.540 |
| Myt1l | 1.580 |  | Fzr1 | 2.040 |  | Adipor1 | 1.530 |
| Rbm5 | 1.580 |  | Pitpnm3 | 2.040 |  | Brp17 | 1.530 |
| Slc11a2 | 1.580 |  | Epb4.1l3 | 2.030 |  | Dfy | 1.520 |
| Srpk1 | 1.580 |  | Prpf19 | 2.030 |  | Exosc10 | 1.510 |
| Impact | 1.570 |  | Rpn2 | 2.030 |  | Ldhd | 1.510 |
| Slc39a3 | 1.570 |  | Mtch1 | 2.020 |  | Ppm1a | 1.510 |
| Rnf14 | 1.560 |  | Cdk2ap1 | 2.010 |  | Lypla3 | 1.500 |
| Tsc2 | 1.560 |  | D8Ertd325e | 2.010 |  | Optn | 1.010 |
| Zfp295 | 1.560 |  | Hgs | 2.010 |  | Cyhr1 | 0.860 |
| 5830427H10Rik | 1.550 |  | Tob2 | 2.010 |  | 5133400G04Rik | 0.567 |
| 1110020M19Rik | 1.540 |  | Aco2 | 2.000 |  | Xab1 | 0.515 |
| Zfyve20 | 1.540 |  | Uaca | 2.000 |  | Eif2b1 | 0.295 |
| Polr2j | 1.530 |  | 2410104I19Rik | 1.995 |  |  |  |
| 1700021K19Rik | 1.520 |  | Gm1821 | 1.990 |  | **Exclusive Genes** |  |
| Gtpbp4 | 1.520 |  | Rps26 | 1.990 |  | **Down regulated** | **Z-ratio** |
| Zw10 | 1.520 |  | Ahi1 | 1.980 |  | **Symbol** |  |
| Bbs4 | 1.510 |  | Nrarp | 1.980 |  | Lancl1 | -0.110 |
| Nudt5 | 1.475 |  | Cdipt | 1.970 |  | Vps35 | -1.510 |
| Irf3 | 1.405 |  | Dctn2 | 1.970 |  | 2210021J22Rik | -1.540 |
| Hnrpm | 1.275 |  | Dnchc1 | 1.970 |  | Usp2 | -1.540 |
| Ars2 | 1.245 |  | Gnb4 | 1.970 |  | Prmt6 | -1.550 |
| Nupl1 | 1.125 |  | Ndufc1 | 1.970 |  | Ing4 | -1.590 |
| Zhx1 | 0.985 |  | Scarf2 | 1.970 |  | Arhgap12 | -1.600 |
| Rnf11 | 0.795 |  | Thy1 | 1.960 |  | LOC622404 | -1.610 |
| Otub2 | 0.750 |  | Wbp5 | 1.960 |  | 4833424P18Rik | -1.620 |
| Elavl2 | 0.430 |  | Prosapip1 | 1.950 |  | Mrps2 | -1.620 |
| Rhobtb2 | 0.340 |  | Ly6h | 1.940 |  | Gatad1 | -1.640 |
| Hnrpdl | 0.305 |  | Hdac11 | 1.940 |  | Sirt5 | -1.640 |
| **Exclusive Genes**  **Down regulated** | **Z-ratio** |  | Nedd8 | 1.940 |  | Mreg | -1.650 |
| **Symbol**  3110035E14Rik | -0.315 |  | Snca | 1.940 |  | Siat8c | -1.670 |
| Tmem66 | -1.320 |  | Akr7a5 | 1.930 |  | 2400001E08Rik | -1.680 |
| Eif4g1 | -1.500 |  | Cox8a | 1.930 |  | Atp6v0a1 | -1.680 |
| Smpd2 | -1.500 |  | Mrpl28 | 1.930 |  | Dbi | -1.680 |
| Elp3 | -1.510 | | Arl6ip1 | 1.920 |  | Dpp3 | -1.690 |
| Tbl3 | -1.510 |  | Ednra | 1.920 |  | Htatip2 | -1.690 |
| U2af2 | -1.510 |  | Flot1 | 1.920 |  | Slc20a2 | -1.690 |
| Vps53 | -1.510 |  | 2310036O22Rik | 1.910 |  | Zfp219 | -1.690 |
| 2610205E22Rik | -1.530 |  | Hnrpl | 1.910 |  | Lyrm2 | -1.710 |
| Plec1 | -1.540 |  | Ssbp4 | 1.910 |  | BC011248 | -1.720 |
| Zfp608 | -1.540 |  | Tssc1 | 1.910 |  | Pacsin2 | -1.750 |
| Rbm9 | -1.550 |  | B230374F23Rik | 1.900 |  | Ehbp1 | -1.760 |
| 5730494M16Rik | -1.560 |  | Ddah1 | 1.900 |  | Mageh1 | -1.760 |
| Commd10 | -1.560 |  | Gng13 | 1.900 |  | Ndrg3 | -1.770 |
| H2afx | -1.560 |  | E130307J07Rik | 1.890 |  | Freq | -1.780 |
| Faf1 | -1.570 |  | Dscr1l2 | 1.880 |  | LOC242681 | -1.780 |
| Mapkap1 | -1.570 |  | Rce1 | 1.880 |  | Prnpip1 | -1.780 |
| Mpst | -1.570 |  | Renbp | 1.870 |  | 4921507I02Rik | -1.790 |
| S100a1 | -1.580 |  | Rnaset2 | 1.870 |  | Atf4 | -1.790 |
| Smarcb1 | -1.580 |  | Srebf1 | 1.870 |  | D10Ertd438e | -1.790 |
| AI646023 | -1.590 |  | Ifitm2 | 1.860 |  | Smad3 | -1.790 |
| Pep4 | -1.590 |  | Parl | 1.860 |  | Tmem108 | -1.800 |
| 2010004B12Rik | -1.600 |  | Sfxn1 | 1.860 |  | Tmem8 | -1.800 |
| Usp30 | -1.600 |  | Sh3gl2 | 1.860 |  | Ctsz | -1.810 |
| Chd4 | -1.610 |  | Txndc5 | 1.860 |  | 2010011I20Rik | -1.820 |
| Fxc1 | -1.610 |  | Ywhah | 1.860 |  | Lix1 | -1.820 |
| Med25 | -1.620 |  | Napg | 1.860 |  | D0H8S2298E | -1.840 |
| Acat2 | -1.630 |  | Hmgcs1 | 1.840 |  | Tbrg4 | -1.850 |
| Palm | -1.630 |  | Ntsr2 | 1.840 |  | Golga5 | -1.870 |
| Rcl1 | -1.650 |  | Rsad1 | 1.840 |  | Ptpn1 | -1.880 |
| 1810054O13Rik | -1.680 |  | Ccs | 1.830 |  | 5133401N09Rik | -1.890 |
| Apba2 | -1.680 |  | Pkm2 | 1.830 |  | Tuba4 | -1.890 |
| Cs | -1.680 |  | Syn1 | 1.830 |  | Foxred1 | -1.900 |
| Ube1l | -1.680 |  | Blcap | 1.820 |  | Usp3 | -1.980 |
| Apbb3 | -1.690 |  | Coq10b | 1.820 |  | Slc39a7 | -1.990 |
| Znhit2 | -1.700 |  | 2500003M10Rik | 1.810 |  | Vamp1 | -1.990 |
| Mina | -1.710 |  | Atox1 | 1.810 |  | Ahsa1 | -2.000 |
| Ss18l1 | -1.710 |  | Capzb | 1.810 |  | Rufy3 | -2.010 |
| Tmem17 | -1.710 |  | Exosc7 | 1.810 |  | Ndufs4 | -2.030 |
| Leprel2 | -1.720 |  | 1810027O10Rik | 1.800 |  | Arf6 | -2.050 |
| 3930401K13Rik | -1.730 |  | Lass5 | 1.800 |  | Rab11a | -2.050 |
| Ppp5c | -1.730 |  | Plxna2 | 1.800 |  | Atp6v1g2 | -2.060 |
| Rdbp | -1.730 |  | Rassf2 | 1.800 |  | 5730437N04Rik | -2.090 |
| Siva | -1.730 |  | Ube2d2 | 1.800 |  | Slc11a1 | -2.090 |
| Eftud1 | -1.740 |  | Cspg5 | 1.790 |  | Ush2a | -2.090 |
| Rhbdd2 | -1.740 |  | Pfkm | 1.780 |  | Cstf1 | -2.100 |
| 8430427H17Rik | -1.750 |  | Rpl23a | 1.780 |  | 6720485C15Rik | -2.120 |
| 1700030E05Rik | -1.770 |  | Tbc1d2b | 1.780 |  | Plekhg5 | -2.130 |
| Txnrd2 | -1.775 |  | Wdr45l | 1.770 |  | Sbf1 | -2.150 |
| Ift81 | -1.780 |  | 2310003H01Rik | 1.760 |  | Npdc1 | -2.160 |
| LOC381633 | -1.780 |  | Bax | 1.760 |  | Thoc6 | -2.160 |
| Nrbp | -1.780 |  | BC003885 | 1.760 |  | Ddx3y | -2.180 |
| Smo | -1.780 |  | Chd5 | 1.760 |  | Calca | -2.200 |
| 2310050B20Rik | -1.790 |  | Dpysl2 | 1.760 |  | Rps5 | -2.200 |
| Csf1r | -1.790 |  | Fscn1 | 1.760 |  | Dap3 | -2.205 |
| Cops7b | -1.800 |  | Hn1 | 1.760 |  | Foxk1 | -2.210 |
| 2700094L05Rik | -1.810 |  | Idh3g | 1.760 |  | Hadh2 | -2.220 |
| Thoc3 | -1.820 |  | Necap1 | 1.760 |  | Flna | -2.260 |
| B230217C12Rik | -1.830 |  | Park7 | 1.760 |  | Anp32a | -2.270 |
| Cope | -1.830 |  | C230093N12Rik | 1.750 |  | C630007B19Rik | -2.280 |
| Gne | -1.830 |  | Gm2a | 1.750 |  | Idb1 | -2.280 |
| 2310016C08Rik | -1.850 |  | Llgl1 | 1.750 |  | Tmem2 | -2.290 |
| Dnajc19 | -1.850 |  | Ube2l3 | 1.750 |  | Senp5 | -2.300 |
| OTTMUSG00000000421 | -1.850 |  | Vps28 | 1.750 |  | Snx6 | -2.340 |
| Mboat5 | -1.860 |  | Gstz1 | 1.740 |  | Fbxo34 | -2.345 |
| Sema6b | -1.860 |  | 1110061N23Rik | 1.730 |  | Gprk6 | -2.380 |
| Slc16a6 | -1.860 |  | Eno1 | 1.730 |  | Dtna | -2.385 |
| Snapap | -1.860 |  | Npm3-ps1 | 1.730 |  | Ntrk3 | -2.450 |
| Dullard | -1.870 |  | Ppm1b | 1.730 |  | 2210013K02Rik | -2.490 |
| 1810037G04Rik | -1.880 |  | Scara3 | 1.730 |  | 1810021J13Rik | -2.510 |
| 1810038H16Rik | -1.880 |  | Bex2 | 1.725 |  | Tef | -2.550 |
| Cox15 | -1.880 |  | 2810012G03Rik | 1.720 |  | 2410001C21Rik | -2.570 |
| Dab1 | -1.880 |  | Cd81 | 1.720 |  | Itpk1 | -2.570 |
| Relb | -1.880 |  | Dync1i2 | 1.720 |  | C1qa | -2.610 |
| Bai3 | -1.890 |  | P4hb | 1.720 |  | Gpd1 | -2.720 |
| D1Bwg0212e | -1.890 |  | Rtn1 | 1.720 |  | Rhoj | -2.720 |
| Brf1 | -1.900 |  | 1810031K02Rik | 1.710 |  | Ptpre | -2.780 |
| LOC380625 | -1.910 |  | Galk1 | 1.710 |  | Spag1 | -2.810 |
| Uxt | -1.910 |  | C78212 | 1.700 |  | Eef2 | -2.955 |
| Etv3 | -1.920 |  | Rpl31 | 1.700 |  | Rpl35a | -3.060 |
| Rps6ka1 | -1.920 |  | Tceal5 | 1.700 |  | Luzp1 | -3.150 |
| AK129375 | -1.930 |  | 1700027J05Rik | 1.690 |  | Rab1b | -3.180 |
| Muted | -1.965 |  | 2510048O06Rik | 1.680 |  | Rps25 | -3.210 |
| Scand1 | -1.970 |  | BC035295 | 1.680 |  | Zfp238 | -3.220 |
| Keap1 | -1.975 |  | Cyb5r3 | 1.680 |  | Dgkz | -3.230 |
| 3110040N11Rik | -1.980 |  | Sbds | 1.680 |  | Rasgrp1 | -3.280 |
| Obfc1 | -2.000 |  | Snx17 | 1.680 |  | Tubb2b | -3.300 |
| Vps16 | -2.000 |  | 2310047D13Rik | 1.670 |  | Sfrs5 | -3.460 |
| Snx4 | -2.010 |  | Ndufa3 | 1.670 |  | Add1 | -3.580 |
| Vdrip | -2.010 |  | Plod3 | 1.670 |  | Centg1 | -3.650 |
| 1190002C06Rik | -2.020 |  | Spec1 | 1.670 |  | 2610103J23Rik | -3.670 |
| Cln6 | -2.020 |  | Cbx7 | 1.660 |  | Scd2 | -3.860 |
| Kif22 | -2.020 |  | Uqcrh | 1.660 |  | Insig1 | -3.940 |
| Pms2 | -2.020 |  | Atp6v1h | 1.650 |  | 2610204M08Rik | -4.190 |
| Tst | -2.030 |  | Nav1 | 1.650 |  | 1700021C14Rik | -4.590 |
| D8Ertd354e | -2.040 |  | Scamp1 | 1.650 |  | Sri | -4.640 |
| Pgls | -2.040 |  | Akap12 | 1.640 |  | Hdc | -5.090 |
| Sf4 | -2.050 |  | Hdgf | 1.640 |  | Manbal | -7.420 |
| E330036I19Rik | -2.060 |  | Nsf | 1.640 |  | Rapgef5 | -10.770 |
| Rps6ka4 | -2.060 |  | Wdr34 | 1.640 |  |  |  |
| Wipi1 | -2.060 |  | Arf1 | 1.630 |  |  |  |
| 1110032E23Rik | -2.070 |  | Arl2 | 1.630 |  |  |  |
| Srd5a2l | -2.075 |  | BC018601 | 1.630 |  |  |  |
| Dtr | -2.080 |  | Hdgfrp2 | 1.630 |  |  |  |
| AI606181 | -2.090 |  | Mrpl38 | 1.630 |  |  |  |
| Grm1 | -2.100 |  | Zfp213 | 1.630 |  |  |  |
| Chmp7 | -2.130 |  | Bscl2 | 1.620 |  |  |  |
| S100a16 | -2.130 |  | Gnaz | 1.620 |  |  |  |
| Tceb3bp1 | -2.135 |  | LOC240906 | 1.620 |  |  |  |
| 9330132O05Rik | -2.140 |  | Fnta | 1.610 |  |  |  |
| Idb3 | -2.150 |  | 6720456B07Rik | 1.600 |  |  |  |
| Cyp4v3 | -2.160 |  | 9130023D20Rik | 1.600 |  |  |  |
| Lims2 | -2.165 |  | Eps15-rs | 1.600 |  |  |  |
| Mvd | -2.170 |  | Mrpl23 | 1.600 |  |  |  |
| Lin7b | -2.180 |  | Nicn1 | 1.600 |  |  |  |
| D130029J02Rik | -2.200 |  | Rpl28 | 1.600 |  |  |  |
| Ly86 | -2.210 |  | Unc84b | 1.600 |  |  |  |
| Mgat1 | -2.210 |  | Wdr13 | 1.600 |  |  |  |
| 6030411F23Rik | -2.220 |  | Psmb2 | 1.595 |  |  |  |
| Bdh | -2.220 |  | 2310047M10Rik | 1.590 |  |  |  |
| Mrps28 | -2.220 |  | Ergic3 | 1.590 |  |  |  |
| Hip1r | -2.230 |  | Fbxo2 | 1.590 |  |  |  |
| Lhfpl2 | -2.230 |  | 2410012C07Rik | 1.580 |  |  |  |
| Pigl | -2.230 |  | Atp6v0d1 | 1.580 |  |  |  |
| Slc8a2 | -2.240 |  | Btg1 | 1.580 |  |  |  |
| 1700084C01Rik | -2.280 |  | Eif3s4 | 1.580 |  |  |  |
| AJ430384 | -2.290 |  | Klf2 | 1.580 |  |  |  |
| Slc25a12 | -2.290 |  | Rnf10 | 1.580 |  |  |  |
| B930041F14Rik | -2.310 |  | 2900011O08Rik | 1.570 |  |  |  |
| Abcc8 | -2.320 |  | Got2 | 1.570 |  |  |  |
| Ddr1 | -2.320 |  | Rab11b | 1.570 |  |  |  |
| Abi3 | -2.360 |  | Zfp275 | 1.570 |  |  |  |
| Acta1 | -2.360 |  | 2310016M24Rik | 1.560 |  |  |  |
| Dyrk1b | -2.360 |  | 2610507L03Rik | 1.560 |  |  |  |
| Ltap | -2.370 |  | Ap3m2 | 1.560 |  |  |  |
| Slc38a3 | -2.390 |  | Cerk | 1.560 |  |  |  |
| Fbxo32 | -2.410 |  | D11Bwg0280e | 1.560 |  |  |  |
| Prr7 | -2.410 |  | Ptpru | 1.560 |  |  |  |
| R74862 | -2.410 |  | Psmb4 | 1.550 |  |  |  |
| Fastk | -2.430 |  | Zfp282 | 1.550 |  |  |  |
| Sat2 | -2.430 |  | Ckmt1 | 1.540 |  |  |  |
| Mpp3 | -2.440 |  | Fcho1 | 1.540 |  |  |  |
| 6330406I15Rik | -2.450 |  | Gars | 1.540 |  |  |  |
| Sema5b | -2.450 |  | Nme2 | 1.540 |  |  |  |
| Dicer1 | -2.500 |  | Tbca | 1.540 |  |  |  |
| Akap1 | -2.515 |  | 1700093E07Rik | 1.530 |  |  |  |
| Pltp | -2.570 |  | AW742319 | 1.530 |  |  |  |
| Alg2 | -2.580 |  | Cops6 | 1.530 |  |  |  |
| Tubgcp2 | -2.660 |  | Hrmt1l2 | 1.530 |  |  |  |
| A830039B04Rik | -2.670 |  | Lrrc49 | 1.530 |  |  |  |
| Acbd4 | -2.670 |  | Sirt2 | 1.530 |  |  |  |
| Clmn | -2.680 |  | Ank | 1.520 |  |  |  |
| Crmp1 | -2.710 |  | Pcbp3 | 1.520 |  |  |  |
| Msr2 | -2.710 |  | Timm44 | 1.520 |  |  |  |
| Zfp46 | -2.720 |  | Tmem9b | 1.520 |  |  |  |
| Akp2 | -2.740 |  | Ttll1 | 1.520 |  |  |  |
| Lmna | -2.903 |  | 1110018G07Rik | 1.510 |  |  |  |
| Hsd3b2 | -2.910 |  | 1200014J11Rik | 1.510 |  |  |  |
| Rnf126 | -2.920 |  | C130052I12Rik | 1.510 |  |  |  |
| Ick | -2.930 |  | Mapkapk2 | 1.510 |  |  |  |
| Sprn | -2.940 |  | Nap1l4 | 1.510 |  |  |  |
| 4930429A22Rik | -2.990 |  | Nisch | 1.510 |  |  |  |
| F730014I05Rik | -3.000 |  | Ocil | 1.510 |  |  |  |
| 2010005A06Rik | -3.050 |  | Pitpn | 1.510 |  |  |  |
| P2ry13 | -3.080 |  | Ypel3 | 1.510 |  |  |  |
| Abcb10 | -3.100 |  | 4930504E06Rik | 1.500 |  |  |  |
| Islr2 | -3.230 |  | Oxct1 | 1.500 |  |  |  |
| Jund1 | -3.250 |  | BC046404 | 1.275 |  |  |  |
| Lst1 | -3.330 |  | Fmnl3 | 1.245 |  |  |  |
| 4631426J05Rik | -3.380 |  | Pld3 | 1.090 |  |  |  |
| Arfgap1 | -3.420 |  | Abcb8 | 0.877 |  |  |  |
| Pgpep1 | -3.860 |  | Tpi1 | 0.740 |  |  |  |
| Dutp | -4.010 |  | Cd151 | 0.700 |  |  |  |
| Hspb6 | -4.170 |  | Hspa8 | 0.480 |  |  |  |
| Arrdc4 | -4.330 |  | Ubc | 0.385 |  |  |  |
| S100a6 | -4.330 |  | Rps3 | 0.305 |  |  |  |
|  |  |  | Slc25a11 | 0.155 |  |  |  |
|  |  |  | Rasgrf1 | 0.130 |  |  |  |
|  |  |  | Arpc1b | 0.075 |  |  |  |
|  |  |  | 5730410E15Rik | 0.040 |  |  |  |
|  |  |  | Syt11 | 0.010 |  |  |  |
|  |  |  | **Exclusive Genes**  **Down regulated**  **Symbol**  Mapt | **Z-ratio**  -0.270 |  |  |  |
|  |  |  | Tcf25 | -0.345 |  |  |  |
|  |  |  | Psmc5 | -1.500 |  |  |  |
|  |  |  | AI413414 | -1.510 |  |  |  |
|  |  |  | Cog3 | -1.510 | | |  |
|  |  |  | D15Wsu169e | -1.510 |  |  |  |
|  |  |  | Lrp1 | -1.510 |  |  |  |
|  |  |  | Pygo1 | -1.510 |  |  |  |
|  |  |  | A630054L15Rik | -1.520 |  |  |  |
|  |  |  | Acate3 | -1.520 |  |  |  |
|  |  |  | Atpaf2 | -1.520 |  |  |  |
|  |  |  | Atxn7l2 | -1.520 |  |  |  |
|  |  |  | B3galt5 | -1.520 |  |  |  |
|  |  |  | B430218L07Rik | -1.520 |  |  |  |
|  |  |  | Dedd | -1.520 |  |  |  |
|  |  |  | Polr2g | -1.520 |  |  |  |
|  |  |  | Pom121 | -1.520 |  |  |  |
|  |  |  | Smek2 | -1.520 |  |  |  |
|  |  |  | Adra2c | -1.530 |  |  |  |
|  |  |  | Arhgap1 | -1.530 |  |  |  |
|  |  |  | DXImx46e | -1.530 |  |  |  |
|  |  |  | Thap7 | -1.530 |  |  |  |
|  |  |  | 2510001I10Rik | -1.540 |  |  |  |
|  |  |  | 2810427I04Rik | -1.540 |  |  |  |
|  |  |  | Dnaja4 | -1.540 |  |  |  |
|  |  |  | Gm631 | -1.540 |  |  |  |
|  |  |  | Pdcl | -1.540 |  |  |  |
|  |  |  | Tex10 | -1.540 |  |  |  |
|  |  |  | Acp2 | -1.550 |  |  |  |
|  |  |  | Chchd5 | -1.550 |  |  |  |
|  |  |  | Khdrbs2 | -1.550 |  |  |  |
|  |  |  | Lonrf2 | -1.550 |  |  |  |
|  |  |  | Nnp1 | -1.550 |  |  |  |
|  |  |  | Tmem19 | -1.550 |  |  |  |
|  |  |  | Ttc14 | -1.550 |  |  |  |
|  |  |  | Txndc1 | -1.550 |  |  |  |
|  |  |  | 1500005A01Rik | -1.560 |  |  |  |
|  |  |  | Bcas3 | -1.560 |  |  |  |
|  |  |  | Dcx | -1.560 |  |  |  |
|  |  |  | Ddx46 | -1.560 |  |  |  |
|  |  |  | Fcmd | -1.560 |  |  |  |
|  |  |  | Rbms3 | -1.560 |  |  |  |
|  |  |  | 3110001H15Rik | -1.570 |  |  |  |
|  |  |  | Cml1 | -1.570 |  |  |  |
|  |  |  | Gpr177 | -1.570 |  |  |  |
|  |  |  | Acvr1 | -1.580 |  |  |  |
|  |  |  | Fh1 | -1.580 |  |  |  |
|  |  |  | Narg2 | -1.580 |  |  |  |
|  |  |  | Pmm1 | -1.580 |  |  |  |
|  |  |  | Snap29 | -1.580 |  |  |  |
|  |  |  | 1110057K04Rik | -1.590 |  |  |  |
|  |  |  | Odf2 | -1.590 |  |  |  |
|  |  |  | Pparbp | -1.590 |  |  |  |
|  |  |  | Vdac2 | -1.590 |  |  |  |
|  |  |  | 0610009B22Rik | -1.600 |  |  |  |
|  |  |  | AI854635 | -1.600 |  |  |  |
|  |  |  | Dlat | -1.600 |  |  |  |
|  |  |  | Kifap3 | -1.600 |  |  |  |
|  |  |  | Cbr1 | -1.610 |  |  |  |
|  |  |  | Sec14l1 | -1.610 |  |  |  |
|  |  |  | Trp53bp1 | -1.610 |  |  |  |
|  |  |  | Zcchc3 | -1.610 |  |  |  |
|  |  |  | Zfp131 | -1.610 |  |  |  |
|  |  |  | Zfp748 | -1.610 |  |  |  |
|  |  |  | 2610002M06Rik | -1.620 |  |  |  |
|  |  |  | Ak2 | -1.620 |  |  |  |
|  |  |  | B020018G12Rik | -1.620 |  |  |  |
|  |  |  | Gabrg2 | -1.620 |  |  |  |
|  |  |  | Gmpr2 | -1.620 |  |  |  |
|  |  |  | Rpl4 | -1.620 |  |  |  |
|  |  |  | 2010305A19Rik | -1.630 |  |  |  |
|  |  |  | Csnk2a2 | -1.630 |  |  |  |
|  |  |  | Jundm2 | -1.630 |  |  |  |
|  |  |  | Rpp38 | -1.630 |  |  |  |
|  |  |  | Rtn4ip1 | -1.630 |  |  |  |
|  |  |  | Sfxn4 | -1.630 |  |  |  |
|  |  |  | 2410127E16Rik | -1.640 |  |  |  |
|  |  |  | 4932442K08Rik | -1.640 |  |  |  |
|  |  |  | Ache | -1.640 |  |  |  |
|  |  |  | Amigo1 | -1.640 |  |  |  |
|  |  |  | Cstf3 | -1.640 |  |  |  |
|  |  |  | Lamp2 | -1.640 |  |  |  |
|  |  |  | Mmp16 | -1.640 |  |  |  |
|  |  |  | Ptprb | -1.640 |  |  |  |
|  |  |  | Tjp2 | -1.640 |  |  |  |
|  |  |  | Xrcc4 | -1.640 |  |  |  |
|  |  |  | 1810020D17Rik | -1.650 |  |  |  |
|  |  |  | 2810439F02Rik | -1.650 |  |  |  |
|  |  |  | Abtb1 | -1.650 |  |  |  |
|  |  |  | BC065078 | -1.650 |  |  |  |
|  |  |  | Bre | -1.650 |  |  |  |
|  |  |  | Kcns2 | -1.650 |  |  |  |
|  |  |  | Lrrn3 | -1.650 |  |  |  |
|  |  |  | Mark3 | -1.650 |  |  |  |
|  |  |  | Polr3h | -1.650 |  |  |  |
|  |  |  | Stk40 | -1.650 |  |  |  |
|  |  |  | Tcfe3 | -1.650 |  |  |  |
|  |  |  | Dapk1 | -1.655 |  |  |  |
|  |  |  | 2410002O22Rik | -1.660 |  |  |  |
|  |  |  | Jph4 | -1.660 |  |  |  |
|  |  |  | Sepm | -1.660 |  |  |  |
|  |  |  | Zfp451 | -1.660 |  |  |  |
|  |  |  | 3830406C13Rik | -1.670 |  |  |  |
|  |  |  | 6430706D22Rik | -1.670 |  |  |  |
|  |  |  | Ankrd10 | -1.670 |  |  |  |
|  |  |  | Ephb6 | -1.670 |  |  |  |
|  |  |  | Mtmr1 | -1.670 |  |  |  |
|  |  |  | Nenf | -1.670 |  |  |  |
|  |  |  | Nit1 | -1.670 |  |  |  |
|  |  |  | Wdr5 | -1.670 |  |  |  |
|  |  |  | Fbxo33 | -1.680 |  |  |  |
|  |  |  | LOC382010 | -1.680 |  |  |  |
|  |  |  | Map2k4 | -1.680 |  |  |  |
|  |  |  | 1810014F10Rik | -1.690 |  |  |  |
|  |  |  | 1810015C04Rik | -1.690 |  |  |  |
|  |  |  | 2310073E15Rik | -1.690 |  |  |  |
|  |  |  | AW049604 | -1.690 |  |  |  |
|  |  |  | Cdkl2 | -1.690 |  |  |  |
|  |  |  | Manea | -1.690 |  |  |  |
|  |  |  | Robo1 | -1.690 |  |  |  |
|  |  |  | Tfpt | -1.690 |  |  |  |
|  |  |  | 1700060H10Rik | -1.700 |  |  |  |
|  |  |  | AA536749 | -1.700 |  |  |  |
|  |  |  | Cyb5d2 | -1.700 |  |  |  |
|  |  |  | Tdrd3 | -1.700 |  |  |  |
|  |  |  | Tnfrsf12a | -1.700 |  |  |  |
|  |  |  | Trak1 | -1.700 |  |  |  |
|  |  |  | 1700023M03Rik | -1.710 |  |  |  |
|  |  |  | Acsbg1 | -1.710 |  |  |  |
|  |  |  | Dnmt1 | -1.710 |  |  |  |
|  |  |  | Trim11 | -1.710 |  |  |  |
|  |  |  | Appbp1 | -1.720 |  |  |  |
|  |  |  | Pex3 | -1.720 |  |  |  |
|  |  |  | Pitpnm1 | -1.720 |  |  |  |
|  |  |  | Syn2 | -1.720 |  |  |  |
|  |  |  | Znrd1 | -1.720 |  |  |  |
|  |  |  | 1700025K23Rik | -1.730 |  |  |  |
|  |  |  | Numbl | -1.730 |  |  |  |
|  |  |  | Ppm1e | -1.730 |  |  |  |
|  |  |  | Tceal3 | -1.730 |  |  |  |
|  |  |  | Ttc15 | -1.730 |  |  |  |
|  |  |  | D6Ertd365e | -1.740 |  |  |  |
|  |  |  | Dgkh | -1.740 |  |  |  |
|  |  |  | Ywhab | -1.745 |  |  |  |
|  |  |  | 5730469M10Rik | -1.750 |  |  |  |
|  |  |  | 6332401O19Rik | -1.750 |  |  |  |
|  |  |  | Arpc5l | -1.750 |  |  |  |
|  |  |  | D430039N05Rik | -1.750 |  |  |  |
|  |  |  | Gbf1 | -1.750 |  |  |  |
|  |  |  | Lsm8 | -1.750 |  |  |  |
|  |  |  | Npy5r | -1.750 |  |  |  |
|  |  |  | A930009M04Rik | -1.760 |  |  |  |
|  |  |  | AI413782 | -1.760 |  |  |  |
|  |  |  | Dnclic1 | -1.760 |  |  |  |
|  |  |  | Mesdc1 | -1.760 |  |  |  |
|  |  |  | Plch2 | -1.760 |  |  |  |
|  |  |  | Sh2bp1 | -1.760 |  |  |  |
|  |  |  | Srp9 | -1.760 |  |  |  |
|  |  |  | Tmem4 | -1.760 |  |  |  |
|  |  |  | Dlgap3 | -1.770 |  |  |  |
|  |  |  | LOC384214 | -1.770 |  |  |  |
|  |  |  | Lrrfip1 | -1.770 |  |  |  |
|  |  |  | Nudt10 | -1.770 |  |  |  |
|  |  |  | Pfn2 | -1.770 |  |  |  |
|  |  |  | Tcte1l | -1.770 |  |  |  |
|  |  |  | Ube2a | -1.770 |  |  |  |
|  |  |  | 1500041N16Rik | -1.780 |  |  |  |
|  |  |  | Gosr1 | -1.780 |  |  |  |
|  |  |  | Lars | -1.780 |  |  |  |
|  |  |  |  |  |  |  |  |
|  |  |  | Eed | -1.790 |  |  |  |
|  |  |  | Fndc3b | -1.790 |  |  |  |
|  |  |  | Ythdf3 | -1.790 |  |  |  |
|  |  |  | Arsa | -1.800 |  |  |  |
|  |  |  | Hist1h2an | -1.800 |  |  |  |
|  |  |  | Orc3l | -1.800 |  |  |  |
|  |  |  | Polr3k | -1.800 |  |  |  |
|  |  |  | Stag1 | -1.800 |  |  |  |
|  |  |  | A230106M15Rik | -1.810 |  |  |  |
|  |  |  | B930062P21Rik | -1.810 |  |  |  |
|  |  |  | Rgs10 | -1.810 |  |  |  |
|  |  |  | Rnf25 | -1.810 |  |  |  |
|  |  |  | Tspan31 | -1.810 |  |  |  |
|  |  |  | Wdr37 | -1.810 |  |  |  |
|  |  |  | Nlk | -1.820 |  |  |  |
|  |  |  | Tbrg1 | -1.820 |  |  |  |
|  |  |  | Sec63 | -1.825 |  |  |  |
|  |  |  | 2310005P05Rik | -1.830 |  |  |  |
|  |  |  | 2600013N14Rik | -1.830 |  |  |  |
|  |  |  | BC027061 | -1.830 |  |  |  |
|  |  |  | D430025H09Rik | -1.830 |  |  |  |
|  |  |  | M6pr | -1.830 |  |  |  |
|  |  |  | 1110056N09Rik | -1.840 |  |  |  |
|  |  |  | Klf3 | -1.840 |  |  |  |
|  |  |  | Tceb1 | -1.840 |  |  |  |
|  |  |  | 2310057G13Rik | -1.845 |  |  |  |
|  |  |  | Cox18 | -1.850 |  |  |  |
|  |  |  | Fbxo18 | -1.850 |  |  |  |
|  |  |  | Pik4ca | -1.850 |  |  |  |
|  |  |  | Ppargc1a | -1.850 |  |  |  |
|  |  |  | Tmem32 | -1.850 |  |  |  |
|  |  |  | 2210417J20Rik | -1.860 |  |  |  |
|  |  |  | 6430601A21Rik | -1.860 |  |  |  |
|  |  |  | Cmpk | -1.860 |  |  |  |
|  |  |  | Coil | -1.860 |  |  |  |
|  |  |  | LOC234374 | -1.860 |  |  |  |
|  |  |  | Slc30a6 | -1.860 |  |  |  |
|  |  |  | 1110067D22Rik | -1.870 |  |  |  |
|  |  |  | 5330410G16Rik | -1.870 |  |  |  |
|  |  |  | Atf7ip | -1.870 |  |  |  |
|  |  |  | Pip3ap | -1.870 |  |  |  |
|  |  |  | 1300007B12Rik | -1.880 |  |  |  |
|  |  |  | Hps4 | -1.880 |  |  |  |
|  |  |  | Lpl | -1.880 |  |  |  |
|  |  |  | Tax1bp1 | -1.880 |  |  |  |
|  |  |  | 6330509G02Rik | -1.890 |  |  |  |
|  |  |  | Kctd4 | -1.890 |  |  |  |
|  |  |  | 2810422B04Rik | -1.900 |  |  |  |
|  |  |  | Eif3s10 | -1.900 |  |  |  |
|  |  |  | Fbxl4 | -1.900 |  |  |  |
|  |  |  | Nt5c2 | -1.900 |  |  |  |
|  |  |  | Tgfbr2 | -1.900 |  |  |  |
|  |  |  | 2310057D15Rik | -1.910 |  |  |  |
|  |  |  | Zfp263 | -1.910 |  |  |  |
|  |  |  | 6330409N04Rik | -1.920 |  |  |  |
|  |  |  | Armc8 | -1.920 |  |  |  |
|  |  |  | Yme1l1 | -1.920 |  |  |  |
|  |  |  | Nck2 | -1.930 |  |  |  |
|  |  |  | 2900026H06Rik | -1.940 |  |  |  |
|  |  |  | Abi1 | -1.940 |  |  |  |
|  |  |  | Hira | -1.940 |  |  |  |
|  |  |  | Stk4 | -1.940 |  |  |  |
|  |  |  | 2210404D11Rik | -1.950 |  |  |  |
|  |  |  | 1110013L07Rik | -1.960 |  |  |  |
|  |  |  | Cyp2d22 | -1.960 |  |  |  |
|  |  |  | Ddhd1 | -1.960 |  |  |  |
|  |  |  | Ina | -1.960 |  |  |  |
|  |  |  | Nek9 | -1.960 |  |  |  |
|  |  |  | Thop1 | -1.960 |  |  |  |
|  |  |  | 4833439L19Rik | -1.970 |  |  |  |
|  |  |  | D11Wsu47e | -1.970 |  |  |  |
|  |  |  | Dexi | -1.970 |  |  |  |
|  |  |  | Gm83 | -1.970 |  |  |  |
|  |  |  | Phtf2 | -1.970 |  |  |  |
|  |  |  | Rab1 | -1.970 |  |  |  |
|  |  |  | Rbbp7 | -1.970 |  |  |  |
|  |  |  | Tra1 | -1.970 |  |  |  |
|  |  |  | Ccndbp1 | -1.980 |  |  |  |
|  |  |  | Dusp6 | -1.980 |  |  |  |
|  |  |  | Grik4 | -1.980 |  |  |  |
|  |  |  | Itm2b | -1.980 |  |  |  |
|  |  |  | Osbpl6 | -1.980 |  |  |  |
|  |  |  | B930008K04Rik | -1.990 |  |  |  |
|  |  |  | Foxj3 | -1.990 |  |  |  |
|  |  |  | Gbif | -1.990 |  |  |  |
|  |  |  | Pde8a | -1.990 |  |  |  |
|  |  |  | 1700065O13Rik | -2.000 |  |  |  |
|  |  |  | 1810047C23Rik | -2.000 |  |  |  |
|  |  |  | Elac2 | -2.000 |  |  |  |
|  |  |  | Nap1l5 | -2.000 |  |  |  |
|  |  |  | Sorcs3 | -2.000 |  |  |  |
|  |  |  | Surf1 | -2.000 |  |  |  |
|  |  |  | Khdrbs3 | -2.010 |  |  |  |
|  |  |  | Pcyox1 | -2.010 |  |  |  |
|  |  |  | Pdk3 | -2.010 |  |  |  |
|  |  |  | 1110019C08Rik | -2.020 |  |  |  |
|  |  |  | Apex1 | -2.020 |  |  |  |
|  |  |  | Dhx9 | -2.020 |  |  |  |
|  |  |  | Spock1 | -2.020 |  |  |  |
|  |  |  | Uchl5 | -2.030 |  |  |  |
|  |  |  | Psip1 | -2.040 |  |  |  |
|  |  |  | rp9 | -2.040 |  |  |  |
|  |  |  | 2610031L17Rik | -2.050 |  |  |  |
|  |  |  | Brdt | -2.050 |  |  |  |
|  |  |  | Ctnnb1 | -2.050 |  |  |  |
|  |  |  | Pdgfa | -2.050 |  |  |  |
|  |  |  | Sc5d | -2.050 |  |  |  |
|  |  |  | 2810055E05Rik | -2.060 |  |  |  |
|  |  |  | Lmo3 | -2.060 |  |  |  |
|  |  |  | Rap2c | -2.060 |  |  |  |
|  |  |  | Terf2ip | -2.060 |  |  |  |
|  |  |  | Eef1e1 | -2.070 |  |  |  |
|  |  |  | Skiv2l | -2.070 |  |  |  |
|  |  |  | 5330414D10Rik | -2.080 |  |  |  |
|  |  |  | Rb1 | -2.080 |  |  |  |
|  |  |  | Sdhb | -2.080 |  |  |  |
|  |  |  | Smad1 | -2.080 |  |  |  |
|  |  |  | 2010204I15Rik | -2.090 |  |  |  |
|  |  |  | 2310056P07Rik | -2.090 |  |  |  |
|  |  |  | Scrn1 | -2.090 |  |  |  |
|  |  |  | Axot | -2.100 |  |  |  |
|  |  |  | Kpna1 | -2.100 |  |  |  |
|  |  |  | LOC433182 | -2.100 |  |  |  |
|  |  |  | 1810003N24Rik | -2.110 |  |  |  |
|  |  |  | 4121402D02Rik | -2.110 |  |  |  |
|  |  |  | Nhlrc1 | -2.110 |  |  |  |
|  |  |  | Zfp692 | -2.110 |  |  |  |
|  |  |  | 1810074P20Rik | -2.120 |  |  |  |
|  |  |  | 2410018M08Rik | -2.120 |  |  |  |
|  |  |  | Ap3b1 | -2.120 |  |  |  |
|  |  |  | Exoc7 | -2.120 |  |  |  |
|  |  |  | Igsf4b | -2.120 |  |  |  |
|  |  |  | Plk2 | -2.120 |  |  |  |
|  |  |  | A930008A22Rik | -2.130 |  |  |  |
|  |  |  | C030011O14Rik | -2.130 |  |  |  |
|  |  |  | Cul1 | -2.130 |  |  |  |
|  |  |  | Large | -2.130 |  |  |  |
|  |  |  | Rchy1 | -2.130 |  |  |  |
|  |  |  | Spg4 | -2.130 |  |  |  |
|  |  |  | Baiap2 | -2.135 |  |  |  |
|  |  |  | 0610009I22Rik | -2.140 |  |  |  |
|  |  |  | Cdkn2d | -2.140 |  |  |  |
|  |  |  | Nsmaf | -2.140 |  |  |  |
|  |  |  | Tram1 | -2.140 |  |  |  |
|  |  |  | Kbtbd7 | -2.150 |  |  |  |
|  |  |  | 4933407N01Rik | -2.160 |  |  |  |
|  |  |  | BC016235 | -2.160 |  |  |  |
|  |  |  | Rerg | -2.160 |  |  |  |
|  |  |  | Ring1 | -2.160 |  |  |  |
|  |  |  | Tfam | -2.160 |  |  |  |
|  |  |  | Lman1 | -2.170 |  |  |  |
|  |  |  | Mrps35 | -2.170 |  |  |  |
|  |  |  | Rgma | -2.170 |  |  |  |
|  |  |  | Zfyve27 | -2.170 |  |  |  |
|  |  |  | Abat | -2.180 |  |  |  |
|  |  |  | Atp5l | -2.180 |  |  |  |
|  |  |  | Eps15 | -2.180 |  |  |  |
|  |  |  | Evc2 | -2.180 |  |  |  |
|  |  |  | Nat5 | -2.180 |  |  |  |
|  |  |  | Trhde | -2.190 |  |  |  |
|  |  |  | BC055107 | -2.200 |  |  |  |
|  |  |  | Zap70 | -2.200 |  |  |  |
|  |  |  | Abcb9 | -2.205 |  |  |  |
|  |  |  | 2610312B22Rik | -2.210 |  |  |  |
|  |  |  | Akr1b3 | -2.210 |  |  |  |
|  |  |  | Nell2 | -2.210 |  |  |  |
|  |  |  | Rga | -2.210 |  |  |  |
|  |  |  | Ibtk | -2.220 |  |  |  |
|  |  |  | Uchl3 | -2.220 |  |  |  |
|  |  |  | 9430023B20Rik | -2.230 |  |  |  |
|  |  |  | Jmjd2c | -2.230 |  |  |  |
|  |  |  | Npy2r | -2.230 |  |  |  |
|  |  |  | Rem2 | -2.230 |  |  |  |
|  |  |  | BC027088 | -2.240 |  |  |  |
|  |  |  | C80879 | -2.240 |  |  |  |
|  |  |  | Mrps21 | -2.240 |  |  |  |
|  |  |  | Nab1 | -2.240 |  |  |  |
|  |  |  | Zfp75 | -2.240 |  |  |  |
|  |  |  | D12Ertd771e | -2.250 |  |  |  |
|  |  |  | Faim | -2.255 |  |  |  |
|  |  |  | 2610022K04Rik | -2.260 |  |  |  |
|  |  |  | Aifm1 | -2.260 |  |  |  |
|  |  |  | Ensa | -2.260 |  |  |  |
|  |  |  | Gzf1 | -2.260 |  |  |  |
|  |  |  | Kctd6 | -2.280 |  |  |  |
|  |  |  | Mapk10 | -2.280 |  |  |  |
|  |  |  | Dnaja2 | -2.290 |  |  |  |
|  |  |  | Nr1d2 | -2.290 |  |  |  |
|  |  |  | Tceal1 | -2.290 |  |  |  |
|  |  |  | Ubtd2 | -2.290 |  |  |  |
|  |  |  | Cox6c | -2.300 |  |  |  |
|  |  |  | Tmem136 | -2.300 |  |  |  |
|  |  |  | Cpsf2 | -2.310 |  |  |  |
|  |  |  | 0610007P22Rik | -2.315 |  |  |  |
|  |  |  | Rtn4rl1 | -2.320 |  |  |  |
|  |  |  | Scyl1 | -2.320 |  |  |  |
|  |  |  | Vps24 | -2.320 |  |  |  |
|  |  |  | 6530418L21Rik | -2.320 |  |  |  |
|  |  |  | 1600012H06Rik | -2.325 |  |  |  |
|  |  |  | Pla2g7 | -2.330 |  |  |  |
|  |  |  | Rad23b | -2.330 |  |  |  |
|  |  |  | AI593442 | -2.335 |  |  |  |
|  |  |  | 2510012J08Rik | -2.340 |  |  |  |
|  |  |  | Cacnb2 | -2.340 |  |  |  |
|  |  |  | C6.1A | -2.350 |  |  |  |
|  |  |  | Insig2 | -2.350 |  |  |  |
|  |  |  | Schip1 | -2.350 |  |  |  |
|  |  |  | Zmynd11 | -2.350 |  |  |  |
|  |  |  | Als2cr13 | -2.360 |  |  |  |
|  |  |  | Copeb | -2.360 |  |  |  |
|  |  |  | 5330439J01Rik | -2.365 |  |  |  |
|  |  |  | 4122402O22Rik | -2.370 |  |  |  |
|  |  |  | 4933426M11Rik | -2.370 |  |  |  |
|  |  |  | 8430415E04Rik | -2.370 |  |  |  |
|  |  |  | Pafah1b1 | -2.375 |  |  |  |
|  |  |  | Rrn3 | -2.380 |  |  |  |
|  |  |  | Narg1 | -2.395 |  |  |  |
|  |  |  | Lrpprc | -2.400 |  |  |  |
|  |  |  | Slc25a17 | -2.400 |  |  |  |
|  |  |  | Fgf10 | -2.410 |  |  |  |
|  |  |  | Snap91 | -2.410 |  |  |  |
|  |  |  | Rnf6 | -2.420 |  |  |  |
|  |  |  | Cdk5rap1 | -2.430 |  |  |  |
|  |  |  | Cox7a2 | -2.430 |  |  |  |
|  |  |  | Btbd9 | -2.440 |  |  |  |
|  |  |  | Kif3a | -2.440 |  |  |  |
|  |  |  | Msrb2 | -2.440 |  |  |  |
|  |  |  | Prps2 | -2.450 |  |  |  |
|  |  |  | Gkap1 | -2.470 |  |  |  |
|  |  |  | Lip1 | -2.480 |  |  |  |
|  |  |  | Rad52 | -2.480 |  |  |  |
|  |  |  | Slc26a4 | -2.480 |  |  |  |
|  |  |  | Smap1 | -2.480 |  |  |  |
|  |  |  | 4930572J05Rik | -2.490 |  |  |  |
|  |  |  | Usp1 | -2.510 |  |  |  |
|  |  |  | 2610205H19Rik | -2.520 |  |  |  |
|  |  |  | AW212394 | -2.520 |  |  |  |
|  |  |  | 4933434E20Rik | -2.530 |  |  |  |
|  |  |  | Ascc3l1 | -2.530 |  |  |  |
|  |  |  | Fnip1 | -2.540 |  |  |  |
|  |  |  | Gpr22 | -2.540 |  |  |  |
|  |  |  | Rras2 | -2.540 |  |  |  |
|  |  |  | Rbbp9 | -2.550 |  |  |  |
|  |  |  | Arrdc3 | -2.560 |  |  |  |
|  |  |  | Chl1 | -2.560 |  |  |  |
|  |  |  | Tcfcp2l2 | -2.560 |  |  |  |
|  |  |  | Psmd14 | -2.570 |  |  |  |
|  |  |  | 5830415L20Rik | -2.580 |  |  |  |
|  |  |  | Iqgap2 | -2.580 |  |  |  |
|  |  |  | Slc8a1 | -2.580 |  |  |  |
|  |  |  | Bzw1 | -2.590 |  |  |  |
|  |  |  | Epha5 | -2.590 |  |  |  |
|  |  |  | Osbpl8 | -2.590 |  |  |  |
|  |  |  | Thsd7b | -2.590 |  |  |  |
|  |  |  | Prdx5 | -2.595 |  |  |  |
|  |  |  | Elavl4 | -2.600 |  |  |  |
|  |  |  | Flrt3 | -2.600 |  |  |  |
|  |  |  | Lgi1 | -2.600 |  |  |  |
|  |  |  | Chchd7 | -2.610 |  |  |  |
|  |  |  | Gosr2 | -2.620 |  |  |  |
|  |  |  | Rab9 | -2.620 |  |  |  |
|  |  |  | Dhx33 | -2.630 |  |  |  |
|  |  |  | Drd1a | -2.630 |  |  |  |
|  |  |  | Hsd17b12 | -2.630 |  |  |  |
|  |  |  | 4932409F11Rik | -2.640 |  |  |  |
|  |  |  | Pa2g4 | -2.640 |  |  |  |
|  |  |  | Ulk2 | -2.640 |  |  |  |
|  |  |  | Cpne7 | -2.650 |  |  |  |
|  |  |  | Dock4 | -2.650 |  |  |  |
|  |  |  | Mbnl2 | -2.650 |  |  |  |
|  |  |  | Cdkal1 | -2.660 |  |  |  |
|  |  |  | Tsg101 | -2.670 |  |  |  |
|  |  |  | Mrpl52 | -2.680 |  |  |  |
|  |  |  | Lphn1 | -2.690 |  |  |  |
|  |  |  | Kcnab3 | -2.700 |  |  |  |
|  |  |  | Rab5a | -2.710 |  |  |  |
|  |  |  | 2810453I06Rik | -2.720 |  |  |  |
|  |  |  | Akap8 | -2.720 |  |  |  |
|  |  |  | BC051080 | -2.725 |  |  |  |
|  |  |  | 2210402C18Rik | -2.730 |  |  |  |
|  |  |  | Neto1 | -2.745 |  |  |  |
|  |  |  | Chrna4 | -2.770 |  |  |  |
|  |  |  | Hprt | -2.770 |  |  |  |
|  |  |  | Ttl | -2.770 |  |  |  |
|  |  |  | 2810453K03Rik | -2.780 |  |  |  |
|  |  |  | Lcat | -2.780 |  |  |  |
|  |  |  | Cblb | -2.790 |  |  |  |
|  |  |  | Frmpd4 | -2.790 |  |  |  |
|  |  |  | Prox1 | -2.790 |  |  |  |
|  |  |  | 2810036L13Rik | -2.800 |  |  |  |
|  |  |  | Bzw2 | -2.800 |  |  |  |
|  |  |  | Clpx | -2.800 |  |  |  |
|  |  |  | Pdpk1 | -2.810 |  |  |  |
|  |  |  | Hist2h2bb | -2.820 |  |  |  |
|  |  |  | Mylc2b | -2.830 |  |  |  |
|  |  |  | Pcdh20 | -2.830 |  |  |  |
|  |  |  | Commd1 | -2.850 |  |  |  |
|  |  |  | A530082C11Rik | -2.860 |  |  |  |
|  |  |  | Cdh8 | -2.875 |  |  |  |
|  |  |  | Anxa11 | -2.890 |  |  |  |
|  |  |  | Sparcl1 | -2.890 |  |  |  |
|  |  |  | 2900041A09Rik | -2.905 |  |  |  |
|  |  |  | Nol4 | -2.905 |  |  |  |
|  |  |  | Prss35 | -2.910 |  |  |  |
|  |  |  | C630041L24Rik | -2.920 |  |  |  |
|  |  |  | Pgrmc1 | -2.930 |  |  |  |
|  |  |  | Cidea | -2.970 |  |  |  |
|  |  |  | Golph2 | -2.970 |  |  |  |
|  |  |  | Cat | -2.990 |  |  |  |
|  |  |  | Nqo3a2 | -3.000 |  |  |  |
|  |  |  | 1810055E12Rik | -3.010 |  |  |  |
|  |  |  | BC066107 | -3.010 |  |  |  |
|  |  |  | 1810044A24Rik | -3.020 |  |  |  |
|  |  |  | Ndufa5 | -3.020 |  |  |  |
|  |  |  | D330017J20Rik | -3.030 |  |  |  |
|  |  |  | Nhlrc2 | -3.030 |  |  |  |
|  |  |  | Prkcc | -3.030 |  |  |  |
|  |  |  | 2810432L12Rik | -3.060 |  |  |  |
|  |  |  | Rdh14 | -3.060 |  |  |  |
|  |  |  | AB182283 | -3.070 |  |  |  |
|  |  |  | 1810035L17Rik | -3.130 |  |  |  |
|  |  |  | Timm13a | -3.130 |  |  |  |
|  |  |  | Ube2q | -3.140 |  |  |  |
|  |  |  | Pvrl3 | -3.145 |  |  |  |
|  |  |  | Zswim5 | -3.160 |  |  |  |
|  |  |  | 2510003E04Rik | -3.170 |  |  |  |
|  |  |  | Foxg1 | -3.170 |  |  |  |
|  |  |  | Pabpc1 | -3.170 |  |  |  |
|  |  |  | Lrrtm1 | -3.190 |  |  |  |
|  |  |  | Eif3s8 | -3.230 |  |  |  |
|  |  |  | Usp14 | -3.270 |  |  |  |
|  |  |  | Nsg1 | -3.290 |  |  |  |
|  |  |  | Polb | -3.350 |  |  |  |
|  |  |  | Smc5l1 | -3.370 |  |  |  |
|  |  |  | Lhfp | -3.410 |  |  |  |
|  |  |  | Ntrk2 | -3.450 |  |  |  |
|  |  |  | Timm9 | -3.460 |  |  |  |
|  |  |  | Hist1h2bk | -3.500 |  |  |  |
|  |  |  | B230219D22Rik | -3.510 |  |  |  |
|  |  |  | Ercc5 | -3.550 |  |  |  |
|  |  |  | Hist1h2bj | -3.550 |  |  |  |
|  |  |  | Tpd52l1 | -3.600 |  |  |  |
|  |  |  | Trim37 | -3.610 |  |  |  |
|  |  |  | Them2 | -3.640 |  |  |  |
|  |  |  | Uqcrb | -3.670 |  |  |  |
|  |  |  | Adora1 | -3.700 |  |  |  |
|  |  |  | Hist1h2bn | -3.720 |  |  |  |
|  |  |  | Kcnv1 | -3.900 |  |  |  |
|  |  |  | Zfml | -3.940 |  |  |  |
|  |  |  | Hist1h2be | -3.960 |  |  |  |
|  |  |  | C630002B14Rik | -4.020 |  |  |  |
|  |  |  | Dst | -4.070 |  |  |  |
|  |  |  | Hist1h2bc | -4.070 |  |  |  |
|  |  |  | Scoc | -4.250 |  |  |  |
|  |  |  | Poli | -4.400 |  |  |  |
|  |  |  | D130043K22Rik | -4.650 |  |  |  |
|  |  |  | Hist1h2bm | -5.220 |  |  |  |
|  |  |  | Cnnm1 | -6.540 |  |  |  |
|  |  |  | Deadc1 | -6.920 |  |  |  |
|  |  |  | Xlr4a | -6.980 |  |  |  |
|  |  |  |  |  |  |  |  |
|  |  |  |  |  |  |  |  |
|  |  |  |  |  |  |  |  |
