# Supplementary material for: Cognitive Impairments Induced by Concussive Mild Traumatic Brain Injury in Mouse Are Ameliorated by Treatment with Phenserine via Multiple Non-Cholinergic and Cholinergic Mechanisms
Source: PLoS One. 2016 Jun 2;11(6):e0156493. doi: 10.1371/journal.pone.0156493 (PMC4890804; doi:10.1371/journal.pone.0156493)
Supplement: S2 Table — (DOCX) [file pone.0156493.s002.docx]

**Supplemental Table illustrating the identities of significantly regulated gene ontologies indicated in Figure 4B**

**Common Gene Ontologies observed in all three treatments**

**Gene Ontology Term Z-score Z-score Z-score**

**Up regulated mTBI vs. Sham mTBI/PHEN vs. Sham PHEN vs. Sham**

GO0004984 OLFACTORY RECEPTOR ACTIVITY 9.419 11.063 8.499

GO0007608 SENSORY PERCEPTION OF SMELL 9.330 10.836 8.462

GO0004930 G PROTEIN COUPLED RECEPTOR ACTIVITY 8.531 10.318 8.429

GO0007186 G PROTEIN COUPLED RECEPTOR PROTEIN SIG 7.657 10.164 8.194

GO0004871 SIGNAL TRANSDUCER ACTIVITY 7.324 9.998 8.440

GO0004872 RECEPTOR ACTIVITY 7.140 8.892 6.706

GO0007165 SIGNAL TRANSDUCTION 5.482 8.529 6.937

GO0005576 EXTRACELLULAR REGION 3.658 5.555 4.027

GO0016503 PHEROMONE RECEPTOR ACTIVITY 3.461 3.843 3.163

GO0005550 PHEROMONE BINDING 3.418 3.616 2.877

GO0019236 RESPONSE TO PHEROMONE 3.294 3.671 2.982

GO0001584 RHODOPSIN LIKE RECEPTOR ACTIVITY 2.729 3.668 3.541

GO0008188 NEUROPEPTIDE RECEPTOR ACTIVITY 2.317 3.114 3.326

GO0016712 OXIDOREDUCTASE ACTIVITY ACTING ON PAIRE 1.870 2.300 1.757

GO0006091 GENERATION OF PRECURSOR METABOLITES 1.863 1.884 1.788

GO0006952 DEFENSE RESPONSE 1.784 2.728 1.636

GO0001895 RETINAL HOMEOSTASIS 1.760 1.695 2.681

GO0060048 CARDIAC MUSCLE CONTRACTION 1.638 2.090 1.837

GO0015672 MONOVALENT INORGANIC CATION TRANSPORT 1.555 2.819 2.302

GO0015077 MONOVALENT INORGANIC CATION TRANSMEMBRAN 1.521 2.797 2.288

**Gene Ontology Term Z-score Z-score Z-score**

**Down regulated mTBI vs. Sham mTBI/PHEN vs. Sham PHEN vs. Sham**

GO0005245 VOLTAGE GATED CALCIUM CHANNEL ACTIVITY -1.675 -1.717 -1.881

GO0006287 BASE EXCISION REPAIR GAP FILLING -1.749 -2.906 -2.005

GO0001975 RESPONSE TO AMPHETAMINE -1.754 -2.577 -2.831

GO0003724 RNA HELICASE ACTIVITY -1.799 -3.268 -2.454

GO0003960 NADPH QUINONE REDUCTASE ACTIVITY -1.838 -2.536 -1.917

GO0007625 GROOMING BEHAVIOR -1.977 -2.436 -2.438

GO0019452 L CYSTEINE CATABOLIC PROCESS TO TAURINE -1.990 -1.525 -1.633

GO0019530 TAURINE METABOLIC PROCESS -1.990 -1.525 -1.633

GO0004952 DOPAMINE RECEPTOR ACTIVITY -2.118 -3.263 -3.548

GO0005794 GOLGI APPARATUS -2.186 -3.069 -2.960

GO0004970 IONOTROPIC GLUTAMATE RECEPTOR ACTIVITY -2.399 -3.273 -3.027

GO0005234 EXTRACELLULAR GLUTAMATE GATED ION CHANNEL -2.399 -3.273 -3.027

GO0016831 CARBOXY LYASE ACTIVITY -2.481 -2.241 -3.236

GO0005003 EPHRIN RECEPTOR ACTIVITY -2.567 -3.532 -3.658

GO0019904 PROTEIN DOMAIN SPECIFIC BINDING -2.593 -2.665 -2.986

GO0006996 ORGANELLE ORGANIZATION AND BIOGENESIS -2.613 -2.258 -1.957

GO0005099 RAS GTPASE ACTIVATOR ACTIVITY -2.664 -2.702 -2.037

GO0031103 AXON REGENERATION -2.739 -3.122 -2.874

GO0007212 DOPAMINE RECEPTOR SIGNALING PATHWAY -2.978 -3.722 -4.473

GO0015031 PROTEIN TRANSPORT -3.034 -4.998 -2.974

GO0005634 NUCLEUS -3.056 -5.018 -3.400

GO0017119 GOLGI TRANSPORT COMPLEX -3.737 -2.586 -2.812

**Gene Ontologies observed in pairwise comparisons**

**Gene Ontology Term Z-score Z-score**

**Up regulated mTBI vs. Sham mTBI/PHEN vs. Sham**

GO0005843 CYTOSOLIC SMALL RIBOSOMAL SUBUNIT 6.330 6.077

GO0005840 RIBOSOME 4.803 3.065

GO0003735 STRUCTURAL CONSTITUENT OF RIBOSOME 4.482 2.947

GO0005830 CYTOSOLIC RIBOSOME (SENSU EUKARYOTA) 4.111 3.450

GO0019843 RRNA BINDING 3.909 3.561

GO0015986 ATP SYNTHESIS COUPLED PROTON TRANSPORT 3.168 4.214

GO0005615 EXTRACELLULAR SPACE 2.983 4.587

GO0004295 TRYPSIN ACTIVITY 2.902 2.051

GO0005842 CYTOSOLIC LARGE RIBOSOMAL SUBUNIT 2.867 2.187

GO0008366 AXON ENSHEATHMENT 2.832 3.412

GO0006508 PROTEOLYSIS 2.770 4.065

GO0004252 SERINE TYPE ENDOPEPTIDASE ACTIVITY 2.756 2.253

GO0016469 PROTON TRANSPORTING TWO SECTOR ATPASE 2.720 4.290

GO0006825 COPPER ION TRANSPORT 2.661 2.251

GO0046933 HYDROGEN ION TRANSPORTING ATP SYNTHASE 2.592 4.158

GO0005625 SOLUBLE FRACTION 2.575 2.471

GO0046961 HYDROGEN ION TRANSPORTING ATPASE ACTIVITY 2.547 4.363

GO0007566 EMBRYO IMPLANTATION 2.473 2.013

GO0043565 SEQUENCE SPECIFIC DNA BINDING 2.448 2.334

GO0008250 OLIGOSACCHARYL TRANSFERASE COMPLEX 2.329 2.174

GO0005604 BASEMENT MEMBRANE 2.318 3.457

GO0008233 PEPTIDASE ACTIVITY 2.294 1.930

GO0050896 RESPONSE TO STIMULUS 2.241 2.247

GO0005792 MICROSOME 2.168 2.520

GO0005375 COPPER ION TRANSMEMBRANE TRANSPORTER 2.168 1.762

GO0015078 HYDROGEN ION TRANSMEMBRANE TRANSPORTER 2.117 3.636

GO0030659 CYTOPLASMIC VESICLE MEMBRANE 2.031 1.760

GO0005581 COLLAGEN 1.941 3.587

GO0042589 ZYMOGEN GRANULE MEMBRANE 1.894 2.189

GO0006892 POST GOLGI VESICLE MEDIATED TRANSPORT 1.813 2.180

GO0051726 REGULATION OF CELL CYCLE 1.744 2.160

GO0004499 FLAVIN CONTAINING MONOOXYGENASE ACTIVITY 1.707 1.908

GO0031227 INTRINSIC TO ENDOPLASMIC RETICULUM MEMBR 1.707 1.908

GO0001504 NEUROTRANSMITTER UPTAKE 1.672 2.993

GO0050381 UNSPECIFIC MONOOXYGENASE ACTIVITY 1.655 1.771

GO0030020 EXTRACELLULAR MATRIX STRUCTURAL CONSTITU 1.653 3.772

**Gene Ontology Term Z-score Z-score**

**Down regulated mTBI vs. Sham mTBI/PHEN vs. Sham**

GO0019201 NUCLEOTIDE KINASE ACTIVITY -1.529 -1.668

GO0021953 CENTRAL NERVOUS SYSTEM NEURON DIFFERENTION -1.572 -1.555

GO0001601 PEPTIDE YY RECEPTOR ACTIVITY -1.576 -3.253

GO0008168 METHYLTRANSFERASE ACTIVITY -1.642 -2.406

GO0017056 STRUCTURAL CONSTITUENT OF NUCLEAR PORE -1.723 -1.779

GO0007431 SALIVARY GLAND DEVELOPMENT -1.819 -1.642

GO0050508 GLUCURONOSYL N ACETYLGLUCOSAMINYL PROTEO -1.869 -1.595

GO0004649 POLY(ADP RIBOSE) GLYCOHYDROLASE ACTIVITY -1.915 -1.753

GO0005643 NUCLEAR PORE -2.016 -3.589

GO0006487 PROTEIN AMINO ACID N LINKED GLYCOSYLATIO -2.023 -2.217

GO0005216 ION CHANNEL ACTIVITY -2.114 -2.399

GO0006281 DNA REPAIR -2.136 -2.306

GO0003910 DNA LIGASE (ATP) ACTIVITY -2.351 -1.915

GO0017070 U6 SNRNA BINDING -2.355 -2.620

GO0005730 NUCLEOLUS -2.440 -2.278

GO0006744 UBIQUINONE BIOSYNTHETIC PROCESS -2.898 -3.130

GO0060158 DOPAMINE RECEPTOR PHOSPHOLIPASE C ACTIV -2.913 -3.432

GO0005244 VOLTAGE GATED ION CHANNEL ACTIVITY -2.927 -2.983

GO0006260 DNA REPLICATION -3.226 -2.444

GO0005515 PROTEIN BINDING -3.386 -2.395

**Gene Ontology Term Z-score Z-score**

**Up regulated mTBI/PHEN vs. Sham PHEN vs. Sham**

GO0005578 PROTEINACEOUS EXTRACELLULAR MATRIX 5.064 2.753

GO0016021 INTEGRAL TO MEMBRANE 4.761 2.726

GO0005201 EXTRACELLULAR MATRIX STRUCTURAL CONSTITU 4.743 3.816

GO0007155 CELL ADHESION 3.192 2.365

GO0005887 INTEGRAL TO PLASMA MEMBRANE 3.185 2.491

GO0001503 OSSIFICATION 3.127 3.064

GO0000015 PHOSPHOPYRUVATE HYDRATASE COMPLEX 2.853 2.317

GO0004634 PHOSPHOPYRUVATE HYDRATASE ACTIVITY 2.853 2.317

GO0045595 REGULATION OF CELL DIFFERENTIATION 2.794 2.491

GO0050699 WW DOMAIN BINDING 2.700 2.202

GO0006907 PINOCYTOSIS 2.588 2.391

GO0006911 PHAGOCYTOSIS ENGULFMENT 2.532 1.637

GO0050728 NEGATIVE REGULATION OF INFLAMMATORY RESP 2.099 1.746

GO0007548 SEX DIFFERENTIATION 2.004 2.404

GO0006903 VESICLE TARGETING 1.972 1.942

GO0004994 SOMATOSTATIN RECEPTOR ACTIVITY 1.882 2.321

GO0044237 CELLULAR METABOLIC PROCESS 1.874 2.311

GO0004311 FARNESYLTRANSTRANSFERASE ACTIVITY 1.864 2.280

GO0042157 LIPOPROTEIN METABOLIC PROCESS 1.863 1.605

GO0004536 DEOXYRIBONUCLEASE ACTIVITY 1.766 1.651

GO0007606 SENSORY PERCEPTION OF CHEMICAL STIMULUS 1.651 1.606

GO0004449 ISOCITRATE DEHYDROGENASE (NAD+) ACTIVITY 1.550 1.552

GO0048856 ANATOMICAL STRUCTURE DEVELOPMENT 1.531 1.950

GO0045197 ESTABLISHMENT AND OR MAINTENANCE 1.530 1.672

**Gene Ontology Term Z-score Z-score**

**Down regulated mTBI/PHEN vs. Sham PHEN vs. Sham**

GO0030136 CLATHRIN COATED VESICLE -1.756 -1.547

GO0030178 NEGATIVE REGULATION OF WNT RECEPTOR SIGN -1.859 -2.466

GO0005793 ER GOLGI INTERMEDIATE COMPARTMENT -1.945 -1.959

GO0042799 HISTONE LYSINE N METHYLTRANSFERASE ACTIV -1.954 -1.602

GO0016291 ACYL COA THIOESTERASE ACTIVITY -2.071 -1.796

GO0006829 ZINC ION TRANSPORT -2.168 -2.131

GO0005786 SIGNAL RECOGNITION PARTICLE ENDOPLASMIC -2.214 -2.367

GO0006614 SRP DEPENDENT COTRANSLATIONAL PROTEIN TA -2.273 -2.575

GO0009266 RESPONSE TO TEMPERATURE STIMULUS -2.274 -2.018

GO0006637 ACYL COA METABOLIC PROCESS -2.375 -2.155

GO0031252 LEADING EDGE -2.504 -2.279

GO0016829 LYASE ACTIVITY -2.543 -3.704

GO0009116 NUCLEOSIDE METABOLIC PROCESS -2.567 -1.537

GO0007409 AXONOGENESIS -2.595 -3.153

GO0005737 CYTOPLASM -2.609 -2.484

GO0003743 TRANSLATION INITIATION FACTOR ACTIVITY -2.689 -2.347

GO0048227 PLASMA MEMBRANE TO ENDOSOME TRANSPORT -2.775 -2.544

GO0043087 REGULATION OF GTPASE ACTIVITY -2.804 -1.720

GO0006607 NLS BEARING SUBSTRATE IMPORT INTO NUCLEU -2.867 -2.826

GO0006397 MRNA PROCESSING -3.124 -2.320

GO0051028 MRNA TRANSPORT -3.213 -2.441

GO0008380 RNA SPLICING -3.286 -2.742

GO0006512 UBIQUITIN CYCLE -3.982 -2.016

GO0005739 MITOCHONDRION -4.095 -3.027

GO0000395 NUCLEAR MRNA 5 SPLICE SITE RECOGNITION -4.366 -3.139

GO0016482 CYTOPLASMIC TRANSPORT -4.366 -3.139

GO0048025 NEGATIVE REGULATION OF NUCLEAR MRNA SPLI -4.366 -3.139

GO0050733 RS DOMAIN BINDING -4.366 -3.139

GO0045104 INTERMEDIATE FILAMENT CYTOSKELETON ORGAN -4.615 -6.076

GO0005694 CHROMOSOME -5.117 -3.954

GO0045787 POSITIVE REGULATION OF PROGRESSION THROU -5.361 -4.541

GO0000786 NUCLEOSOME -5.563 -4.266

GO0007001 CHROMOSOME ORGANIZATION AND BIOGENESIS -6.036 -4.767

GO0006334 NUCLEOSOME ASSEMBLY -6.406 -5.711

**Gene Ontology Term Z-score Z-score**

**Up regulated mTBI vs. Sham PHEN vs. Sham**

GO0001786 PHOSPHATIDYLSERINE BINDING 2.287 2.152

GO0031941 FILAMENTOUS ACTIN 2.250 1.853

GO0005149 INTERLEUKIN 1 RECEPTOR BINDING 2.222 1.649

GO0007343 EGG ACTIVATION 2.103 1.654

GO0006665 SPHINGOLIPID METABOLIC PROCESS 1.963 3.509

GO0008290 F ACTIN CAPPING PROTEIN COMPLEX 1.948 1.938

GO0008745 N ACETYLMURAMOYL L ALANINE AMIDASE ACTIV 1.791 1.502

GO0009253 PEPTIDOGLYCAN CATABOLIC PROCESS 1.791 1.502

GO0043193 POSITIVE REGULATION OF GENE SPECIFIC TRA 1.735 1.565

GO0004331 FRUCTOSE 2 6 BISPHOSPHATE 2 PHOSPHATASE 1.717 1.613

GO0006003 FRUCTOSE 2 6 BISPHOSPHATE METABOLIC PROC 1.717 1.613

GO0004879 LIGAND DEPENDENT NUCLEAR RECEPTOR ACTIVI 1.636 2.019

**Gene Ontology Term Z-score Z-score**

**Down regulated mTBI vs. Sham PHEN vs. Sham**

GO0009898 INTERNAL SIDE OF PLASMA MEMBRANE -1.673 -1.731

GO0005337 NUCLEOSIDE TRANSMEMBRANE TRANSPORTER ACT -1.945 -2.057

GO0005975 CARBOHYDRATE METABOLIC PROCESS -1.983 -1.954

GO0007169 TRANSMEMBRANE RECEPTOR PROTEIN TYROSINE -1.989 -2.876

GO0030060 L MALATE DEHYDROGENASE ACTIVITY -2.114 -2.671

GO0003985 ACETYL COA C ACETYLTRANSFERASE ACTIVITY -2.268 -2.414

GO0005095 GTPASE INHIBITOR ACTIVITY -2.656 -2.472

GO0007635 CHEMOSENSORY BEHAVIOR -2.767 -2.061

**Exclusive Gene Ontologies**

**Gene Ontology Term Z-score**

**Up regulated mTBI vs. Sham**

GO0000163 PROTEIN PHOSPHATASE TYPE 1 ACTIVITY 3.646

GO0004656 PROCOLLAGEN PROLINE 4 DIOXYGENASE ACTIVI 3.423

GO0018401 PEPTIDYL PROLINE HYDROXYLATION TO 4 HYDR 3.423

GO0004867 SERINE TYPE ENDOPEPTIDASE INHIBITOR ACTI 3.191

GO0004722 PROTEIN SERINE OR THREONINE PHOSPHATASE 3.126

GO0004322 FERROXIDASE ACTIVITY 2.990

GO0005849 MRNA CLEAVAGE FACTOR COMPLEX 2.909

GO0008191 METALLOENDOPEPTIDASE INHIBITOR ACTIVITY 2.875

GO0006412 TRANSLATION 2.863

GO0000089 MITOTIC METAPHASE 2.793

GO0030529 RIBONUCLEOPROTEIN COMPLEX 2.739

GO0006414 TRANSLATIONAL ELONGATION 2.630

GO0008396 OXYSTEROL 7 ALPHA HYDROXYLASE ACTIVITY 2.597

GO0005179 HORMONE ACTIVITY 2.549

GO0005506 IRON ION BINDING 2.527

GO0004594 PANTOTHENATE KINASE ACTIVITY 2.482

GO0044444 CYTOPLASMIC PART 2.460

GO0030033 MICROVILLUS BIOGENESIS 2.363

GO0030073 INSULIN SECRETION 2.256

GO0008199 FERRIC IRON BINDING 2.246

GO0004263 CHYMOTRYPSIN ACTIVITY 2.242

GO0050051 LEUKOTRIENE B4 20 MONOOXYGENASE ACTIVITY 2.198

GO0008514 ORGANIC ANION TRANSMEMBRANE TRANSPORTER 2.139

GO0001947 HEART LOOPING 1.929

GO0016922 LIGAND DEPENDENT NUCLEAR RECEPTOR BINDIN 1.896

GO0048251 ELASTIC FIBER ASSEMBLY 1.885

GO0005588 COLLAGEN TYPE V 1.860

GO0042541 HEMOGLOBIN BIOSYNTHETIC PROCESS 1.846

GO0043292 CONTRACTILE FIBER 1.816

GO0006800 OXYGEN AND REACTIVE OXYGEN SPECIES METAB 1.793

GO0005484 SNAP RECEPTOR ACTIVITY 1.792

GO0018298 PROTEIN CHROMOPHORE LINKAGE 1.789

GO0046902 REGULATION OF MITOCHONDRIAL MEMBRANE PER 1.736

GO0002467 GERMINAL CENTER FORMATION 1.709

GO0006997 NUCLEAR ORGANIZATION AND BIOGENESIS 1.695

GO0050700 CARD DOMAIN BINDING 1.671

GO0043275 GLUTAMATE CARBOXYPEPTIDASE II ACTIVITY 1.635

GO0008453 ALANINE GLYOXYLATE TRANSAMINASE ACTIVITY 1.632

GO0047305 (R) 3 AMINO 2 METHYLPROPIONATE PYRUVATE 1.632

GO0003873 6 PHOSPHOFRUCTO 2 KINASE ACTIVITY 1.627

GO0043297 APICAL JUNCTION ASSEMBLY 1.625

GO0008517 FOLIC ACID TRANSPORTER ACTIVITY 1.585

GO0051023 REGULATION OF IMMUNOGLOBULIN SECRETION 1.581

GO0008652 AMINO ACID BIOSYNTHETIC PROCESS 1.575

GO0009083 BRANCHED CHAIN FAMILY AMINO ACID CATABOL 1.564

GO0043149 STRESS FIBER FORMATION 1.532

GO0001750 PHOTORECEPTOR OUTER SEGMENT 1.507

GO0042562 HORMONE BINDING 1.502

**Gene Ontology Term Z-score**

**Down regulated mTBI vs. Sham**

GO0030203 GLYCOSAMINOGLYCAN METABOLIC PROCESS -1.502

GO0008605 PROTEIN KINASE CK2 REGULATOR ACTIVITY -1.504

GO0004520 ENDODEOXYRIBONUCLEASE ACTIVITY -1.516

GO0006476 PROTEIN AMINO ACID DEACETYLATION -1.528

GO0006310 DNA RECOMBINATION -1.538

GO0004003 ATP DEPENDENT DNA HELICASE ACTIVITY -1.557

GO0007271 SYNAPTIC TRANSMISSION CHOLINERGIC -1.561

GO0015012 HEPARAN SULFATE PROTEOGLYCAN BIOSYNTHETI -1.566

GO0004435 PHOSPHOINOSITIDE PHOSPHOLIPASE C ACTIVIT -1.571

GO0050730 REGULATION OF PEPTIDYL TYROSINE PHOSPHOR -1.573

GO0008080 N ACETYLTRANSFERASE ACTIVITY -1.574

GO0006044 N ACETYLGLUCOSAMINE METABOLIC PROCESS -1.609

GO0006869 LIPID TRANSPORT -1.623

GO0042473 OUTER EAR MORPHOGENESIS -1.640

GO0016075 RRNA CATABOLIC PROCESS -1.652

GO0006656 PHOSPHATIDYLCHOLINE BIOSYNTHETIC PROCESS -1.667

GO0008757 S ADENOSYLMETHIONINE DEPENDENT METHYLTRA -1.714

GO0005657 REPLICATION FORK -1.730

GO0042733 EMBRYONIC DIGIT MORPHOGENESIS -1.734

GO0006621 PROTEIN RETENTION IN ER -1.737

GO0009314 RESPONSE TO RADIATION -1.751

GO0006506 GPI ANCHOR BIOSYNTHETIC PROCESS -1.756

GO0043534 BLOOD VESSEL ENDOTHELIAL CELL MIGRATION -1.772

GO0004767 SPHINGOMYELIN PHOSPHODIESTERASE ACTIVITY -1.781

GO0045859 REGULATION OF PROTEIN KINASE ACTIVITY -1.837

GO0042474 MIDDLE EAR MORPHOGENESIS -1.849

GO0046579 POSITIVE REGULATION OF RAS PROTEIN SIGNA -1.860

GO0006333 CHROMATIN ASSEMBLY OR DISASSEMBLY -1.861

GO0051010 MICROTUBULE PLUS END BINDING -1.904

GO0043065 POSITIVE REGULATION OF APOPTOSIS -1.941

GO0005956 PROTEIN KINASE CK2 COMPLEX -1.953

GO0048015 PHOSPHOINOSITIDE MEDIATED SIGNALING -1.955

GO0004672 PROTEIN KINASE ACTIVITY -1.965

GO0042765 GPI ANCHOR TRANSAMIDASE COMPLEX -1.966

GO0019863 IGE BINDING -1.973

GO0048844 ARTERY MORPHOGENESIS -1.992

GO0008277 REGULATION OF G PROTEIN COUPLED RECEPTOR -2.003

GO0005675 HOLO TFIIH COMPLEX -2.019

GO0000075 CELL CYCLE CHECKPOINT -2.029

GO0060134 PREPULSE INHIBITION -2.031

GO0004739 PYRUVATE DEHYDROGENASE (ACETYL TRANSFERR) -2.049

GO0046907 INTRACELLULAR TRANSPORT -2.096

GO0005388 CALCIUM TRANSPORTING ATPASE ACTIVITY -2.141

GO0006270 DNA REPLICATION INITIATION -2.142

GO0007202 PHOSPHOLIPASE C ACTIVATION -2.143

GO0000166 NUCLEOTIDE BINDING -2.182

GO0042488 POSITIVE REGULATION OF ODONTOGENESIS -2.184

GO0006810 TRANSPORT -2.185

GO0008630 DNA DAMAGE RESPONSE SIGNAL TRANSDUCTION -2.203

GO0016528 SARCOPLASM -2.238

GO0005778 PEROXISOMAL MEMBRANE -2.305

GO0003924 GTPASE ACTIVITY -2.341

GO0043473 PIGMENTATION -2.352

GO0045046 PROTEIN IMPORT INTO PEROXISOME MEMBRANE -2.383

GO0006725 AROMATIC COMPOUND METABOLIC PROCESS -2.425

GO0017057 6 PHOSPHOGLUCONOLACTONASE ACTIVITY -2.433

GO0045892 NEGATIVE REGULATION OF TRANSCRIPTION DN -2.436

GO0016740 TRANSFERASE ACTIVITY -2.500

GO0005011 MACROPHAGE COLONY STIMULATING FACTOR REC -2.506

GO0030278 REGULATION OF OSSIFICATION -2.526

GO0030318 MELANOCYTE DIFFERENTIATION -2.549

GO0008324 CATION TRANSMEMBRANE TRANSPORTER ACTIVIT -2.554

GO0007399 NERVOUS SYSTEM DEVELOPMENT -2.600

GO0016055 WNT RECEPTOR SIGNALING PATHWAY -2.706

GO0005652 NUCLEAR LAMINA -2.708

GO0008094 DNA DEPENDENT ATPASE ACTIVITY -2.845

GO0001942 HAIR FOLLICLE DEVELOPMENT -2.908

GO0008535 CYTOCHROME C OXIDASE COMPLEX ASSEMBLY -2.937

GO0019001 GUANYL NUCLEOTIDE BINDING -2.938

GO0005834 HETEROTRIMERIC G PROTEIN COMPLEX -3.377

GO0004396 HEXOKINASE ACTIVITY -3.606

GO0001540 BETA AMYLOID BINDING -4.252

**Exclusive Gene Ontologies**

**Gene Ontology Term Z-score**

**Up regulated mTBI/PHEN vs. Sham**

GO0016020 MEMBRANE 4.183

GO0006754 ATP BIOSYNTHETIC PROCESS 3.434

GO0005198 STRUCTURAL MOLECULE ACTIVITY 3.325

GO0001502 CARTILAGE CONDENSATION 3.226

GO0015992 PROTON TRANSPORT 3.102

GO0009611 RESPONSE TO WOUNDING 2.875

GO0006817 PHOSPHATE TRANSPORT 2.870

GO0006958 COMPLEMENT ACTIVATION CLASSICAL PATHWAY 2.866

GO0005954 CALCIUM AND CALMODULIN DEPENDENT PROTEI 2.837

GO0005200 STRUCTURAL CONSTITUENT OF CYTOSKELETON 2.835

GO0006955 IMMUNE RESPONSE 2.743

GO0050766 POSITIVE REGULATION OF PHAGOCYTOSIS 2.670

GO0001798 POSITIVE REGULATION OF TYPE IIA HYPERSEN 2.654

GO0005391 SODIUM POTASSIUM EXCHANGING ATPASE ACTIV 2.653

GO0008553 HYDROGEN EXPORTING ATPASE ACTIVITY PHOS 2.614

GO0005624 MEMBRANE FRACTION 2.556

GO0005044 SCAVENGER RECEPTOR ACTIVITY 2.529

GO0030433 ER ASSOCIATED PROTEIN CATABOLIC PROCESS 2.524

GO0006954 INFLAMMATORY RESPONSE 2.513

GO0005952 CAMP DEPENDENT PROTEIN KINASE COMPLEX 2.421

GO0008307 STRUCTURAL CONSTITUENT OF MUSCLE 2.407

GO0005529 SUGAR BINDING 2.369

GO0019432 TRIACYLGLYCEROL BIOSYNTHETIC PROCESS 2.352

GO0005507 COPPER ION BINDING 2.319

GO0004497 MONOOXYGENASE ACTIVITY 2.317

GO0005605 BASAL LAMINA 2.293

GO0045087 INNATE IMMUNE RESPONSE 2.290

GO0016531 COPPER CHAPERONE ACTIVITY 2.261

GO0050909 SENSORY PERCEPTION OF TASTE 2.249

GO0007588 EXCRETION 2.191

GO0004602 GLUTATHIONE PEROXIDASE ACTIVITY 2.183

GO0008631 INDUCTION OF APOPTOSIS BY OXIDATIVE STRE 2.149

GO0008283 CELL PROLIFERATION 2.141

GO0005856 CYTOSKELETON 2.129

GO0005863 STRIATED MUSCLE THICK FILAMENT 2.107

GO0030286 DYNEIN COMPLEX 2.098

GO0001833 INNER CELL MASS CELL PROLIFERATION 2.030

GO0016165 LIPOXYGENASE ACTIVITY 1.984

GO0015179 L AMINO ACID TRANSMEMBRANE TRANSPORTER 1.977

GO0015807 L AMINO ACID TRANSPORT 1.977

GO0009897 EXTERNAL SIDE OF PLASMA MEMBRANE 1.976

GO0003796 LYSOZYME ACTIVITY 1.965

GO0043547 POSITIVE REGULATION OF GTPASE ACTIVITY 1.941

GO0030199 COLLAGEN FIBRIL ORGANIZATION 1.940

GO0007568 AGING 1.833

GO0008095 INOSITOL 1 4 5 TRIPHOSPHATE RECEPTOR ACT 1.770

GO0043281 REGULATION OF CASPASE ACTIVITY 1.754

GO0019370 LEUKOTRIENE BIOSYNTHETIC PROCESS 1.744

GO0008527 TASTE RECEPTOR ACTIVITY 1.741

GO0019864 IGG BINDING 1.740

GO0042554 SUPEROXIDE RELEASE 1.732

GO0005534 GALACTOSE BINDING 1.732

GO0001963 SYNAPTIC TRANSMISSION DOPAMINERGIC 1.707

GO0009209 PYRIMIDINE RIBONUCLEOSIDE TRIPHOSPHATE B 1.704

GO0046626 REGULATION OF INSULIN RECEPTOR SIGNALING 1.689

GO0031012 EXTRACELLULAR MATRIX 1.661

GO0016504 PROTEASE ACTIVATOR ACTIVITY 1.626

GO0008603 CAMP DEPENDENT PROTEIN KINASE REGULATOR 1.620

GO0004859 PHOSPHOLIPASE INHIBITOR ACTIVITY 1.601

GO0006691 LEUKOTRIENE METABOLIC PROCESS 1.578

GO0005499 VITAMIN D BINDING 1.576

GO0004052 ARACHIDONATE 12 LIPOXYGENASE ACTIVITY 1.568

GO0008367 BACTERIAL BINDING 1.564

GO0006595 POLYAMINE METABOLIC PROCESS 1.533

GO0001542 OVULATION FROM OVARIAN FOLLICLE 1.524

GO0030889 NEGATIVE REGULATION OF B CELL PROLIFERAT 1.501

**Gene Ontology Term Z-score**

**Down regulated mTBI/PHEN vs. Sham**

GO0051899 MEMBRANE DEPOLARIZATION -1.574

GO0033152 IMMUNOGLOBULIN V(D)J RECOMBINATION -1.640

GO0046668 REGULATION OF RETINAL PROGRAMMED CELL DE -1.641

GO0042054 HISTONE METHYLTRANSFERASE ACTIVITY -1.655

GO0030262 APOPTOTIC NUCLEAR CHANGES -1.655

GO0005832 CHAPERONIN CONTAINING T COMPLEX -1.728

GO0007032 ENDOSOME ORGANIZATION AND BIOGENESIS -1.748

GO0009187 CYCLIC NUCLEOTIDE METABOLIC PROCESS -1.760

GO0004437 INOSITOL OR PHOSPHATIDYLINOSITOL PHOSPHA -1.764

GO0000910 CYTOKINESIS -1.768

GO0030314 JUNCTIONAL MEMBRANE COMPLEX -1.774

GO0005385 ZINC ION TRANSMEMBRANE TRANSPORTER ACTIV -1.792

GO0048007 ANTIGEN PROCESSING AND PRESENTATION EXO -1.813

GO0007040 LYSOSOME ORGANIZATION AND BIOGENESIS -1.842

GO0003986 ACETYL COA HYDROLASE ACTIVITY -1.876

GO0022844 VOLTAGE GATED ANION CHANNEL ACTIVITY -1.898

GO0051138 POSITIVE REGULATION OF NK T CELL DIFFERE -1.903

GO0002063 CHONDROCYTE DEVELOPMENT -1.905

GO0042490 MECHANORECEPTOR DIFFERENTIATION -1.979

GO0016564 TRANSCRIPTION REPRESSOR ACTIVITY -1.982

GO0005487 NUCLEOCYTOPLASMIC TRANSPORTER ACTIVITY -1.994

GO0019861 FLAGELLUM -2.031

GO0007049 CELL CYCLE -2.095

GO0006406 MRNA EXPORT FROM NUCLEUS -2.108

GO0045651 POSITIVE REGULATION OF MACROPHAGE DIFFER -2.151

GO0046580 NEGATIVE REGULATION OF RAS PROTEIN SIGNA -2.153

GO0008312 7S RNA BINDING -2.189

GO0004749 RIBOSE PHOSPHATE DIPHOSPHOKINASE ACTIVIT -2.196

GO0048148 BEHAVIORAL RESPONSE TO COCAINE -2.197

GO0003682 CHROMATIN BINDING -2.202

GO0006379 MRNA CLEAVAGE -2.240

GO0016779 NUCLEOTIDYLTRANSFERASE ACTIVITY -2.313

GO0006164 PURINE NUCLEOTIDE BIOSYNTHETIC PROCESS -2.316

GO0046934 PHOSPHATIDYLINOSITOL 4 5 BISPHOSPHATE -2.333

GO0003677 DNA BINDING -2.336

GO0003697 SINGLE STRANDED DNA BINDING -2.350

GO0006886 INTRACELLULAR PROTEIN TRANSPORT -2.374

GO0005777 PEROXISOME -2.445

GO0030503 REGULATION OF CELL REDOX HOMEOSTASIS -2.541

GO0003676 NUCLEIC ACID BINDING -2.559

GO0001602 PANCREATIC POLYPEPTIDE RECEPTOR ACTIVITY -2.765

GO0007000 NUCLEOLUS ORGANIZATION AND BIOGENESIS -2.815

GO0065002 INTRACELLULAR PROTEIN TRANSPORT ACROSS A -2.837

GO0006446 REGULATION OF TRANSLATIONAL INITIATION -2.920

GO0016874 LIGASE ACTIVITY -3.068

GO0043161 PROTEASOMAL UBIQUITIN DEPENDENT PROTEIN -3.112

GO0050780 DOPAMINE RECEPTOR BINDING -3.140

GO0004614 PHOSPHOGLUCOMUTASE ACTIVITY -3.185

GO0008565 PROTEIN TRANSPORTER ACTIVITY -3.217

GO0042053 REGULATION OF DOPAMINE METABOLIC PROCESS -3.218

GO0048168 REGULATION OF NEURONAL SYNAPTIC PLASTICITY -3.353

GO0016868 INTRAMOLECULAR TRANSFERASE ACTIVITY PHO -3.604

GO0000059 PROTEIN IMPORT INTO NUCLEUS DOCKING -4.166

GO0000244 ASSEMBLY OF SPLICEOSOMAL TRI SNRNP -4.454

**Exclusive Gene Ontologies**

**Gene Ontology Term Z-score**

**Up regulated PHEN vs. Sham**

GO0051260 PROTEIN HOMOOLIGOMERIZATION 3.184

GO0045777 POSITIVE REGULATION OF BLOOD PRESSURE 2.697

GO0016538 CYCLIN DEPENDENT PROTEIN KINASE REGULATO 2.679

GO0007223 WNT RECEPTOR SIGNALING PATHWAY CALCIUM 2.430

GO0008035 HIGH DENSITY LIPOPROTEIN BINDING 2.357

GO0048010 VASCULAR ENDOTHELIAL GROWTH FACTOR RECEP 2.292

GO0006887 EXOCYTOSIS 2.259

GO0048468 CELL DEVELOPMENT 1.968

GO0005868 CYTOPLASMIC DYNEIN COMPLEX 1.909

GO0016772 TRANSFERASE ACTIVITY TRANSFERRING PHOSP 1.886

GO0045019 NEGATIVE REGULATION OF NITRIC OXIDE BIOS 1.868

GO0007188 G PROTEIN SIGNALING COUPLED TO CAMP NUC 1.848

GO0007611 LEARNING AND OR MEMORY 1.847

GO0046325 NEGATIVE REGULATION OF GLUCOSE IMPORT 1.774

GO0046879 HORMONE SECRETION 1.740

GO0021513 SPINAL CORD DORSAL OR VENTRAL PATTERNING 1.728

GO0008187 POLY PYRIMIDINE TRACT BINDING 1.725

GO0044445 CYTOSOLIC PART 1.698

GO0005861 TROPONIN COMPLEX 1.683

GO0016019 PEPTIDOGLYCAN RECEPTOR ACTIVITY 1.674

GO0042551 NEURON MATURATION 1.674

GO0008588 RELEASE OF CYTOPLASMIC SEQUESTERED NF KA 1.673

GO0006183 GTP BIOSYNTHETIC PROCESS 1.662

GO0006228 UTP BIOSYNTHETIC PROCESS 1.662

GO0006241 CTP BIOSYNTHETIC PROCESS 1.662

GO0008628 INDUCTION OF APOPTOSIS BY HORMONES 1.613

GO0048343 PARAXIAL MESODERMAL CELL FATE COMMITMENT 1.597

GO0006613 COTRANSLATIONAL PROTEIN TARGETING TO MEM 1.583

GO0007512 ADULT HEART DEVELOPMENT 1.583

GO0008624 INDUCTION OF APOPTOSIS BY EXTRACELLULAR 1.574

GO0048536 SPLEEN DEVELOPMENT 1.560

GO0006924 ACTIVATED T CELL APOPTOSIS 1.537

GO0045085 NEGATIVE REGULATION OF INTERLEUKIN 2 BIO 1.513

GO0050840 EXTRACELLULAR MATRIX BINDING 1.512

**Gene Ontology Term Z-score**

**Down regulated PHEN vs. Sham**

GO0005891 VOLTAGE GATED CALCIUM CHANNEL COMPLEX -1.503

GO0030127 COPII VESICLE COAT -1.506

GO0045806 NEGATIVE REGULATION OF ENDOCYTOSIS -1.514

GO0001659 THERMOREGULATION -1.526

GO0021987 CEREBRAL CORTEX DEVELOPMENT -1.589

GO0016525 NEGATIVE REGULATION OF ANGIOGENESIS -1.597

GO0015187 GLYCINE TRANSMEMBRANE TRANSPORTER ACTIVI -1.616

GO0015816 GLYCINE TRANSPORT -1.616

GO0048488 SYNAPTIC VESICLE ENDOCYTOSIS -1.618

GO0042220 RESPONSE TO COCAINE -1.626

GO0014047 GLUTAMATE SECRETION -1.640

GO0006338 CHROMATIN REMODELING -1.682

GO0001527 MICROFIBRIL -1.775

GO0043296 APICAL JUNCTION COMPLEX -1.806

GO0001892 EMBRYONIC PLACENTA DEVELOPMENT -1.814

GO0005838 PROTEASOME REGULATORY PARTICLE -1.873

GO0004197 CYSTEINE TYPE ENDOPEPTIDASE ACTIVITY -1.968

GO0001739 SEX CHROMATIN -2.044

GO0005977 GLYCOGEN METABOLIC PROCESS -2.045

GO0045735 NUTRIENT RESERVOIR ACTIVITY -2.113

GO0004813 ALANINE TRNA LIGASE ACTIVITY -2.115

GO0006419 ALANYL TRNA AMINOACYLATION -2.115

GO0009791 POST EMBRYONIC DEVELOPMENT -2.153

GO0048169 REGULATION OF LONG TERM NEURONAL SYNAPTIC PLASTICITY -2.217

GO0048167 REGULATION OF SYNAPTIC PLASTICITY -2.265

GO0006906 VESICLE FUSION -2.331

GO0005923 TIGHT JUNCTION -2.403

GO0043542 ENDOTHELIAL CELL MIGRATION -2.440

GO0030512 NEGATIVE REGULATION OF TRANSFORMING GROW -2.462

GO0001955 BLOOD VESSEL MATURATION -2.705

GO0007195 DOPAMINE RECEPTOR ADENYLATE CYCLASE INH -2.848

GO0050220 PROSTAGLANDIN E SYNTHASE ACTIVITY -2.934

GO0043433 NEGATIVE REGULATION OF TRANSCRIPTION FAC -2.996

GO0030118 CLATHRIN COAT -3.053

GO0006950 RESPONSE TO STRESS -3.521
