# Supplementary material for: Cognitive Impairments Induced by Concussive Mild Traumatic Brain Injury in Mouse Are Ameliorated by Treatment with Phenserine via Multiple Non-Cholinergic and Cholinergic Mechanisms
Source: PLoS One. 2016 Jun 2;11(6):e0156493. doi: 10.1371/journal.pone.0156493 (PMC4890804; doi:10.1371/journal.pone.0156493)
Supplement: S3 Table — (DOCX) [file pone.0156493.s003.docx]

**Supplemental Table illustrating the identities of significantly regulated canonical pathways indicated in Figure 4C**

**R* refers to “Reactome”**

**Common Pairwise regulated pathways from Figure 4C**

**Pathway - mTBI vs. Sham & mTBI/PHEN vs. Sham**  **mTBI vs. Sham mTBI/PHEN vs. Sham**

**DOWN REGULATED Z-score Z-score**

R* PYRUVATE METABOLISM AND TCA CYCLE -2.936 -3.224

R* TRANSPORT OF RIBONUCLEOPROTEINS INTO THE HOST NUCLEUS -2.485 -2.365

KEGG CITRATE CYCLE TCA CYCLE -2.296 -3.492

R* TRANSFORMATION OF LANOSTEROL TO CHOLESTEROL -2.004 -2.214

R* SIGNALING BY ROBO RECEPTOR -1.792 -2.685

**UP REGULATED Z-score Z-score**

KEGG ALLOGRAFT REJECTION 1.134 1.438

R* CYCLIN A1 ASSOCIATED EVENTS DURING G2 M TRANSITION 1.536 1.672

BIOCARTA IL5 PATHWAY 1.879 1.171

KEGG GRAFT VERSUS HOST DISEASE 1.936 1.583

KEGG COMPLEMENT AND COAGULATION CASCADES 2.074 2.029

KEGG AUTOIMMUNE THYROID DISEASE 2.154 1.194

KEGG PANTOTHENATE AND COA BIOSYNTHESIS 2.621 1.427

R* METABOLISM OF PROTEINS 3.955 3.747

R* FORMATION OF THE TERNARY COMPLEX AND SUBSEQUENTLY THE 43S COMPLEX 5.137 5.346

R* GTP HYDROLYSIS AND JOINING OF THE 60S RIBOSOMAL SUBUNIT 5.718 5.090

R* INSULIN SYNTHESIS AND SECRETION 5.802 5.096

R* REGULATION OF BETA CELL DEVELOPMENT 5.806 5.462

R* REGULATION OF GENE EXPRESSION IN BETA CELLS 5.825 6.023

R* TRANSLATION 6.007 5.178

R* INFLUENZA VIRAL RNA TRANSCRIPTION AND REPLICATION 6.165 5.476

R* FORMATION OF A POOL OF FREE 40S SUBUNITS 6.537 6.193

R* VIRAL MRNA TRANSLATION 6.567 5.781

R* PEPTIDE CHAIN ELONGATION 6.622 6.268

KEGG RIBOSOME 6.626 6.249

**Pathway - mTBI/PHEN vs. Sham & PHEN vs. Sham mTBI/PHEN vs. Sham PHEN vs. Sham**

**DOWN REGULATED Z-score Z-score**

R* RNA POLYMERASE I PROMOTER OPENING -4.333 -6.257

R* PACKAGING OF TELOMERE ENDS -4.046 -6.147

R* RNA POLYMERASE I PROMOTER CLEARANCE -3.964 -5.691

KEGG SYSTEMIC LUPUS ERYTHEMATOSUS -3.680 -3.813

R* APOPTOTIC EXECUTION PHASE -2.948 -2.915

**UP REGULATED Z-score Z-score**

R* GAMMA CARBOXYLATION TRANSPORT AND AMINO TERMINAL CLEAVAGE OF PROTEINS 1.354 1.155

R* NUCLEAR RECEPTOR TRANSCRIPTION PATHWAY 1.952 1.514

R* STRIATED MUSCLE CONTRACTION 2.266 1.761

BIOCARTA RARRXR PATHWAY 2.698 1.336

KEGG HEDGEHOG SIGNALING PATHWAY 3.685 2.836

**Pathway - mTBI vs. Sham & PHEN vs. Sham mTBI vs. Sham PHEN vs. Sham**

**DOWN REGULATED Z-score Z-score**

KEGG GLYCEROPHOSPHOLIPID METABOLISM -2.837 -2.928

R* TRIACYLGLYCERIDE BIOSYNTHESIS -2.613 -3.252

**UP REGULATED Z-score Z-score**

BIOCARTA PARKIN PATHWAY 1.343 1.496

**Exclusively regulated pathways from Venn Diagram**

**Pathway - mTBI vs. Sham mTBI vs. Sham**

**DOWN REGULATED Z-score**

R* DNA REPAIR -3.422

R* HDL MEDIATED LIPID TRANSPORT -3.295

R* NEUROTRANSMITTER RECEPTOR BINDING AND DOWNSTREAM TRANSMISSION IN THE POSTSYNAPTIC CELL -3.061

R* SIGNALLING TO RAS -2.978

KEGG AMINO SUGAR AND NUCLEOTIDE SUGAR METABOLISM -2.976

R* TRANSMISSION ACROSS CHEMICAL SYNAPSES -2.940

R* DNA STRAND ELONGATION -2.880

R* DUAL INCISION REACTION IN GG NER -2.858

R* GLOBAL GENOMIC NER -2.829

KEGG AXON GUIDANCE -2.762

R* METABOLISM OF RNA -2.761

KEGG NUCLEOTIDE EXCISION REPAIR -2.757

R* TRANSMEMBRANE TRANSPORT OF SMALL MOLECULES -2.755

KEGG DNA REPLICATION -2.743

KEGG PANCREATIC CANCER -2.727

R* GLUCOSE TRANSPORT -2.643

R* UNWINDING OF DNA -2.629

R* NUCLEOTIDE EXCISION REPAIR -2.486

R* REGULATION OF GLUCOKINASE BY GLUCOKINASE REGULATORY PROTEIN -2.456

ST PHOSPHOINOSITIDE 3 KINASE PATHWAY -2.442

R* NUCLEAR IMPORT OF REV PROTEIN -2.436

R* REGULATION OF LIPID METABOLISM BY PEROXISOME PROLIFERATOR ACTIVATED RECEPTOR ALPHA -2.407

R* DEPOLARIZATION OF THE PRESYNAPTIC TERMINAL TRIGGERS THE OPENING OF CALCIUM CHANNELS -2.366

ST INTEGRIN SIGNALING PATHWAY -2.353

R* NEP NS2 INTERACTS WITH THE CELLULAR EXPORT MACHINERY -2.290

R* ACTIVATION OF ATR IN RESPONSE TO REPLICATION STRESS -2.277

BIOCARTA CERAMIDE PATHWAY -2.190

KEGG MISMATCH REPAIR -2.185

R* VPR MEDIATED NUCLEAR IMPORT OF PICS -2.146

R* REV MEDIATED NUCLEAR EXPORT OF HIV1 RNA -2.099

R* SNRNP ASSEMBLY -1.937

BIOCARTA AGR PATHWAY -1.869

R* TRAFFICKING OF GLUR2 CONTAINING AMPA RECEPTORS -1.584

**Up regulated Z-score**

R* XENOBIOTICS 1.287

BIOCARTA ACE2 PATHWAY 1.755

BIOCARTA AMI PATHWAY 1.761

BIOCARTA PLATELETAPP PATHWAY 1.829

BIOCARTA INTRINSIC PATHWAY 1.972

R* PP2A MEDIATED DEPHOSPHORYLATION OF KEY METABOLIC FACTORS 2.027

R* DARPP32 EVENTS 2.864

R* DIABETES PATHWAYS 3.955

R* INFLUENZA LIFE CYCLE 4.163

**Pathway - mTBI/PHEN vs. Sham mTBI/PHEN vs. Sham**

**DOWN REGULATED Z-score**

R* RNA POLYMERASE I III AND MITOCHONDRIAL TRANSCRIPTION -5.756

R* TRANSCRIPTION -5.491

R* TELOMERE MAINTENANCE -4.361

R* REGULATION OF PYRUVATE DEHYDROGENASE COMPLEX -3.419

KEGG PEROXISOME -3.106

R* PYRUVATE METABOLISM -3.037

BIOCARTA EIF4 PATHWAY -2.838

R* ASSOCIATION OF TRIC CCT WITH TARGET PROTEINS DURING BIOSYNTHESIS -2.637

R* TCR SIGNALING -2.454

KEGG UBIQUITIN MEDIATED PROTEOLYSIS -2.349

KEGG PROTEIN EXPORT -2.221

R* ZINC TRANSPORTATION -2.187

R* PURINE RIBONUCLEOSIDE MONOPHOSPHATE BIOSYNTHESIS -2.128

R* ZINC INFLUX INTO CELLS BY THE SLC39 GENES FAMILY -1.977

R* DOWNSTREAM TCR SIGNALING -1.977

**UP REGULATED Z-score**

SA G2 AND M PHASES 0.815

BIOCARTA ASBCELL PATHWAY 0.935

KEGG PRIMARY BILE ACID BIOSYNTHESIS 1.186

BIOCARTA TALL1 PATHWAY 1.193

BIOCARTA NUCLEARRS PATHWAY 1.354

R* SIGNAL ATTENUATION 1.558

SA G1 AND S PHASES 1.568

SIG BCR SIGNALING PATHWAY 1.693

BIOCARTA DC PATHWAY 2.171

R* G2 M TRANSITION 2.200

BIOCARTA STATHMIN PATHWAY 2.342

R* GPCR LIGAND BINDING 2.394

ST WNT CA2 CYCLIC GMP PATHWAY 2.483

R* CLASS A1 RHODOPSIN LIKE RECEPTORS 2.539

KEGG ECM RECEPTOR INTERACTION 2.944

R* NCAM1 INTERACTIONS 2.992

**Pathway - PHEN vs. Sham PHEN vs. Sham**

**DOWN REGULATED Z-score**

KEGG OOCYTE MEIOSIS -3.125

BIOCARTA TCRA PATHWAY -1.406

R* TRANSLOCATION OF ZAP70 TO IMMUNOLOGICAL SYNAPSE -1.247

**UP REGULATED** **Z-score**

SA REG CASCADE OF CYCLIN EXPR 1.892

R* METABOLISM OF VITAMINS AND COFACTORS 2.091

KEGG TASTE TRANSDUCTION 2.191
